# Exosomal misfolded α1-antitrypsin triggers a cytosolic GRP78-dependent unfolded protein response and pro-survival signaling in pre-metastatic tissues

**DOI:** 10.1016/j.jbc.2026.113282

**Published:** 2026-06-20

**Authors:** Anuran Bhattacharya, Tiyasa Saha, Aditya Shukla, Ananya Banerjee, Himansu Roy, Urmi Chatterji

**Affiliations:** 1 Cancer Research Laboratory, Department of Zoology, University of Calcutta, Kolkata, India; 2 Cell Biology Laboratory, Department of Microbiology, University of Calcutta, Kolkata, India; 3 Department of General Surgery, KPC Medical and College and Hospital, Kolkata, India; 4 Centre for Research in Nanoscience and Nanotechnology, University of Calcutta, Kolkata, India

**Keywords:** tumor-derived exosomes, misfolded α1-antitrypsin, unfolded protein response, GRP78, stemness, ER stress, inflammation

## Abstract

Tumor-derived exosomes (TDEs) promote cancer progression by transmitting oncogenic signals to adjacent cells. However, their impact on distant, non-malignant tissues remains poorly defined, particularly with respect to their bioactive cargo. Subsequently, a misfolded form of α1-antitrypsin (mA1AT) was identified as an exosomal component secreted by 4T1 mice mammary tumor cells, and validated in breast cancer patient serum. Exosome-mediated delivery of mA1AT to healthy lung, liver and bone marrow cells, prospective sites for metastasis, induces cytoplasmic expression of GRP78, triggers a pro-survival unfolded protein response (UPR), activates proliferation and inflammation, while suppressing apoptosis, both *in vitro* and *in vivo*. In silico modeling and co-immunoprecipitation confirm interaction between GRP78 and mA1AT, implicating non-canonical UPR activation. Depletion of mA1AT from exosomes reduces these effects, signifying its role in exosome-mediated transfer of oncogenic traits to distant, non-malignant tissues, and highlighting its potential as a therapeutic target to limit systemic dissemination of tumorigenic cues.

Tumor-derived exosome-borne misfolded A1AT confers tumorigenic traits to healthy organs prone to metastasis, prior to arrival of circulating tumor cells, by enhancing proliferation, inflammation and activating cellular stress *via* increased interaction with GRP78 in the cytosol. Misfolded A1AT can be developed as a diagnostic and therapeutic molecule for tumor metastasis.

Cell-to-cell communication is fundamental for maintaining homeostasis and coordinating growth, differentiation, maintenance, and organ formation in multicellular organisms (1). However, when gone awry under stress, it can lead to debilitating changes, culminating in various diseases and disorders. Communication can occur *via* – (i) direct contact, (ii) secretory signaling molecules, such as hormones, growth factors and cytokines, or (iii) macromolecules enclosed within extracellular vesicles (EVs), especially exosomes (1). The versatility of the exosomes and their bioactive components has ever since incited their applications in both diagnostics and therapeutics (2), since they can radically manipulate the function of neighboring and distant cells. While normal cells rely on these mechanisms to regulate processes like immune responses, tissue repair and metabolic balance, cancer cells apprehend these communication modalities to promote tumor growth, immune evasion and metastasis (3). Subsequently, tumor-derived exosomes (TDEs) play essential roles in metastasis by reprogramming recipient cells to support a pre-metastatic niche formation, prior to invasion by circulating tumor cells. Clinically, exosomes may therefore serve as tumor biomarkers and novel therapeutic targets for predicting and preventing metastasis (4).

Traditionally, metastasis has been attributed to the migration and invasion of tumor cells from the primary site to secondary organs (5). While some organs like the lungs, liver, and bone marrow are more vulnerable than others, it has been advocated that they need to be primed for accepting the invading tumor cells. Accordingly, the “seed and soil” hypothesis of Paget, 1889 (6) projected that circulating tumor cells (‘seeds’) from primary breast tumors preferentially colonize to specific organs (‘soil’), which are permissive to tumor growth. Recent evidence suggests that tumors themselves prepare distant tissues for colonization by altering their microenvironment prior to the arrival of cancer cells. Preparation of this "pre-metastatic niche" involves a series of molecular and cellular changes in the secondary organs, orchestrated by factors released from the primary tumor (7). Tumor-secreted components, such as soluble proteins, cytokines, and exosomes play pivotal roles in shaping the pre-metastatic niche (8), even in the absence of direct cell migration (9). In this perspective, exosome-borne components may serve as key mediators (“fertilizers”) for efficient niche preparation in cancer metastasis, since they are highly stable in circulation, enabling them to travel significant distances and target specific cells (10). Once internalized, their cargo can modulate gene expression, protein synthesis and cellular behavior, rendering them as powerful modulators of the tumor microenvironment and systemic tumor progression (11).

Intriguingly, TDEs often induce endoplasmic reticulum (ER) stress in recipient cells (12), specifically when misfolded proteins accumulate in the ER, activating the unfolded protein response (UPR), a cellular program designed to restore proteostasis (13, 14). In support, studies have shown that TDEs can transmit ER stress from primary tumors to adjacent normal cells, driving tumor-promoting changes within the microenvironment (15). While transient ER stress is protective, chronic or unresolved ER stress can induce tumorigenic processes (16), including enhanced tumor cell survival and proliferation (17), immune modulation (18) and stemness (19). Despite these advances, the role of TDEs and their components, specifically misfolded proteins, in inducing ER stress and transferring oncogenic traits from primary tumors to healthy tissues is yet to be ascertained. In this study, we have attempted to identify specific exosomal constituents responsible for distant metastasis, establishing their minimally invasive diagnostic and therapeutic potential.

## Results

### TDEs induce oncogenic traits at putative metastatic sites

Mouse mammary 4T1 cells were orthotopically injected into BALB/c mice and allowed to develop for 7, 14 and 21 days. Tumor burden progressively increased, accompanied by indications of cellular atypia and stromal infiltration (Fig. S1, *A*–*C*). Exosomes isolated from control and tumor-bearing mice serum expressed canonical exosomal markers (CD63, CD81, TSG101; Fig. S1*D*, S27A), but were significantly negative for the non-exosomal contaminant albumin, confirming the purity of the isolated fractions (Fig. S1*E*). Furthermore, the vesicles displayed a uniform size range (50–150 nm; Fig. S1*F*) and exhibited typical spherical morphology (Fig. S1*G*).

**Figure 1:**
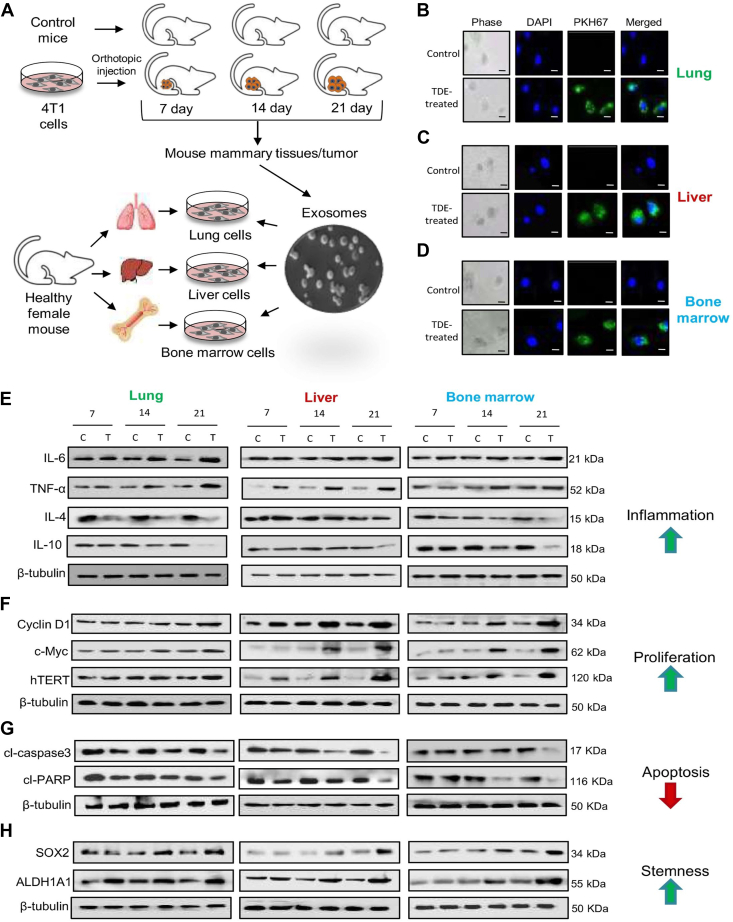
**TDEs induce oncogenic traits in normal cells at potential metastatic sites.***A*, schematic illustration of the experimental workflow. Female BALB/c mice were inoculated with 1 × 10^4^ 4T1 cells and allowed to develop tumors for 7, 14 and 21 days, while respective control groups received vehicle control treatment for the same duration (n = 3). After the incubation period, exosomes were isolated from the serum of mammary tumor–bearing mice (TDEs) and control, and subsequently used to treat primary lung, liver and bone marrow cells isolated and cultured from healthy female BALB/c mice. *B–D*, confocal microscopic images of primary lung (*B*), liver (*C*) and bone marrow (*D*) cells showing uptake of PKH67-labeled exosomes (*green*); nuclei are counterstained with DAPI (*blue*). Exosome-depleted supernatant (PKH67-labeled) was used as control. Magnification, 40X; scale bar, 20 μm. *E–H*, immunoblot analyses of lung, liver and bone marrow cells following treatment with control exosomes (C) or TDEs (T), assessing (*E*) inflammatory markers (IL-6, TNF-α, IL-4, IL-10; n = 3), (*F*) proliferation markers (cyclin D1, cMyc, hTERT; n = 3), (*G*) apoptotic markers (cleaved caspase 3, cleaved PARP; n = 3) and (*H*) stem cell markers (SOX2, ALDH1A1; n = 3). Protein expressions were normalized against β-tubulin as the internal loading control. Data are presented as mean ± SD (n = 3). Corresponding quantitative graphs with individual data points are provided in Figs. S4 and S5. Statistical significance was determined by two-way ANOVA followed by Tukey’s multiple-comparison test. *p* < 0.05 was considered significant. Different letters (a–d) denote statistically significant differences between groups.

Next, isolated exosomes from control and tumor-bearing (TDEs) mice were administered to healthy liver, lung and bone marrow cells, plausible organs of metastasis. A schematic representation of the experimental workflow is shown in Figure 1*A*. PKH67-labeling confirmed efficient uptake of TDEs by primary lung, liver and bone marrow cells, with perinuclear localization, compared to cells treated with exosome-depleted supernatant (Fig. 1, *B*–*D*).

Exosome internalization elicited marked molecular reprogramming in recipient cells. Pro-inflammatory cytokines (IL-6, TNF-α) were significantly upregulated, whereas anti-inflammatory mediators (IL-4, IL-10) were reduced (Fig. 1*E*). Concomitantly, proliferation markers like cyclin D1, c-Myc, and hTERT were elevated (Fig. 1*F*), while apoptosis indicators cleaved caspase-3 and cleaved PARP were suppressed (Fig. 1*G*). Furthermore, stemness-associated factors SOX2 and ALDH1A1 were significantly upregulated in exosome-treated cells (Fig. 1*H*). Individual data points, along with their respective statistical quantifications, are shown in Figs. S4 and S5.

### TDEs induce stemness and ER stress in healthy tissues

**Figure 2:**
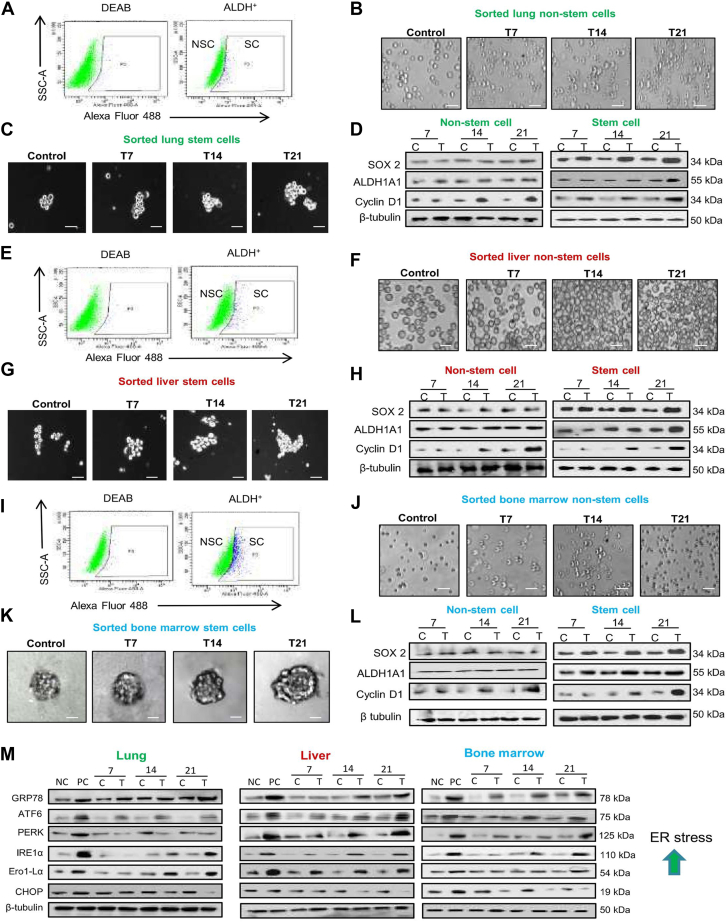
**TDEs enhance stemness and ER stress in healthy cells of lung, liver and bone marrow.** ALDEFLUOR assay and gating strategy for isolation of ALDH^+^ (stem cell) and ALDH^-^ (non-stem cell) fractions from (*A*) lungs, (*E*) liver and (*I*) bone marrow of healthy BALB/c mice. DEAB-treated cells served as negative control for ALDEFLUOR staining. Phase-contrast images of sorted ALDH^-^ (*B*, *F*, and *J*) and ALDH^+^ (*C*, *G*, and *K*) lung, liver and bone marrow cells, respectively, after treatment with TDEs (T) or control exosomes (C) for 5 days. Western blot analysis of SOX2, ALDH1A1, and cyclin D1 in ALDH^+^ and ALDH^-^ (*D*) lung, (*H*) liver and (*L*) bone marrow cells treated with TDEs or control exosomes (n = 3). *M*, Western blot analyses of whole-cell lysates from lung, liver and bone marrow cells, treated with control exosomes (*C*) or TDEs (T), and probed for UPR markers (GRP78, ATF6, PERK, IRE1α, Ero1-Lα, CHOP; n = 3). DTT-treated cells served as positive control (PC) and untreated healthy cells served as negative control (NC). All protein expressions were normalized against β-tubulin as the internal loading control. Data are presented as mean ± SD (*n* = biological replicates per group). Corresponding quantitative graphs with individual data points are provided in Figs. S6–S8. Statistical significance was determined by two-way ANOVA followed by Tukey’s multiple-comparison test. *p* < 0.05 was considered significant. Different letters (a–d) denote statistically significant differences between groups.

To assess the impact of TDEs on stem and non-stem compartments, ALDH^+^ (stem cell) and ALDH^-^ (non-stem cell) fractions were isolated from lung, liver and bone marrow of healthy BALB/c mice (Fig. 2, *A*, *E*, and *I*, respectively). In lung-derived cells, ALDH^-^ fractions displayed elevated mitotic activity (Fig. 2*B*), whereas ALDH^+^ fractions formed spheroid-like aggregates following TDE exposure (Fig. 2*C*). Immunoblotting revealed that ALDH^+^ lung cells exhibited upregulated SOX2, ALDH1A1 and cyclin D1 (Fig. 2*D*), indicating enhanced self-renewal. A similar pattern was observed in the liver (Fig. 2, *F*–*H*) and bone marrow (Fig. 2, *J*–*L*), where TDE-treated ALDH^+^ fractions exhibited increased stemness and proliferation in comparison to ALDH^-^ fractions. Simultaneously, TDEs increased expressions of ER stress markers GRP78, ATF4, PERK, IRE1α and Ero1-Lα with a concomitant reduction in CHOP in healthy lung, liver and bone marrow cells (Fig. 2*M*). Furthermore, increased expressions of downstream ER stress markers XBP-1s and ATF4 upon TDE treatment in healthy lung, liver and bone marrow cells confirmed ER stress activation (Fig. S1*H*). Individual data points, along with their respective statistical quantifications, are shown in Figs. S6–S8 and S27, *B* and *C*.

### Misfolded A1AT reigns abundant in TDE-borne cargo

**Figure 3:**
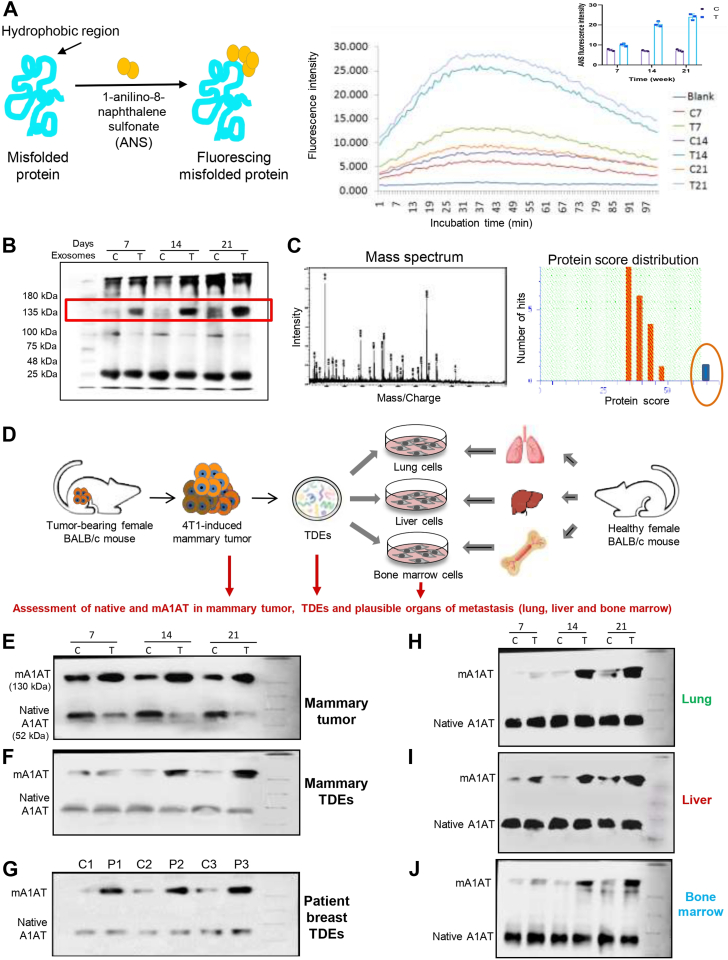
**TDEs exhibit progressively increasing levels of misfolded A1AT (mA1AT).***A*, analysis of exosomal misfolded protein abundance using ANS dye fluorescence spectroscopy. Fluorescence intensity values were plotted across the 450 to 550 nm range to assess the progressive accumulation of misfolded proteins (n = 3). *B*, Coomassie-stained native gel of exosomal proteins isolated from the serum of control (C) and tumor-bearing mice (T). Differentially expressed bands were selected for mass spectrometry analysis. *C*, MALDI-TOF MS and tandem MS/MS analysis of the selected band from the native gel, confirming the identity of the protein (circled in orange) as α1-antitrypsin (A1AT). *D*, schematic representation illustrating the assessment of native and mA1AT in mammary tumor, TDEs and plausible organs of metastasis (lung, liver and bone marrow). Western blot analyses of both native and mA1AT in (*E*) mammary fat pad (C) and tumor (T); (*F*) exosomal protein isolated from control (C) and tumor-bearing (T) mice (n = 3), and (*G*) exosomal protein isolated from healthy age-matched control (C) and triple negative breast cancer patients (T; n = 6). Native and mA1AT were assessed in (*H*) lung, (*I*) liver and (*J*) bone marrow cells, isolated from healthy female BALB/c mice, and treated with control exosomes (C) and TDEs (T). Data are presented as mean ± SD (*n* = biological replicates per group). Corresponding quantitative graphs with individual data points are provided in Fig. S9. Statistical significance was determined by two-way ANOVA followed by Tukey’s multiple-comparison test. *p* < 0.05 was considered significant. Different letters (a–d) denote statistically significant differences between groups.

Since TDEs were found to induce ER stress in healthy cells, we next examined if misfolded proteins, potent inducers of ER stress, were enriched within the exosomal cargo. Increasing ANS fluorescence intensity indicated the prevalence of misfolded proteins within the exosomal cargo. ANS intensity increased progressively with tumor advancement, and the most significant elevation was observed in exosomes from 21-day tumor-bearing mice (Fig. 3*A*).

Next, to identify the specific misfolded cargo, serum exosomes from control and tumor-bearing mice were first analyzed by native PAGE. A distinctly high-molecular-weight band (∼130 kDa), significantly low in the control exosomes (C), was consistently observed in the TDE (T) samples (Fig. 3*B*). The excised band was subjected to MALDI-TOF and MS/MS analysis and identified as α1-antitrypsin (A1AT), a 52 kDa serine protease inhibitor prone to pathological misfolding under ER stress (Fig. 3*C*). The ∼130 kDa band, therefore, suggests conformationally altered or misfolded form of A1AT (mA1AT). Assessment of the native and mA1AT was performed in mice tumors, TDEs and TDE-administered healthy lung, liver and bone marrow cells (Fig. 3*D*). Validation by immunoblotting with a conformation-independent anti-A1AT antibody, capable of detecting both native and misfolded forms, confirmed that mA1AT was enriched in primary mammary tumor lysates as well as serum-derived exosomes from tumor-bearing mice (TDEs; Fig. 3, *E* and *F*). Exosomes derived from serum of three representative age-matched control and triple-negative breast cancer patients showed a similar trend in mA1AT expression (Fig. 3*G*). Although the present study does not include direct structural characterization of misfolded A1AT, multiple complementary approaches, including ANS fluorescence, native PAGE analysis, mass spectrometric identification and conformation-sensitive immunodetection consistently supports the presence of a structurally altered form of A1AT, which provides strong evidence of protein misfolding. Protease protection analysis of representative exosome samples revealed that mA1AT remained resistant to proteinase K digestion under intact conditions, whereas detergent-mediated membrane disruption resulted in substantial loss of the signal, supporting its intraluminal localization within tumor-derived exosomes (Fig. S2*I*). Moreover, primary lung, liver and bone marrow cells from healthy BALB/c mouse, exposed to TDEs, showed intracellular accumulation of mA1AT, substantiating horizontal transfer of this misfolded protein into recipient tissues (Fig. 3, *H*–*J*). Individual data points, along with their respective statistical quantifications, are shown in Fig. S9.

### Depletion of mA1AT from TDEs reduces tumorigenic traits in healthy recipient cells

**Figure 4:**
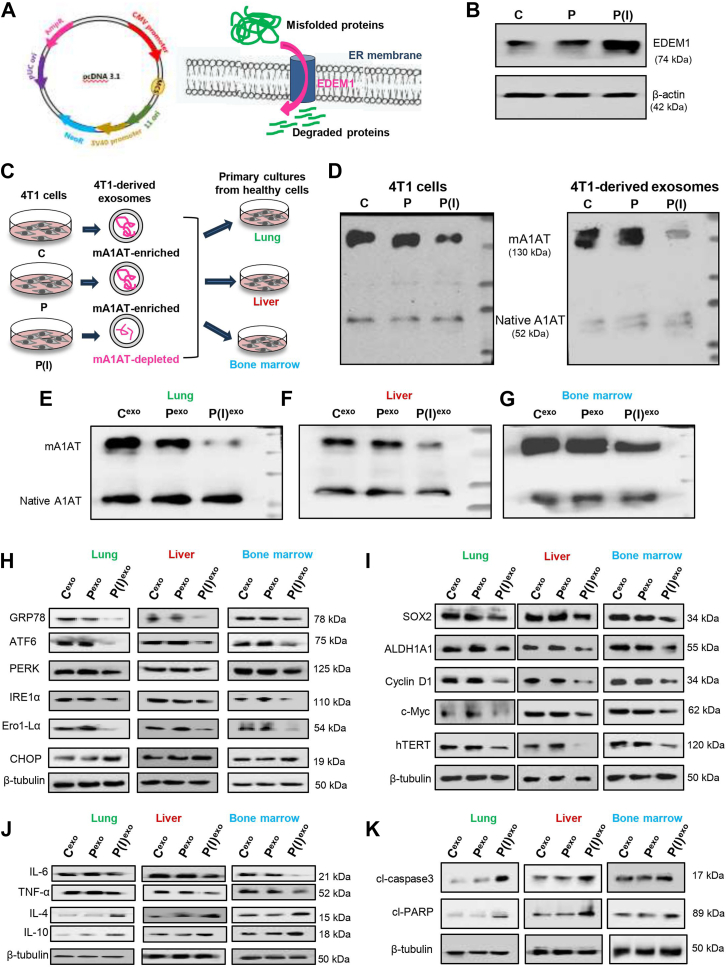
**Depletion of mA1AT in TDEs reduces tumorigenic traits in recipient normal cells.***A*, schematic representation of the pcDNA3.1 vector used for EDEM1 overexpression in 4T1 cells and its effects on misfolded proteins. *B*, Western blot analysis confirming successful EDEM1 overexpression. EDEM1 overexpression was normalized against β-actin. *C*, schematic representation of the experimental design showing treatment of primary lung, liver and bone marrow cells isolated from healthy BALB/c mice with either mA1AT-enriched or depleted exosomes. *D*, Western blot following native gel electrophoresis showing expressions of native and mA1AT in whole-cell lysates of control (C), empty vector–transfected (P) and EDEM1-overexpressing (P(I) 4T1 cells and exosomes derived from their conditioned media. Expressions of native A1AT and mA1AT following native gel electrophoresis of lung (*E*), liver (*F*) and bone marrow (*G*) cells, treated with mA1AT-enriched or depleted exosomes. Western blot analysis of lung, liver and bone marrow cells treated with mA1AT–enriched or –depleted exosomes, assessing (*H*) UPR markers (GRP78, ATF6, PERK, IRE1α, Ero1-Lα, CHOP), (*I*) stemness and proliferation markers (SOX2, ALDH1A1, cyclin D1, c-Myc, hTERT), (*J*) inflammatory markers (IL-6, TNF-α, IL-4, IL-10) and (*K*) apoptotic markers (cleaved caspase-3, cleaved PARP). Protein expressions were normalized against β-tubulin as the internal loading control. All experiments were performed at least three times. Data are presented as mean ± SD (*n* = 3). Corresponding quantitative graphs with individual data points are provided in Figs. S10–S13. Statistical significance was determined by one-way/two-way ANOVA followed by Tukey’s multiple-comparison test. *p* < 0.05 was considered significant. Different letters (a,b) denote statistically significant differences between groups.

To determine whether mA1AT is responsible for TDE-mediated tumorigenic reprogramming, EDEM1 was overexpressed in 4T1 cells to enhance ER-associated degradation of mA1AT (Fig. 4*A*) and overexpression was confirmed by Western blot (Fig. 4*B*). Exosomes isolated from the conditioned media were characterized by DLS (Fig. S2*A*) and SEM (Fig. S2*B*). Their identity was further confirmed *via* Western blot analysis for canonical exosomal markers (Figs. S2*C* and S27*D*) and the absence of non-exosomal contaminants (Fig. S2*D*). Nanoparticle tracking analysis (NTA) demonstrated comparable exosome particle concentrations amongst the experimental groups, indicating that total vesicle secretion was not markedly altered under specific experimental conditions (Fig. S2*E*). 4T1 cells were treated without or with empty plasmids and plasmids containing EDEM1 (Fig. 4*C*). Native PAGE–immunoblotting demonstrated significant depletion of mA1AT in both EDEM1-overexpressing 4T1 cells and exosomes from EDEM1-overexpressing cells, without affecting expression of the native form of A1AT (Fig. 4*D*).

Primary lung, liver and bone marrow cells were treated with both mA1AT-enriched and mA1AT-depleted exosomes (Fig. 4*C*) and were efficiently internalized in the healthy cells (Fig. S2, *F*–*H*). However, healthy cells exposed to mA1AT-depleted exosomes displayed markedly reduced intracellular accumulation of mA1AT, while native A1AT remained unchanged (Fig. 4, *E*–*G*).

Interestingly, mA1AT-depleted exosomes failed to induce tumorigenic traits in healthy cells. Lung, liver, and bone marrow cells exhibited reduced expressions of GRP78, ATF6, PERK, IRE1α, ERO-1Lα (Fig. 4*H*), concomitant with decreased expression of downstream UPR markers (XBP-1s and ATF4; Fig. S2*J*), downregulation of stemness markers (SOX2, ALDH1A1; Fig. 4*I*), proliferation markers (cyclin D1, c-Myc, hTERT; Fig. 4*I*), suppression of pro-inflammatory cytokines (IL-6, TNF-α) along with an increase in IL-4 and IL-10 expressions (Fig. 4*J*). Notably, pro-apoptotic CHOP (Fig. 4*H*), cleaved caspase-3 and cleaved PARP (Fig. 4*K*) were upregulated, indicating a shift toward cell death. Individual data points, and their respective statistical quantifications, are shown in Figs. S10–S13, and S27, *E* and *F*.

### TDE-mediated transfer of mA1AT enhances tumor burden and inflammation *in vivo*

**Figure 5:**
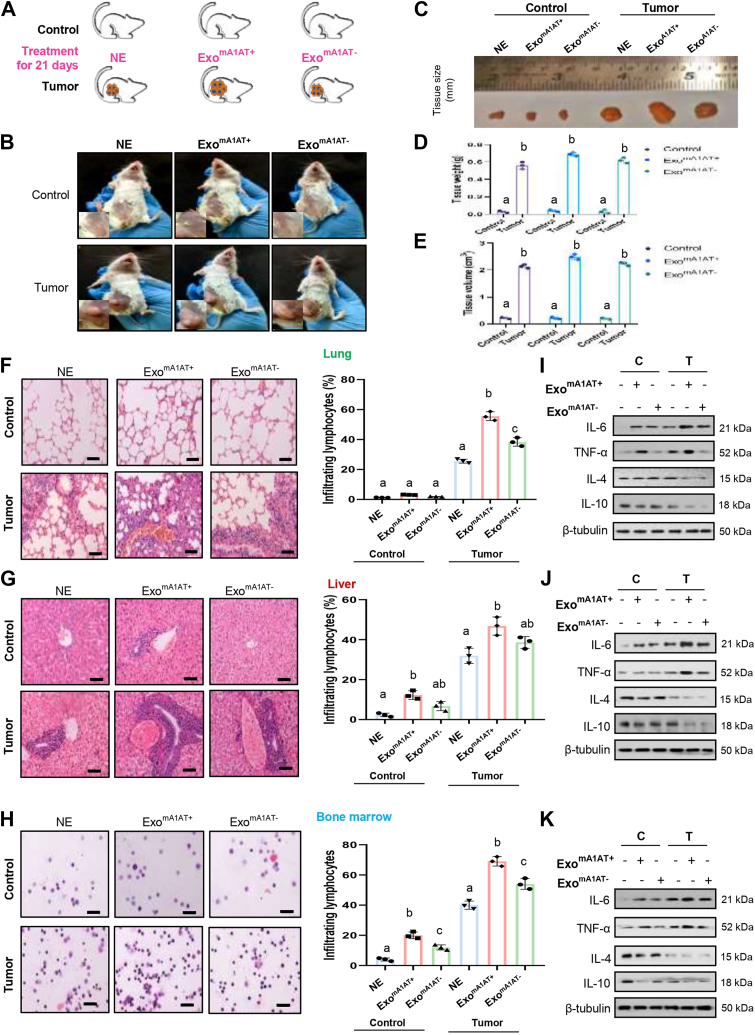
**TDE-borne mA1AT enhances tumor burden and inflammation *in vivo*.***A*, schematic representation of the exosome education model in BALB/c mice, showing systemic administration of vehicle control (NE), mA1AT–enriched (Exo^mA1AT+^) or mA1AT–depleted (Exo^mA1AT-^) exosomes into non–tumor-bearing (Control) and tumor-bearing (Tumor) mice. Representative images of control and tumor-bearing mice (*B*) and dissected mammary fat pad and tumors (*C*) from NE, Exo^mA1AT+^ or Exo^mA1AT-^ treated groups. *D* and *E*, quantification of mammary fat pad and tumor weight (*D*), and tumor volume (*E*) across experimental groups. Representative hematoxylin and eosin-stained sections and quantification of lymphocytic infiltration in lung (*F*), liver (*G*) and bone marrow (*H*) from control and tumor-bearing mice treated with NE, Exo^mA1AT+^ or Exo^mA1AT-^. Magnification, 20X; scale bar, 50 μm. Western blot analysis of whole-tissue lysates from lung (*I*), liver (*J*) and bone marrow (*K*) of control and tumor-bearing mice treated with NE, Exo^mA1AT+^ or Exo^mA1AT-^, and probed for IL-6, TNF-α, IL-4 and IL-10. All protein expressions were normalized against β-tubulin as internal loading control. Data are presented as mean ± SD (*n* = biological replicates per group). Corresponding quantitative graphs of the immunoblots with individual data points are provided in Figs. S14 and S15. Statistical significance was determined by two-way ANOVA followed by Tukey’s multiple-comparison test. *p* < 0.05 was considered significant. Different letters (a-c) denote statistically significant differences between groups.

To further delineate the contribution of exosomal mA1AT in transferring tumorigenic traits to future metastatic sites, an *in vivo* exosome education model was employed. Non-tumor-bearing (Control) and 4T1 tumor-bearing (Tumor) BALB/c mice were systemically administered with TDEs either enriched in mA1AT (Exo^mA1AT+^) or selectively depleted of mA1AT (Exo^mA1AT-^). Gross phenotypic analyses of tumor-bearing groups, including tumor volume and weight, revealed a significant exacerbation of tumor growth in mice treated with Exo^mA1AT+^. Conversely, in tumor-bearing mice treated with Exo^mA1AT-^, a marked reduction in tumor burden was observed (Fig. 5, *A*–*E*).

Histological analysis of lung, liver and bone marrow sections revealed striking differences in immune cell infiltration patterns (Fig. 5, *F*–*H*). Tumor-bearing mice treated with mA1AT-enriched exosomes showed pronounced lymphocytic infiltration and disorganized tissue architecture. In contrast, tumor-bearing mice exposed to mA1AT-depleted exosomes showed notably reduced immune cell infiltration, with improved tissue morphology (Fig. 5, *F*–*H*). Remarkably, administration of mA1AT-enriched exosomes was sufficient to induce low to moderate lymphocytic infiltration in the lung, liver and bone marrow in non-tumor-bearing (control) mice, compared to control mice treated with mA1AT-depleted exosomes. Further, quantification of nucleus-to-cytoplasm (N:C) ratio and metastatic foci in the lung and liver tissue sections demonstrated that mA1AT-enriched exosome treatment significantly increased metastatic burden in distant normal tissues of tumor-bearing mice compared to untreated tumor-bearing mice or mice treated with mA1AT-depleted exosomes (Fig. S3, *A*–*D*). Since bone marrow cell alterations are a major indicator of irregularities, as assessed conventionally by biopsies, the smears were assessed for possible abnormalities. However, metastatic lesion quantification could not be performed since cell smears do not permit reliable histopathological assessment of metastatic foci. In addition, Exo^mA1AT+^ treatment upregulated pro-inflammatory mediators IL-6 and TNF-α, whereas Exo^mA1AT-^ treatment significantly suppressed their expression and concomitantly increased anti-inflammatory IL-4 and IL-10 in both control and tumor-bearing groups (Fig. 5, *I*–*K*). Individual data points displayed with their respective statistical quantifications, are shown in Figs. S14 and S15.

### TDE-borne mA1AT induces oncogenic traits and ER stress in control and tumor-bearing mice

**Figure 6:**
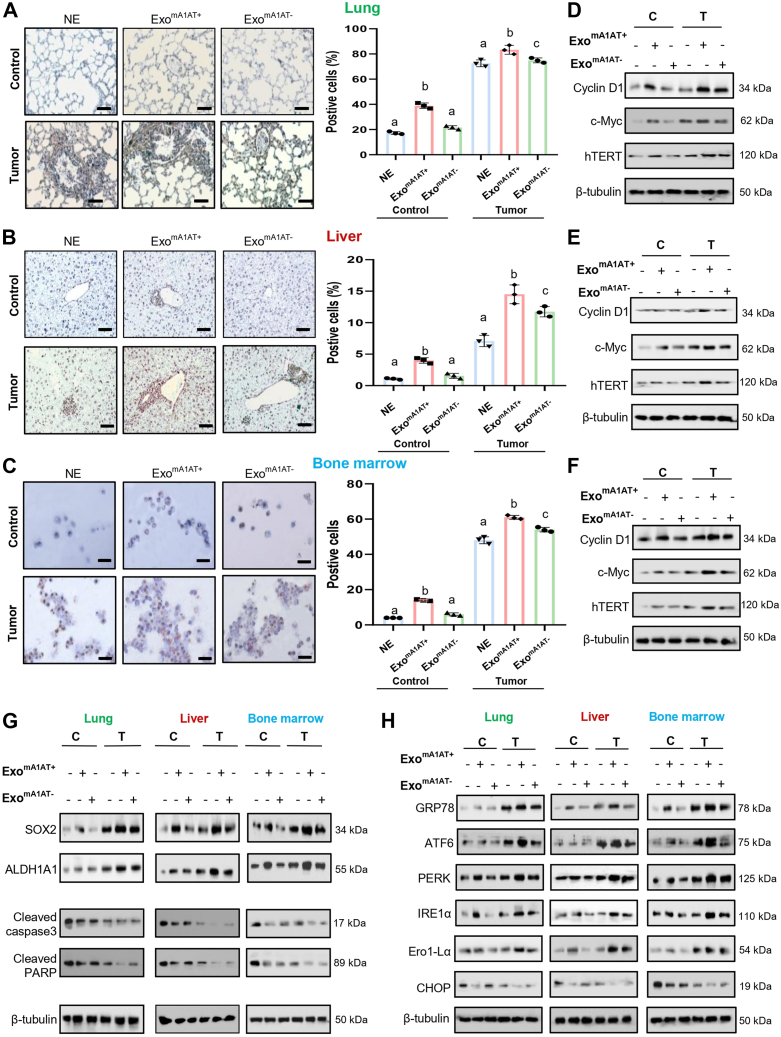
**TDE-borne mA1AT transmits tumorigenic traits to normal lung, liver and bone marrow cells *in vivo*.** Representative immunohistochemistry images and quantification of lung (*A*) and liver (*B*), and representative immunocytochemistry images and quantification of bone marrow cell smears (*C*), showing expression of Ki-67 in different experimental groups. Magnification: 20X, Scale bar: 50 μm. Western blot analyses of cyclin D1, c-Myc and hTERT in lung (*D*), liver (*E*) and bone marrow (*F*) tissues collected from control and tumor-bearing mice treated with NE, Exo^mA1AT+^ or Exo^mA1AT-^. Western blot analyses of lung, liver and bone marrow cells collected from control and tumor-bearing mice treated with NE, Exo^mA1AT+^ or Exo^mA1AT-^, assessing stemness and apoptotic markers (*G*) and UPR markers (*H*). All protein expressions were normalized against β-tubulin as the internal loading control. Data are presented as mean ± SD (*n* = biological replicates per group). Corresponding quantitative graphs of the immunoblots with individual data points are provided in Figs. S16–S22. Statistical significance was determined by two-way ANOVA followed by Tukey’s multiple-comparison test. *p* < 0.05 was considered significant. Different letters (a–c) denote statistically significant differences between groups.

Corollary to the effects of mA1AT in putative metastatic tissues, immunohistochemical analyses of lung and liver tissues, along with immunocytochemical analysis of bone marrow smears, demonstrated a significant increase in Ki-67 expression in mice treated with mA1AT-enriched exosomes, compared to those treated with either mA1AT-depleted exosomes or vehicle control (Fig. 6, *A*–*C*). Proliferation of cells was further confirmed by elevated expressions of cyclin D1, c-Myc and hTERT in lung, liver and bone marrow of mice receiving mA1AT-enriched exosomes (Fig. 6, *D*–*F*). In contrast, tissues from mice treated with mA1AT-depleted exosomes exhibited comparatively lower expression of the above proteins (Fig. 6, *D*–*F*). In addition, Exo^mA1AT+^ treatment in both control and tumor-bearing mice showed increased expressions of SOX2 and ALDH1A1, along with reduced expressions of cleaved caspase-3 and cleaved PARP (Fig. 6*G*). Furthermore, expression analyses revealed an increase in ER stress regulators, including GRP78, ATF6, PERK, IRE1α and ERO1-Lα, concomitant with downregulation of CHOP in lung, liver and bone marrow of Exo^mA1AT+^ -treated mice (Fig. 6*H*). Moreover, upregulated ER stress markers XBP-1s and ATF4 in lung, liver and bone marrow of Exo^mA1AT+^ -treated mice confirmed UPR activation (Fig. S2*K*). In contrast, Exo^mA1AT-^ or vehicle control groups showed significantly reduced ER stress signaling (Figs. 6*H* and S2*K*). Individual data points, along with their respective statistical quantifications, are shown in Figs. S16–S21, and S28.

### mA1AT leads to increased GRP78 expression in the cytosol

**Figure 7:**
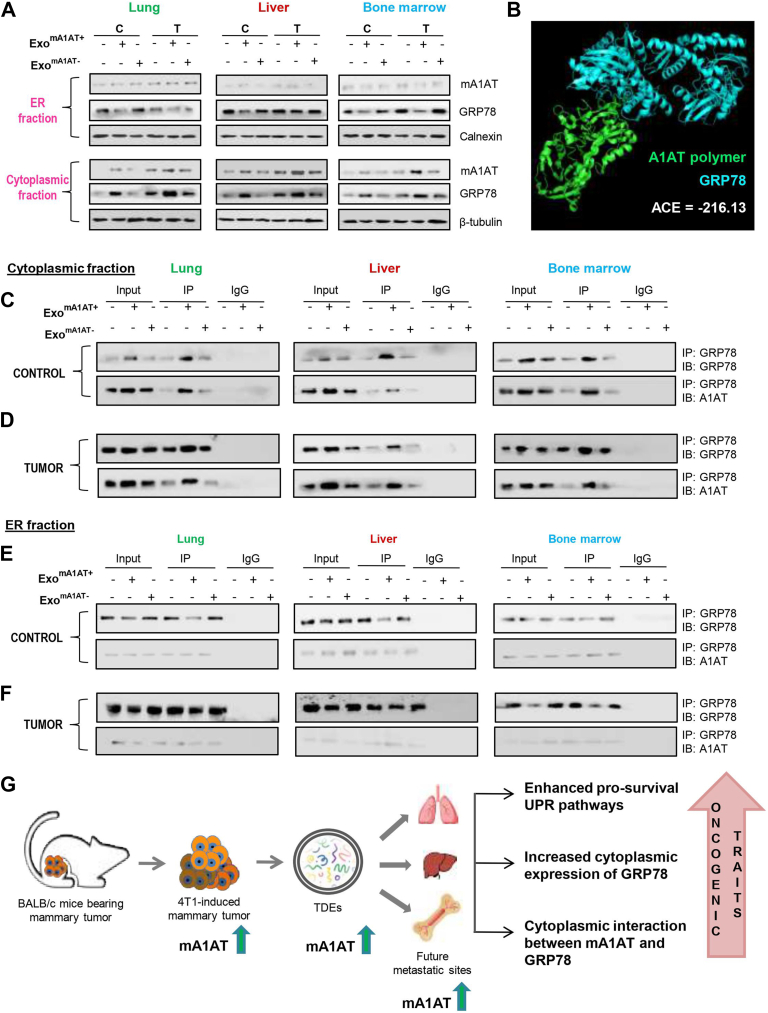
**TDE-borne mA1AT enhances cytoplasmic expression of GRP78 *in vivo***. *A*, subcellular fractionation and Western blot analyses of mA1AT and GRP78 in cytosolic and ER fractions of lung, liver and bone marrow tissues collected from control (C) and tumor-bearing (T) mice treated without or with Exo^mA1AT+^ or Exo^mA1AT-^. *B*, molecular docking model depicting spontaneous interaction A1AT polymer and GRP78. Co-immunoprecipitation experiment using either IgG control or anti-GRP78 antibody followed by Western blot in cytoplasmic fractions of lung, liver and bone marrow tissues collected from control mice (*C*) and tumor-bearing mice (*D*), treated without or with Exo^mA1AT+^ or Exo^mA1AT-^. Co-immunoprecipitation analyses in ER fractions of lung, liver and bone marrow tissues collected from control mice (*E*) and tumor-bearing mice (*F*), treated without or with Exo^mA1AT+^ or Exo^mA1AT-^. Cytosolic and ER protein expressions were normalized against β-tubulin and calnexin, respectively. Data are presented as mean ± SD (*n* = 3). Corresponding quantitative graphs with individual data points are provided in Figs. S23–S28. Statistical significance was determined by two-way ANOVA followed by Tukey’s multiple-comparison test. *p* < 0.05 was considered significant. Different letters (a–f) denote statistically significant differences between groups. *G*, Schematic model illustrating the proposed mechanism by which TDE-borne mA1AT induces tumorigenic traits in normal cells at future metastatic sites. Characteristic elevation of cytosolic GRP78, along with activation of pro-survival UPR, stemness, proliferative and inflammatory factors, and downregulation of apoptotic markers, induce acquisition of oncogenic traits in recipient healthy cells.

To determine how exogenous mA1AT influences GRP78 to activate UPR and increase oncogenic traits, subcellular fractionation followed by protein interactions were performed. Results revealed significant accumulation of mA1AT in the cytoplasmic compartments of lung, liver, and bone marrow from both control and tumor-bearing mice treated with mA1AT-enriched exosomes. This was accompanied by significant expression of GRP78 in the cytosol (Fig. 7*A*). In contrast, tissues from vehicle- or mA1AT-depleted exosome–treated mice exhibited minimal cytoplasmic mA1AT and predominant GRP78 expression in the ER (Fig. 7*A*).

Next, establishment of a functional association between mA1AT and GRP78 by *in silico* docking analysis indicated that GRP78 showed spontaneous binding affinity for A1AT polymer (ACE = −216.13; Fig. 7*B*) Furthermore, co-immunoprecipitation assays using cytoplasmic extracts from lung, liver and bone marrow confirmed the presence of A1AT in the GRP78-bound fraction, specifically in the mA1AT-enriched exosome-treated groups of both control and tumor-bearing mice (Fig. 7, *C* and *D*). The interaction was, however, absent in both vehicle-treated and mA1AT-depleted exosome-treated groups in the ER fraction of both control and tumor-bearing mice (Fig. 7, *E* and *F*). Individual data points, displayed with their respective statistical quantifications, are shown in Figs. S23–S28.

## Discussion

Emerging evidences suggest that extracellular vesicles, particularly exosomes, are critical mediators of intercellular communication in cancer, facilitating the transfer of oncogenic signals by inducing ER stress in neighboring cells (15). However, their effects on distant normal organs, which act as putative metastatic sites and accept disseminated tumor cells, remain moderately evaluated. Moreover, the specific molecular constituents within TDEs which may be responsible for such effects have not been well characterized. Amongst various TDE-borne molecules that can induce ER stress in recipient cells, misfolded proteins represent one of the least explored in the context of tumor progression, despite their well-established roles in the pathogenesis of other diseases (20), and limited evidence implicating them across different types of cancers (14). Cells often rely on UPR to maintain ER protein homeostasis (proteostasis) when challenged with elevated levels of misfolded proteins. In this study, mA1AT was identified as a unique cargo of exosomes secreted by 4T1 mammary tumor cells. Exosomes enriched in mA1AT significantly altered the phenotype of recipient lung, liver and bone marrow cells by inducing cytoplasmic expression of GRP78, activating pro-survival UPR, suppressing apoptotic signaling, increasing stemness, and promoting proliferation and inflammation. Depletion of mA1AT from these vesicles abolished their capacity to elicit these effects, thereby establishing the functional importance of mA1AT in driving TDE-mediated transfer of oncogenic traits to prospective metastatic sites.

Since both pro-survival UPR signaling and inflammation are known to drive cellular proliferation and enhance stemness (21), a mechanistic link between exosome-induced ER stress, increased stemness and enhanced cell growth may therefore be established. According to the hierarchical theory of CSCs, tumors originate from normal stem cells (NSCs) through processes involving genetic mutations or aberrant differentiation (22). Therefore, we presumed that during metastasis, NSCs residing in distant, healthy tissues may acquire CSC-like properties and function as tumor-initiating cells, leading to secondary tumor formation. Consequently, exposure to TDEs resulted in significant upregulation of cyclin D1, along with SOX2 and ALDH1A1, indicating enhanced proliferation and self-renewal of endogenous stem cells in healthy organs. In contrast, the non-stem cell population exhibited increased expression of cyclin D1 only, suggesting that TDEs primarily promote proliferative expansion in these differentiated cell populations. Taken together, these findings suggest that TDEs augment stemness potential of resident NSCs, simultaneously inducing proliferation in the non-stem cells. Since elevated stemness and proliferation are hallmarks of tumorigenic transformation, our results indicated that TDEs and their cargo derived from 4T1 mammary tumors can induce a pro-tumorigenic phenotype in distant, non-tumorigenic cells of future metastatic sites, through ER stress-mediated activation of survival, inflammatory, proliferation and stemness pathways.

Alterations, if any, in the exosomal cargo were initially assessed with tumor progression. Progressive tumor growth was confirmed by increased tumor volume and histopathological evidence of increased multinucleated cells (23). Exosomes isolated and characterized from these mice (24) indicated that the exosomal proteome became progressively enriched with misfolded proteins with the advancement of the tumor. It may therefore be presumed that the molecular composition of circulating TDEs reflects the proteostatic imbalance that occurs within the tumor and may eventually contribute to their pathological effects on neighboring healthy organs.

To authenticate the function of the bioactive constituents in TDEs, including misfolded proteins, the role of a “fertilizer” in Paget’s Seed and Soil theory of metastasis was interrogated (6). Accordingly, specific messages from the tumor which determine the eligibility of a putative distant organ to act as a harbor for disseminated cells, were assessed. The lung, liver and bone marrow are amongst the most common metastatic sites in breast cancer, making them critical targets for investigating systemic tumor influences (25). Consequently, our results indicated that TDE-bearing misfolded proteins not only induced proliferation, but also typically activated UPR in normal cells of future metastatic sites, a cellular stress pathway aimed at restoring ER homeostasis (14). GRP78, a key ER chaperone, acts as a master regulator of UPR by dissociating from the three principal UPR sensors, ATF6, IRE1α, and PERK, thereby initiating their activation. In normal cells, UPR activation initially promotes adaptive, pro-survival responses, including upregulation of chaperones, global attenuation of protein synthesis and activation of ER-associated degradation (ERAD) mechanisms (13). However, if ER stress persists and homeostasis is unattainable, the UPR shifts toward pro-apoptotic signaling, primarily mediated by CHOP (13). Our results indicate that internalization of TDEs led to robust induction of GRP78, along with upregulation of IRE1α, PERK and ATF6, and downregulation of CHOP. Concomitantly, reduced levels of cleaved caspase-3 and cleaved PARP suggested enhanced cellular survival and resistance to apoptosis in the TDE-treated cells. In addition to promoting survival, UPR is also known to trigger pro-inflammatory responses under certain conditions (26). Consistent with this fact, TDE exposure significantly upregulated pro-inflammatory cytokines IL-6 and TNF-α, while suppressing anti-inflammatory cytokines IL-10 and IL-4, in normal lung, liver and bone marrow cells, reiterating that UPR activation induce inflammatory responses in response to TDEs (26).

The search for the TDE-borne “fertilizer” culminated in the identification of a misfolded form of α-1 antitrypsin (mA1AT) consistently in mice tumors, TDEs from both mice and patients with breast cancer, and exosome-treated healthy primary lung, liver and bone marrow cells. Native A1AT is a glycoprotein and serine protease inhibitor, predominantly synthesized in the liver, but also expressed in lung, breast and other organs, from where it is secreted into the bloodstream (27), inhibits neutrophil elastase and protects tissues from proteolytic damage during inflammation (28). Recent studies suggest that A1AT may contribute to cancer progression by modulating immune response and protease activity (29), and blood A1AT levels may serve as an indicator of the efficacy of cancer treatment (30). Interestingly, misfolding and aggregation of A1AT are well-documented in diseases such as A1AT deficiency-related liver and lung disorders (31, 32), and is implicated in chronic ER stress in non-cancerous diseases (33).

To fortify the role of exosome-mediated transfer of mA1AT in interceding oncogenic traits, EDEM1, a well-characterized ER-resident lectin that facilitates proteasomal degradation of terminally misfolded glycoproteins (34), was overexpressed in 4T1 cells. EDEM1 overexpression led to a reduction in mA1AT levels in both tumor lysates and secreted exosomes. Treatment of primary lung, liver and bone marrow cells with either mA1AT-enriched (mA1AT^+^) or mA1AT-depleted (mA1AT^-^) exosomes indicated that cells exposed to exosomes enriched with mA1AT showed intracellular accumulation of mA1AT and initiated extreme alterations in healthy cells. In contrast, treatment with mA1AT-depleted exosomes failed to stimulate UPR, reduced inflammatory, stemness and proliferation markers, and increased pro-apoptotic markers, establishing TDE-borne mA1AT as a major contributor to the induction of pro-tumorigenic traits in normal tissues. Consistent with the *in vitro* findings, *in vivo* exosome education studies concurred that mice receiving mA1AT-enriched exosomes exhibited increased tumor burden. Probable metastatic sites such as the lung, liver and bone marrow showed elevated expression of pro-survival UPR markers, proliferation and stemness-associated markers and reduced levels of apoptotic markers, compared to mice treated with mA1AT-depleted exosomes. Furthermore, increased metastatic burden were observed in lung and liver of tumor-bearing mice receiving mA1AT-enriched exosomes. Tissues from healthy mice also demonstrated enhanced lymphocyte infiltration and increased expression of pro-inflammatory cytokines, indicating an exacerbated inflammatory response. In addition, significantly increased proliferation and stemness was observed in the mA1AT-enriched exosome-treated liver, lungs and bone marrow in both the healthy and tumor-bearing mice, substantiating the pathological role of mammary tumor-derived exosomal mA1AT in enhancing tumorigenic traits in non-malignant tissues. Although EDEM1 overexpression effectively reduced mA1AT abundance in both donor cells and exosomes, EDEM1 is known to function as a broader component of the ERAD machinery and may influence the degradation of additional misfolded proteins, which were not prominent in our investigation. Therefore, the conjecture that the observed phenotypic changes may also be attributed to additional misfolded proteins and not exclusively to mA1AT depletion, still exist. However, consistent attenuation of tumorigenic, pro-survival and inflammatory phenotypes in lung, liver and bone marrow cells following treatment with mA1AT-depleted exosomes, generated through EDEM1 overexpression in 4T1 cells, supports the notion that exosomal misfolded A1AT is a prominent and significant contributor to the molecular alterations induced at future metastatic sites.

Further awareness of the mechanism by which mA1AT activated UPR remained limited, barring the lone fact that GRP78 was significantly upregulated in exposed cells. GRP78, a key regulator of UPR, is predominantly localized within the ER (13). However, exogenous mA1AT was localized to the cytosol and its possible interaction with ER-bound GRP78 remained unexplored. Interestingly, emerging evidence suggests that under certain stress conditions, GRP78 may express in other cellular compartments, including the cytoplasm, where it may engage in non-canonical signaling to selectively activate the cytoplasmic domain of PERK and eventually initiate survival (35). In accordance, cells from both healthy and tumor-bearing mice treated with mA1AT-enriched exosomes revealed that the elevated GRP78 expression was specifically observed in the cytoplasmic fraction. Subsequently, potential interaction between GRP78 and mA1AT in the cytoplasm was predicted using *in silico* docking studies and validated by co-immunoprecipitation assays, which confirmed that cytoplasmic GRP78 associates with A1AT in the cytosol. Notably, increased cytoplasmic GRP78 was accompanied by activation of pro-survival UPR signaling branches, whereas mA1AT-depleted exosome treatment markedly reduced GRP78 redistribution and attenuated downstream pro-survival UPR activation. Together, these findings suggest a putative association between exosomal mA1AT accumulation, cytoplasmic redistribution of GRP78 and activation of non-canonical pro-survival UPR signaling (Fig. 7*G*), which in due course contributes to increased cellular proliferation and stemness, suppression of apoptosis and heightened inflammation at future metastatic sites. Depletion of mA1AT from exosomes abrogates the above effects, establishing its pivotal role in induction of pro-tumorigenic signaling at future metastatic sites, and establishing mA1AT as a specific target for therapeutic benefits in future.

A limitation of the present study is that all experiments were conducted using the murine 4T1 mammary carcinoma model. Although the 4T1 syngeneic model is widely used and recapitulates several key features of aggressive metastatic breast cancer, confirmation of these findings in additional experimental models, using multiple syngeneic cell lines, would further strengthen the conclusions. To minimize model-dependent bias, the biological effects of tumor-derived exosomes were evaluated in primary cultures of lung, liver and bone marrow cells isolated from independent mice, where consistent molecular and phenotypic alterations were observed across recipient cell populations. Future studies employing additional breast cancer cell lines, patient-derived models and clinical specimens will help extend these observations and further define the role of exosomal mA1AT in inducing tumorigenic effects at future metastatic sites.

## Experimental procedures

### Materials

Dulbecco’s Modified Eagle Medium (catalog no.: AT007A), fetal bovine serum (catalog no.: RM10951), and penicillin–streptomycin (catalog no.: A002A) were purchased from HiMedia Laboratories Private Limited, India. The B27 supplement (catalog no.: 17504044) and insulin (catalog no.: 41400045) were obtained from Thermo Fisher Scientific. For histological analysis, hematoxylin, eosin, cedarwood oil and D.P.X. mountant were procured from Merck (Germany) and paraplast high melt tissue embedding medium was obtained from Leica Biosystems (Netherlands). The Total Exosome Isolation Kit (catalog no.: 4478360) and exosome-depleted FBS (catalog no.: A2720801) were obtained from Thermo Fisher Scientific, USA. The protease inhibitor cocktail (catalog no.: P2714) and the collagenase–hyaluronidase cocktail (catalog no.: B20222) were purchased from Sigma-Aldrich. 8-Anilino-1-naphthalenesulfonic acid (ANS) dye (catalog no.: 10417-5G) and PKH67 (catalog no.: PKH67GL-1KT) dye were also purchased from Sigma-Aldrich. The Aldefluor assay kit for stem cell sorting (catalog no.: #01700) was procured from Stem Cell Technologies. Trypsin Gold (catalog no.: V5280), used for MALDI sample preparation, was purchased from Promega. EDEM1-specific primers were custom synthesized and obtained from Integrated DNA Technologies, and the pcDNA3.1(+) vector was a kind gift from the Cell Biology Laboratory, Department of Microbiology, University of Calcutta. T4 DNA ligase (catalog no.: M0202S), BamHI (catalog no.: R0136S) and EcoRI (catalog no.: R0101S) were obtained from New England BioLabs. Lipofectamine 2000 reagent (catalog no.: 11668019) was purchased from Invitrogen. Details of antibodies used in Western blot, immunohistochemistry and immunocytochemistry analyses are mentioned in Table 1. All other chemicals were of analytical grade and purchased from SRL, unless otherwise specified.

**Table 1:** List of primary and secondary antibodies used in Western blot and IHC

Name of antibodies	Company and Catalogue number	Dilutions	
		Western blot	IHC
Anti-CD63	Santa Cruz Biotechnology, USA; SC-5275, RRID: AB_627877	1:1000	-
Anti-CD81	Santa Cruz Biotechnology, USA; SC-166029, RRID: AB_2275892	1:1000	-
Anti-TST101	Santa Cruz Biotechnology, USA; SC-7964, RRID: AB_671392	1:1000	-
Anti-Albumin	Santa Cruz Biotechnology, USA; SC-271605; RRID: AB_10647230		
Anti-ALDH1A1	Santa Cruz Biotechnology, USA; SC-374149, RRID: AB_10917910	1:1000	-
Anti-SOX2	Santa Cruz Biotechnology, USA; SC-365823	1:1000	-
Anti-Cyclin D1	Santa Cruz Biotechnology, USA; SC-8396; RRID: AB_627344	1:1000	-
Anti-cMyc	Santa Cruz Biotechnology, USA; SC-47694; RRID: AB_627266	1:1000	-
Anti-hTERT	Santa Cruz Biotechnology, USA; SC-377511; RRID: AB_11150127	1:1000	-
Anti-IL-6	Santa Cruz Biotechnology, USA; SC-57315; RRID: AB_2127596	1:1000	-
Anti-TNF-α	Santa Cruz Biotechnology, USA; SC-12744	1:1000	-
Anti-IL-4	Santa Cruz Biotechnology, USA; SC-53084; RRID:AB_629791	1:1000	-
Anti-IL-10	Santa Cruz Biotechnology, USA; SC-365858; RRID:AB_10859554	1:1000	-
Anti-Cl-caspase3	Santa Cruz Biotechnology, USA; SC-22140; RRID:AB_2259586	1:1000	-
Anti-Cl-PARP	Santa Cruz Biotechnology, USA; SC-56196; RRID:AB_785085	1:1000	-
Anti-EDEM1	Santa Cruz Biotechnology, USA; SC-377394	1:1000	-
Anti-β-actin	Santa Cruz Biotechnology, USA; SC-47778	1:1000	-
Anti- β-tubulin	Santa Cruz Biotechnology, USA; SC-53140	1:1000	-
Anti-A1AT	Hycult Biotech, Netherlands; HM2358	1:1000	-
Anti-GRP78	Cell Signaling Technology, USA; 3177T; RRID:AB_2119845	1:1000	-
Anti-ATF6	Cell Signaling Technology, USA; 65880T; RRID:AB_2799696	1:1000	-
Anti-PERK	Cell Signaling Technology, USA; 3192T; RRID:AB_2095847	1:1000	-
Anti-IRE1α	Cell Signaling Technology, USA; 3294T; RRID:AB_823545	1:1000	-
Anti-ERO1-Lα	Cell Signaling Technology, USA; 3264T; RRID:AB_823684	1:1000	-
Anti-CHOP	Cell Signaling Technology, USA; 2895T; RRID: AB_2089254	1:1000	-
Anti-XBP-1s	Cell Signaling Technology, USA; 40435T; RRID: AB_2891025	1:1000	-
Anti-ATF4	Cell Signaling Technology, USA; 11815T; AB_2616025	1:1000	-
Anti-Ki67	Santa Cruz Biotechnology, USA; SC-23900; RRID:AB_627859	-	1:100
Goat Anti Mouse IgG-HRP	Santa Cruz Biotechnology, USA; SC-2005; RRID:AB_3717730	1:3000	1:200
Goat Anti Rabbit IgG-HRP	Santa Cruz Biotechnology, USA; SC-2004; RRID:AB_631746	1:3000	-

### Methodology

#### Cell culture

4T1 murine mammary cancer cells (RRID: CVCL_0125) were obtained from the National Centre for Cell Sciences, Pune, India. The cells were cultured in DMEM supplemented with 10% FBS and 1% penicillin-streptomycin. Cultures were maintained at 37 °C in a humidified atmosphere of 5% CO_2_. Cells in the log phase of growth were used for experiments to ensure optimal cell activity and reproducibility. Cells were tested for *mycoplasma* contamination at regular intervals (36).

#### Animal models

All animal handling and experimental protocols were conducted in accordance with the *Principles of Laboratory Animal Care* (NIH Publication No. 85–23, revised 1985) and approved by the Institutional Animal Ethical Committee (Registration Number 885/ac/05/CPCSEA). Additionally, all procedures complied with the Indian Laws of Animal Protection (ILAP) throughout the study. Female BALB/c mice (20 ± 2 g) were procured from the West Bengal Live Stock Development Corporation, Government of West Bengal, India. The animals were maintained under standard laboratory conditions, with controlled temperature (20 ± 2 °C), relative humidity (50 ± 10%) and a 12-h light/dark cycle. They were provided access to food and water ad libitum and acclimatized to laboratory conditions for 7 days prior to initiating experimental procedures (23).

#### Human patient sample

Blood from six (n = 6) triple-negative breast cancer patients and six (n = 6) age-matched healthy controls were obtained from Department of General Surgery, KPC MCH, Kolkata (KPCMCH/IEC/2025/322 dated 26.05. 2025), in accordance with the Institutional Review Board and Ethical Committee, following informed consent from patients. Serum exosomes were isolated as described later for subsequent analysis (36). All human studies reported in this manuscript abide by the ‘Declaration of Helsinki’ principles.

#### Tumor induction in mice

Mouse mammary 4T1 cells in their log phase of growth were orthotopically injected (1 × 10^4^ cells/ml in 50 μl PBS) into the inguinal fourth mammary fat pad of BALB/c mice (n = 3). Tumors were allowed to develop for 7, 14 and 21 days (T7, T14 and T21), respectively. For each group of tumor-bearing mice, a corresponding group of healthy mice was included as control (C7, C14 and C21), which received an equal volume of sterile PBS under identical conditions (36). After respective periods of tumor development, the mice were euthanized and blood samples were collected *via* thoracotomy and cardiac puncture for subsequent exosome isolation. Mammary gland, mammary tumors, lungs, liver and bone marrow were also harvested for subsequent analyses.

#### Tissue weight and volume measurement

The weights of the mammary fat pads from control mice and the tumors from tumor-bearing mice were determined using a digital precision balance. Tumor dimensions, including width and length, were measured using a digital Vernier caliper. Tumor volumes were calculated using the formula V = half × (W^2^ × L), where V, W, and L represent volume, width and length of the tumors, respectively (37).

#### Histopathological analysis

At the end of the experimental schedule, mice were euthanized, and mammary fat pad, mammary tumor, lung, liver, and femoral bone marrow samples were collected for histopathological and cytological analyses. Tissue specimens were fixed in Bouin’s solution, dehydrated through graded ethanol, embedded in paraffin, and sectioned at 5 μm thickness. Bone marrow smears were prepared directly on glass slides. Tissue sections and bone marrow smears were stained with hematoxylin and eosin (H&E) and examined under a light microscope (Olympus BX53; RRID: SCR_022568) at 40× magnification. Inflammatory cell infiltration was assessed in lung, liver and bone marrow samples using QuPath software (RRID: SCR_018257). For each animal, 5 to 10 representative non-overlapping microscopic fields of view (FOVs) were systematically selected from each section or smear, and infiltrating lymphocytes were quantified using QuPath annotation and cell-detection tools. Data were expressed as the mean number of lymphocytes per microscopic field (37).

For morphometric analysis, individual cellular and nuclear boundaries within H&E-stained lung and liver sections, were segmented using QuPath software (RRID: SCR_018257). A minimum of 100 cells per tissue section from each animal were analyzed. Quantitative morphometric parameters were exported and processed using the NumPy library (RRID: SCR_008633) in a Python environment (RRID: SCR_008394) to calculate the nucleus-to-cell (N:C) ratio for individual cells. Frequency distribution plots of N:C ratios were generated using Matplotlib (RRID: SCR_008624; 23).

Metastatic burden in lung and liver tissues was evaluated by identifying metastatic lesions based on the presence of discrete metastatic foci within the H&E-stained sections (38). Metastatic foci, a discrete cluster of neoplastic cells (>5 cohesive cells) exhibiting hyperchromatic nuclei and cytomorphological features distinct from the surrounding normal tissue, were counted across 5 to 10 non-overlapping FOVs per tissue section for each animal. The number of metastatic foci was expressed as mean ± SD per group (n = 3) and used as an indicator of metastatic seeding frequency and overall metastatic burden.

#### Exosome isolation from serum

Exosomes were isolated from blood of mice (n = 3) using the Total Exosome Isolation Kit (Thermo Fisher Scientific, Cat. No. 4478360), following the manufacturer’s instructions. Briefly, serum samples were centrifuged at 2000*g* for 30 min at 4 °C to remove cell debris. The supernatant was transferred to a fresh tube and the Exosome Precipitation Reagent was added at a ratio of 0.2 volumes relative to the total serum sample. The mixture was incubated at 4 °C for 30 min to facilitate precipitation. After incubation, the samples were centrifuged at 10,000*g* for 10 min and the pellet containing exosomes was resuspended in PBS for further analyses. The isolated exosomes were stored at −80 °C until further use.

#### Exosome size assessment by dynamic light scattering (DLS)

Dynamic light scattering (DLS) was used to measure the size distribution of exosomes in solution. The extracted exosomes were diluted five-fold in PBS, to ensure proper scattering intensity and avoid particle-particle interactions. Measurements were performed using a Zetasizer Nano ZS (Malvern Instruments Ltd) at room temperature, with parameters such as temperature, viscosity and refractive index set according to the instrument’s guidelines for biological samples. Each sample was analyzed in triplicate to ensure reproducibility and the average particle size was recorded (39).

#### Scanning electron microscopy

Isolated exosomes were resuspended in sterile PBS. A drop of the suspension was placed on a clean coverslip, spread evenly and air-dried. The dried samples (n = 3) were fixed with 3.7% glutaraldehyde for 2 h at room temperature in the dark. After fixation, the samples were briefly washed with PBS and dehydrated using ascending grades of ethanol. Following ethanol dehydration, the coverslips were immersed in chilled acetone for 10 min and air-dried overnight at room temperature. The prepared samples were coated with a thin layer of silver using a sputter coater and imaged using a scanning electron microscope (Zeiss 194 EVO-18-Special Edition, Germany; (40)).

#### Western blot analyses

Tissues, cells and exosomes were lysed in RIPA buffer (150 mM NaCl, 50 mM Tris, 0.1% Triton X-100 and 0.1% SDS, supplemented with a protease inhibitor cocktail) and centrifuged to isolate the protein. Equal amounts of protein (30 μg) were resolved by sodium dodecyl sulfate-polyacrylamide gel electrophoresis (SDS-PAGE) and transferred onto nitrocellulose membranes. The blots were then incubated overnight at 4 °C with the primary antibodies. After incubation with horseradish peroxidase (HRP)-conjugated secondary antibodies for 2 h at room temperature, bands were visualized using enhanced chemiluminescence (ECL) reagents and imaged with a ChemiDoc™ MP Imaging System (BioRad). Band intensities were quantified using the ImageJ software (NIH; RRID: SCR_003070) and normalized with β-tubulin (36).

#### Abundance of exosomal misfolded proteins by ANS fluorescent intensity

8-Anilino-1-naphthalenesulfonic acid (ANS), a fluorescent molecular probe that fluoresces upon binding to hydrophobic regions of proteins, was used to evaluate the abundance of misfolded proteins in exosomes. ANS stock solution was prepared in methanol and its concentration was determined using an extinction coefficient of 5000 M^−1^ cm^−1^ at 350 nm. Exosomal protein samples (2 μM) were incubated with a 15-fold molar excess of ANS in the dark at room temperature. Fluorescence measurements were conducted using an excitation wavelength of 420 nm to avoid inner filter effects and emission spectra were recorded from 450 nm to 550 nm at defined time intervals (n = 3). Fluorescence intensity values across the 450 to 550 nm range were plotted on the graph to assess the abundance of misfolded proteins (41).

#### Exosome uptake study by confocal imaging

Uptake of exosomes by primary lung, liver and bone marrow cells was assessed using PKH67-labeled exosomes (Sigma-Aldrich). Exosome labeling was performed following the manufacturer’s instructions. Briefly, exosomes were incubated with PKH67 dye in a diluent solution for 5 min at room temperature to allow dye incorporation into the exosome membrane. Excess dye was removed by ultracentrifugation. Primary cells were seeded in 6-well plates and incubated with the PKH67-labeled exosomes for 6 h at 37 °C. As a control, exosome-depleted supernatant from the final ultracentrifugation step was labeled with PKH67 and added to the primary cells. After incubation, cells were washed thoroughly with PBS, fixed with 4% paraformaldehyde and counterstained with DAPI to visualize the nuclei. Images were acquired using a laser-scanning confocal microscope (Olympus Fluoview FV1000) (FV-10 ASW 3.0 viewer image browser) to assess exosome uptake (42).

#### Primary cell culture and exosome treatment

Lungs were dissected under sterile conditions from healthy female BALB/c mice, minced and digested with collagenase-hyaluronidase cocktail (1 mg/ml) at 37 °C for 45 min. The digested tissue was triturated, filtered through a 70-μm cell strainer, centrifuged, and resuspended in complete DMEM with 10% fetal bovine serum, 2 mM L-glutamine, 1% penicillin-streptomycin and 0.1 μg/ml insulin. Cells were plated and cultured at 37 °C in a 5% CO_2_-95% air environment (43).

The liver, dissected from healthy female BALB/c mice, was perfused with perfusion buffer followed by collagenase IV digestion. The liver was excised, mechanically dispersed and filtered using a 70-μm strainer. Cells were cultured in DMEM supplemented with 10% fetal bovine serum, 2 mM L-glutamine, 1% penicillin-streptomycin and 0.1 μg/ml insulin and maintained at 37 °C (44).

Femurs were harvested from euthanized BALB/c mice under sterile conditions. The adjoining muscles and connective tissues were carefully removed, and both ends of the femurs were cut using sterile scissors. Bone marrow was flushed out with ice-cold PBS using a 1 ml syringe and passed through a 70 μm cell strainer to obtain a single cell suspension. The cells were cultured in complete DMEM medium supplemented with 10% fetal bovine serum, 1% penicillin-streptomycin, 2 mM L-glutamine and 0.1 μg/ml insulin. Cells were maintained at 37 °C in a humidified CO_2_ incubator (45).

All primary cultures were treated with exosomes isolated from either healthy mice (control) or from mammary tumor-bearing mice, from different progressive stages of tumor, *viz.* 7, 14, and 21 days. Exosomes were added to the culture medium at a concentration equivalent to 10 μg of exosomal protein per mL of medium. The cultures were incubated with exosomes for 24 h under standard conditions and thereafter processed for further analyses (42).

#### Stem- and non-stem cell sorting of lung, liver and bone marrow cells

Single cell suspensions from lung, liver and bone marrow tissues were sorted using the aldefluor assay reagent (Stem Cell Technologies; n = 3). Briefly, 1 × 10^6^/ml cells were resuspended in aldefluor assay buffer, containing the fluorescent ALDH substrate, bodipy-aminoacetaldehyde (BAAA) and incubated at 37 °C for 45 min. As a negative control, a portion of the cells were incubated with diethylaminobenzaldehyde (DEAB), a specific ALDH inhibitor. After incubation, cells were washed with assay buffer and resuspended in 500 μl of fresh buffer. Cells were first identified based on their light scatter properties (FSC-A vs. SSC-A) to exclude debris. Doublets were then excluded by gating on FSC-Height *versus* FSC-Width (FSC-H vs. FSC-W) and SSC-H *versus* SSC-W. Finally, the ALDH^+^ (stem) and ALDH^-^ (non-stem) populations were gated using the DEAB-treated control as a baseline. Sorting was performed using the FACS Aria III system (BD Biosciences). Sorted cells were collected in sterile tubes containing culture medium and subsequently used for different experiments (22).

#### Lung, liver, and bone marrow stem cell cultures

Sorted liver, lung and bone marrow stem cells were seeded at a density of 2.5 × 10^4^ cells per well in 6-well ultra-low attachment plates. The culture medium consisted of DMEM/F12 supplemented with 5 μg/ml bovine insulin, 20 ng/ml recombinant epidermal growth factor, B27 supplement and an antibiotic-antimycotic mix. Cultures were maintained at 37 °C in a humidified incubator (22).

#### Detection of misfolded proteins

Native polyacrylamide gel electrophoresis was performed to preserve the native conformation of proteins and also to detect misfolded proteins. An 8% polyacrylamide gel was prepared without denaturing agents, ensuring the maintenance of non-denatured protein structure. Protein samples were prepared using protein loading dye containing glycerol and bromophenol blue without adding β-mercaptoethanol and applying heat. Electrophoresis was carried out using a running buffer composed of Tris-Glycine without sodium dodecyl sulphate. Following electrophoresis, proteins were visualized by staining with Coomassie Brilliant Blue R-250 (46).

#### MALDI-ToF-MS and MS-MS analysis

Based on the separation of differentially expressed proteins using native PAGE, specific bands were selected depending on their expression with increasing days of tumorigenesis. The selected protein bands were excised under sterile conditions to minimize contamination. The gel fragments were washed and destained using ammonium bicarbonate/acetonitrile solution to remove the excess dye and prepare the protein for enzymatic digestion. Reduction and alkylation were performed by incubating the gel fragments in dithiothreitol (DTT), followed by iodoacetamide, to break disulfide bonds and prevent reoxidation, enhancing peptide digestion efficiency. Next, the treated gel fragments were subjected to in-gel trypsin digestion at 37 °C overnight, using mass spectrometry-grade trypsin (Trypsin Gold, Promega, Catalog Number – V5280). Peptides were extracted by adding trifluoroacetic acid and sonication, followed by desalting, to prepare the sample for analysis. The resulting peptide mixture was applied to a matrix-assisted laser desorption/ionization (MALDI) plate along with a matrix solution and peptide masses were determined using Bruker Autoflex Speed MALDI-TOF mass spectrometer (RRID: SCR_019757). To confirm the identification of the protein, selected peptide ions were subjected to tandem mass spectrometry (MS/MS). The peptide mass fingerprint and MS/MS data were analyzed against a protein sequence database for identification, using the Mascot software (RRID: SCR_014322; (47)).

#### Protease protection assay

To determine the topographical localization of mA1AT within extracellular vesicles, a protease protection assay was performed on representative exosomal preparations (C14, T14 and C-exo). Purified exosomes (∼20 μg total protein per reaction) were resuspended in PBS and subjected to three experimental conditions (untreated control, proteinase K treatment and combined proteinase K plus Triton X-100 treatment) to distinguish luminal cargo from externally associated proteins. Control samples were incubated in PBS under identical conditions without further treatment. For protease digestion, samples were incubated with proteinase K (100 ng/ml) at 37 °C for 30 min to degrade proteins exposed on the outer surface of intact vesicles. To assess membrane-protected localization, parallel samples were pretreated with 1% (v/v) Triton X-100 on ice for 20 min to disrupt vesicle integrity, followed by proteinase K digestion under identical conditions. Proteolytic reactions were terminated by the addition of 5 mM phenylmethylsulfonyl fluoride (PMSF) and immediate incubation on ice for 15 min. Samples were subsequently mixed with loading buffer and resolved by non-denaturing polyacrylamide gel electrophoresis to preserve conformational integrity, followed by immunoblot analysis using a polymer-specific A1AT antibody. Where indicated, parallel immunoblot analyses under denaturing conditions were performed to assess exosomal marker proteins and protease accessibility controls. All steps, except enzymatic digestion, were carried out at 4 °C or on ice to maintain vesicle integrity. Membrane integrity and protease activity were validated by monitoring the accessibility of internal (*e.g.*, TSG101) and external (*e.g.*, CD63) exosomal markers (48).

#### Construction of EDEM1-pcDNA3.1(+)

Total RNA was extracted from 4T1 cells using TRIzol. Reverse transcription was performed using Superscript III cDNA synthesis kit to generate cDNA (22). Forward (5′-CGCGGATCCATGCAATGGCGAGCGC-3′) and reverse (5′-CCCGAATTCCTGGACTGCAGGTTCTGATA-3′) primers were designed based on the complete cDNA sequence of the EDEM1 gene (NCBI Gene ID: 192193), incorporating BamHI and EcoRI restriction sites, respectively. The EDEM1 gene was amplified by PCR in a thermal cycler for 35 cycles and the product was confirmed by agarose gel electrophoresis. The PCR-amplified EDEM1 fragment and pcDNA3.1(+) expression vector were digested with BamHI and EcoRI, followed by ligation with T4 DNA ligase at 16 °C overnight. The ligated construct was transformed into *E. coli* DH5α competent cells and transformed colonies were grown on Luria-Bertani broth containing 100 μg/ml ampicillin. Colony PCR and restriction digestion analysis were performed to confirm the insertion of EDEM1 into the pcDNA3.1(+) vector (37).

#### Transfection of EDEM1-pcDNA plasmid

4T1 cells were seeded in 6-well plates and grown till 70% confluency. The recombinant EDEM1-pcDNA plasmid (2.5 μg) or empty pcDNA3.1(+) vector (2.5 μg) was transfected into cells using Lipofectamine 2000 reagent (5 μl per well) in serum-free medium. After 6 h, the medium was replaced with complete growth medium and the cells were incubated for 24 h. Transfection efficiency was confirmed by Western blot analysis for EDEM1 expression (37).

#### Exosome isolation from conditioned media of 4T1 cells

Exosomes were isolated from the conditioned media of 4T1 cells by ultracentrifugation, as described previously (49). Briefly, 4T1 cells were cultured in complete medium until they reached 70 to 80% confluency, after which the medium was replaced with exosome-depleted fetal bovine serum-containing medium. After 48 h of incubation, the cell viability was assessed using the trypan blue dye exclusion test and medium from cultures with >90% viability was used for isolation of exosomes. The conditioned media was centrifuged at 300*g* for 10 min, 2000*g* for 10 min and 10,000*g* for 30 min at 4 °C to remove cells, debris and apoptotic bodies, respectively. The supernatant was then filtered through a 0.22 μm filter to eliminate larger particles. Subsequently, the filtered media was subjected to ultracentrifugation at 100,000*g* for 70 min at 4 °C to precipitate the exosomes. The exosome pellet was washed with phosphate-buffered saline (PBS) and centrifuged again at 100,000*g* for 70 min. Finally, the exosomes were resuspended in PBS and stored at −80 °C for different experiments.

#### Nanoparticle Tracking Analysis (NTA)

Exosome-enriched preparations were diluted (1:1000) in 0.22 μm filtered PBS and analyzed using a NanoSight NS300 instrument (Malvern Panalytical) equipped with a 488 nm laser and NTA software version 3.4. Samples were introduced into the chamber using a syringe pump at a constant flow rate (speed 1500) and measured at room temperature. For each sample, three independent technical replicates of 1498 frames each were captured at 25 frames with a camera level of 8/9. Particle concentration were determined using a detection threshold of 5 and automatic blur size. Data are expressed as mean ± SEM of the three independent recordings (50).

#### *In vivo* exosome education studies

For the exosome-education studies, mammary tumors were induced in BALB/c mice (n = 5) as described previously and allowed to develop for 21 days. Mice were divided into 6 groups: Control (NE), Control mice which received misfolded A1AT-enriched exosomes (Exo^mA1AT+^), Control mice which received misfolded A1AT-depleted exosomes (Exo^mA1AT-^), Tumor-bearing mice (NE), Tumor-bearing mice which received misfolded A1AT-enriched exosomes (Exo^mA1AT+^) and Tumor-bearing mice which received misfolded A1AT-depleted exosomes (Exo^mA1AT-^), resuspended in 100 μg/100 μl PBS, *via* tail vein injection on alternate days for 3 weeks (51). On day 22, all mice were euthanized and tissues including lungs, liver and bone marrow, were collected for histopathological examination and Western blot analysis.

#### Isolation of cytoplasmic and ER fractions from tissue samples

Following the exosome-education study, cytoplasmic and ER fractions were isolated from the lung, liver and bone marrow tissues using differential ultracentrifugation. Briefly, tissues were homogenized in ice-cold sucrose homogenization buffer. The homogenates were centrifuged at 600*g* for 5 min at 4 °C to pellet nuclei and unbroken cells. The resulting supernatant was further centrifuged at 10,300*g* for 10 min at 4 °C to remove the mitochondria. The supernatants, containing ER and cytosolic components, were subjected to ultracentrifugation at 100,000*g* for 1 h at 4 °C in a fixed-angle rotor to separate the ER fraction (pellet) from the cytoplasmic fraction (supernatant). The ER pellet was washed twice in 1× PBS and resuspended in ice-cold PBS for further analyses (52).

#### *In silico* docking analysis

To validate the interaction between misfolded A1AT and GRP78, the crystal structures of the A1AT trimer and GRP78 were retrieved from the Protein Data Bank (https://www.rcsb.org). Protein-protein docking was subsequently performed using the HDOCK web server (RRID: SCR_024799; 51). The best docked complex was selected based on docking scores and predicted binding energy values. Following docking, the resulting complex was visualized using PyMOL molecular visualization software (RRID: SCR_000305) and images were generated for the configuration with the lowest binding energy (53).

#### Co-immunoprecipitation

Protein lysates of cytosolic, as well as ER fractions, derived from the all experimental groups of the exosome-education studies were incubated with anti-GRP78 antibody and 50 μl of agarose-protein A/G beads at 4 °C overnight to form immunocomplexes. The beads were washed three times with ice-cold RIPA buffer to remove unbound proteins. After washing, the immunocomplexes were eluted with sample buffer, heated for 10 min at 100 °C, resolved by 10% SDS-PAGE and transferred onto a nitrocellulose membrane. Co-immunoprecipitation of A1AT with GRP78 was analyzed using the anti-A1AT antibody (1:1000). The membranes were then incubated with peroxidase-conjugated secondary antibodies (1:3000) for 2 h at room temperature. Protein bands were visualized using ECL kit and imaged using the ChemiDoc™ MP Imaging System (BioRad). Band intensities were quantified with the ImageJ software and the interaction between GRP78 and A1AT was assessed by comparing relative band intensities across different samples. An IgG control was used to ensure that the observed interaction was specific and not due to non-specific binding of proteins to the beads (36).

#### Immunohistochemistry and immunocytochemistry

To assess cellular proliferation in mice subjected to exosome education, Ki-67 expression was evaluated in 5 μm paraffin-embedded sections of lung and liver tissues. Sections were deparaffinized, rehydrated and subjected to heat-induced epitope retrieval in citrate buffer (10 mM, pH 6.0). Endogenous peroxidase activity was blocked with 3% hydrogen peroxide, followed by blocking with serum. Sections were incubated overnight at 4 °C with anti-Ki-67 antibody, followed by HRP-conjugated secondary antibody and visualization using DAB substrate. Nuclei were counterstained with hematoxylin and stained sections were examined under a bright field microscope (Olympus BX53; 23).

For immunocytochemistry, air-dried bone marrow smears from the same experimental groups of mice were fixed in cold methanol, blocked and incubated with anti-Ki-67 antibody overnight at 4 °C. Detection was performed by incubating the slides successively with HRP-conjugated secondary antibody and DAB chromogen, followed by hematoxylin counterstaining. All images were acquired at 20X magnification, and quantification of Ki-67–positive cells was performed using QuPath software.

### Statistical analysis

All data in this study were analyzed using GraphPad Prism 5.0 software (GraphPad Software Inc; RRID: SCR_002798) and are presented as mean ± standard deviation (SD) from at least three independent experiments. For comparisons involving more than two groups, one-way analysis of variance (ANOVA) was utilized, while two-way ANOVA was employed for experiments involving two independent variables, followed by Tukey’s *post hoc* test for multiple comparisons. In all cases, *p*-values < 0.05 were considered to be statistically significant. Quantitative flow cytometry data were processed and analyzed using BD FACS Aria (RRID: SCR_018934) and the resulting cell populations were graphically represented.

## Data availability

The data generated in this study shall be made available upon request from the corresponding author.

## Supporting information

This article contains supporting information.

## Conflict of interest

The authors declare that they have no conflicts of interest with the contents of this article.
