# Supplementary material for: Exosomal misfolded α1-antitrypsin triggers a cytosolic GRP78-dependent unfolded protein response and pro-survival signaling in pre-metastatic tissues
Source: J Biol Chem. 2026 Jun 20;302(8):113282. doi: 10.1016/j.jbc.2026.113282 (PMC13393667; doi:10.1016/j.jbc.2026.113282)
Supplement: Supporting information [file mmc1.pdf]

Exosomal misfolded α1-antitrypsin triggers a cytosolic GRP78-dependent unfolded protein response and pro-survival signaling in pre-metastatic tissues

Supplementary Fig. S1

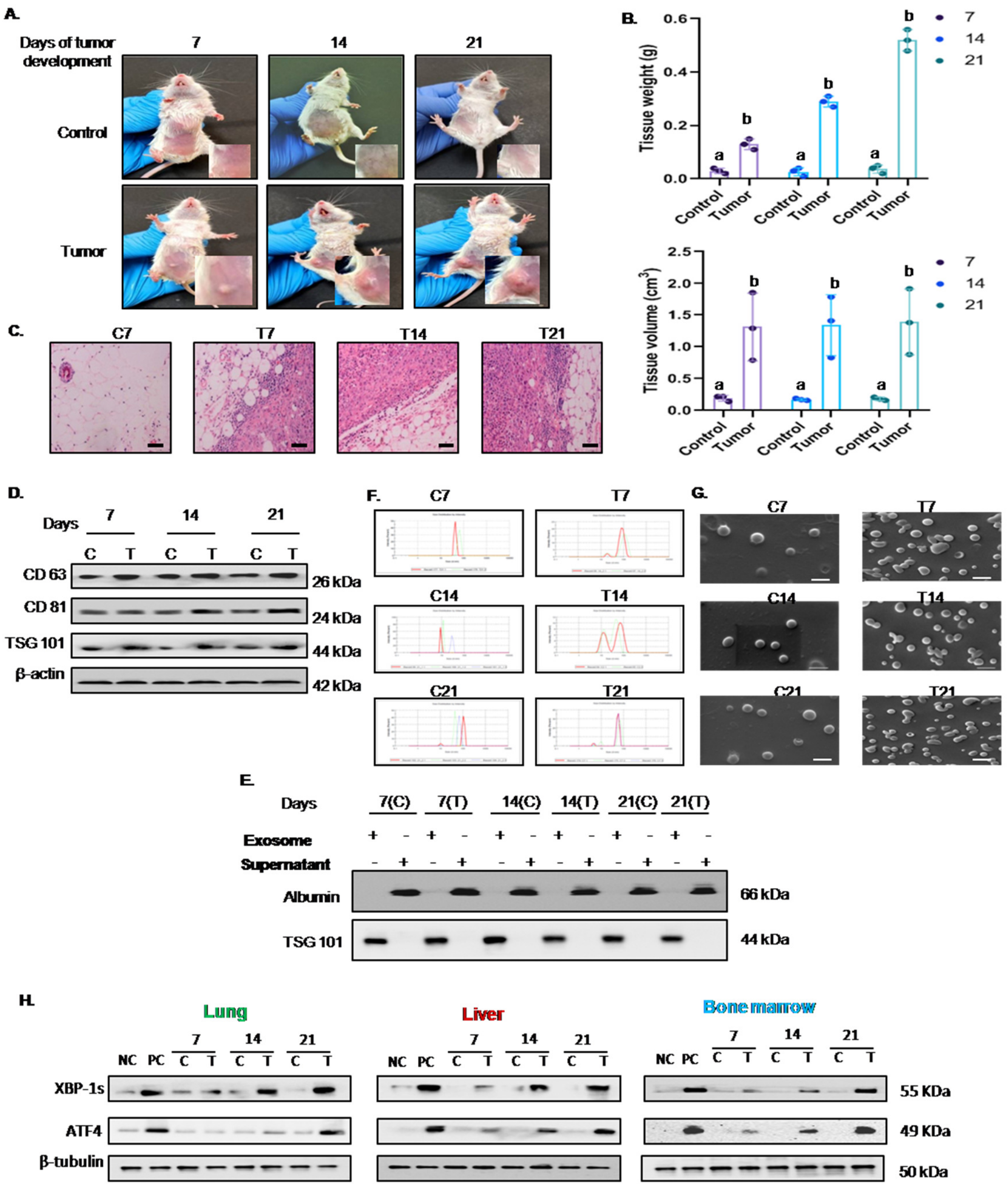

Fig S1. Characterization of TDEs from BALB/c mice at different stages of tumor progression and their effects on downstream UPR signalling. (A) Representative images of mammary tumors in BALB/c mice at 7,14 and21day post-inoculation with 4T1 cells, along with corresponding healthy control groups (n=3 biological replicates). (B) Quantification of mammary fat pad and tumor weight and volume, illustrating progressive tumor growth. (C) Hematoxylin and eosin-stained images of mammary fat pad and mammary tumor sections (Magnification: 20X; Scale bar: 50 μm; n=3 biological replicates) (D) Western blot analysis of exosomes isolated from the serum of control and tumor-bearing mice, confirming the presence of exosomal markers CD63, CD81 and TSG101. Protein expressions were normalized against β-tubulin as the internal loading control (n=3 biological replicates). (E) Western blot analysis of the exosomal marker TSG101 and the negative extracellular vesicle marker Albumin in exosomes isolated from the serum of control and tumor-bearing mice and in the corresponding exosome-depleted supernatant (n=3 biological replicates). (F) Size distribution of exosomes determined by DLS (n=3 biological replicates). (G) Morphological characterization of exosomes using SEM at 50,000X magnification, scale bar: 100 nm (n=3 biological replicates). (H) Western blot analyses of whole-cell lysates from lung, liver and bone marrow cells, treated with control exosomes (C) or TDEs (T), and probed for UPR markers (XBP-1s, ATF4; n=3 biological replicates). DTT-treated cells served as positive control (PC) and untreated healthy cells served as negative control (NC)**.** Protein expressions were normalized against β-tubulin as the internal loading control. Data are presented as mean ± SD (n = biological replicates per group). Rest of the corresponding quantitative graphs with individual data points are provided in Supplementary Fig. S29. Statistical significance was determined by two-way ANOVA followed by Tukey's multiple-comparison test. $P < 0.05$ was considered significant. Different letters (a–f) denote statistically significant differences between groups.

Table S1: Adjusted P values corresponding to Fig. S1B

	Tissue weight	Tissue volume
7 Day	0.0004	0.008
14 Day	<0.0001	0.0059
21 Day	<0.0001	0.0046

Supplementary Fig. S2

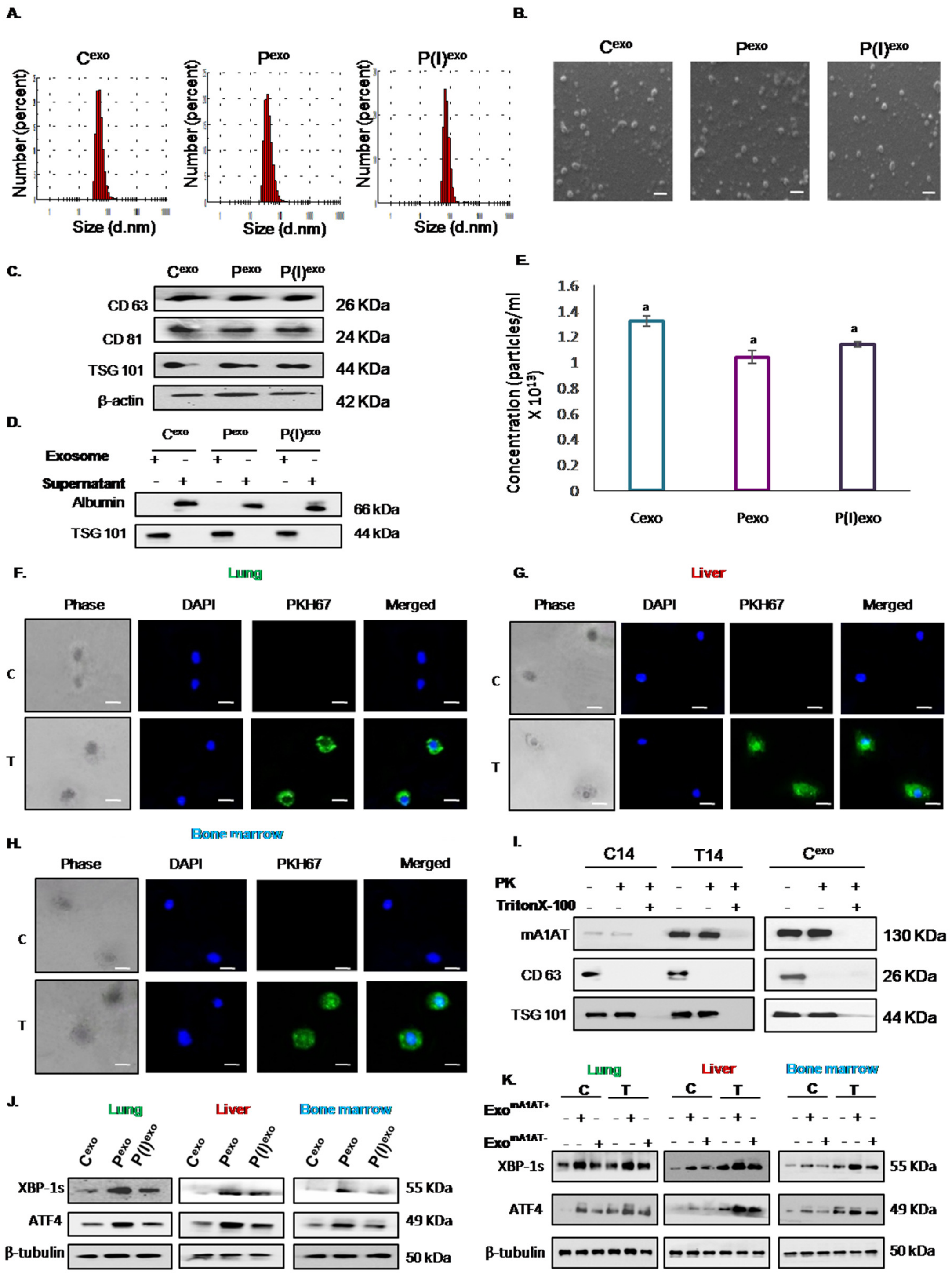

Fig S2. Characterization and uptake of mA1AT enriched or depleted exosomes, intraluminal localization of mA1AT and their effects on downstream UPR signaling. (A) Size distribution of exosomes isolated from the conditioned media of untransfected control 4T1cells (C^{exo}), 4T1 cells transfected with empty pcDNA3.1 vector (P^{exo}) and 4T1 cells transfected with pcDNA3.1 vector containing the EDEM1 gene ($P(I)^{exo}$) determined by DLS (n=3 biological replicates). (B) Morphological characterization of exosomes using SEM at 50,000X magnification, scale bar: 100 nm (n=3 biological replicates). (C) Western blot analysis of exosomes isolated from different experimental groups (C^{exo},P^{exo},$P(I)^{exo}$) confirming the presence of exosomal markers CD63, CD81 and TSG101 (n=3 biological replicates). (D) Western blot analysis of the exosomal marker TSG101 and the negative extracellular vesicle marker Albumin in exosomes isolated from different experimental groups (C^{exo},P^{exo},$P(I)^{exo}$) and in the corresponding exosome-depleted supernatant (n=3 biological replicates). (E) Particle concentration of exosomes isolated from different experimental groups (C^{exo},P^{exo},$P(I)^{exo}$) measured by Nanoparticle Tracking Analysis (NTA). (F–H) Respective confocal microscopic images of primary lung (F), liver (G) and bone marrow (H) control (C) and treated (T) cells showing uptake of PKH67-labeled exosomes (green); nuclei are counterstained with DAPI (blue). Exosome-depleted supernatant (PKH67-labeled) was used as control. Magnification, 40X; scale bar, 20 μm. (I) Western blot analysis of mA1AT, CD63 and TSG101 in representative exosome samples (C14, T14 and C-exo) subjected to untreated control, proteinase K (PK) treatment and PK in the presence of Triton X-100. Samples were analyzed following native/SDS polyacrylamide gel electrophoresis for mA1AT/CD 63 and TSG101 respectively. Data are representative of three independent biological replicates (n=3 biological replicates). (J-K) Western blot analysis assessing UPR markers (XBP-1s, ATF4), of (J) lung, liver and bone marrow cells treated with mA1AT–enriched or –depleted exosomes and (K) lung, liver and bone marrow cells collected from control and tumor-bearing mice treated with NE, Exo^{mA1AT+} or Exo^{mA1AT-}. Protein expressions were normalized against β-tubulin as the internal loading control. Data are presented as mean ± SD (n = 3 biological replicates per group). Corresponding quantitative graphs with individual data points are provided in Supplementary Figs. S30-S31. Statistical significance was determined by two-way ANOVA followed by Tukey's multiple-comparison test. $P < 0.05$ was considered significant. Different letters (a–f) denote statistically significant differences between groups.

Table S2: Adjusted P values corresponding to Fig. S2E

	Adjusted P value
C^{exo} vs. P^{exo}	0.0763
C^{exo} vs. $P(I)^{exo}$	0.0628
P^{exo} vs. $P(I)^{exo}$	0.051

Supplementary Fig. S3

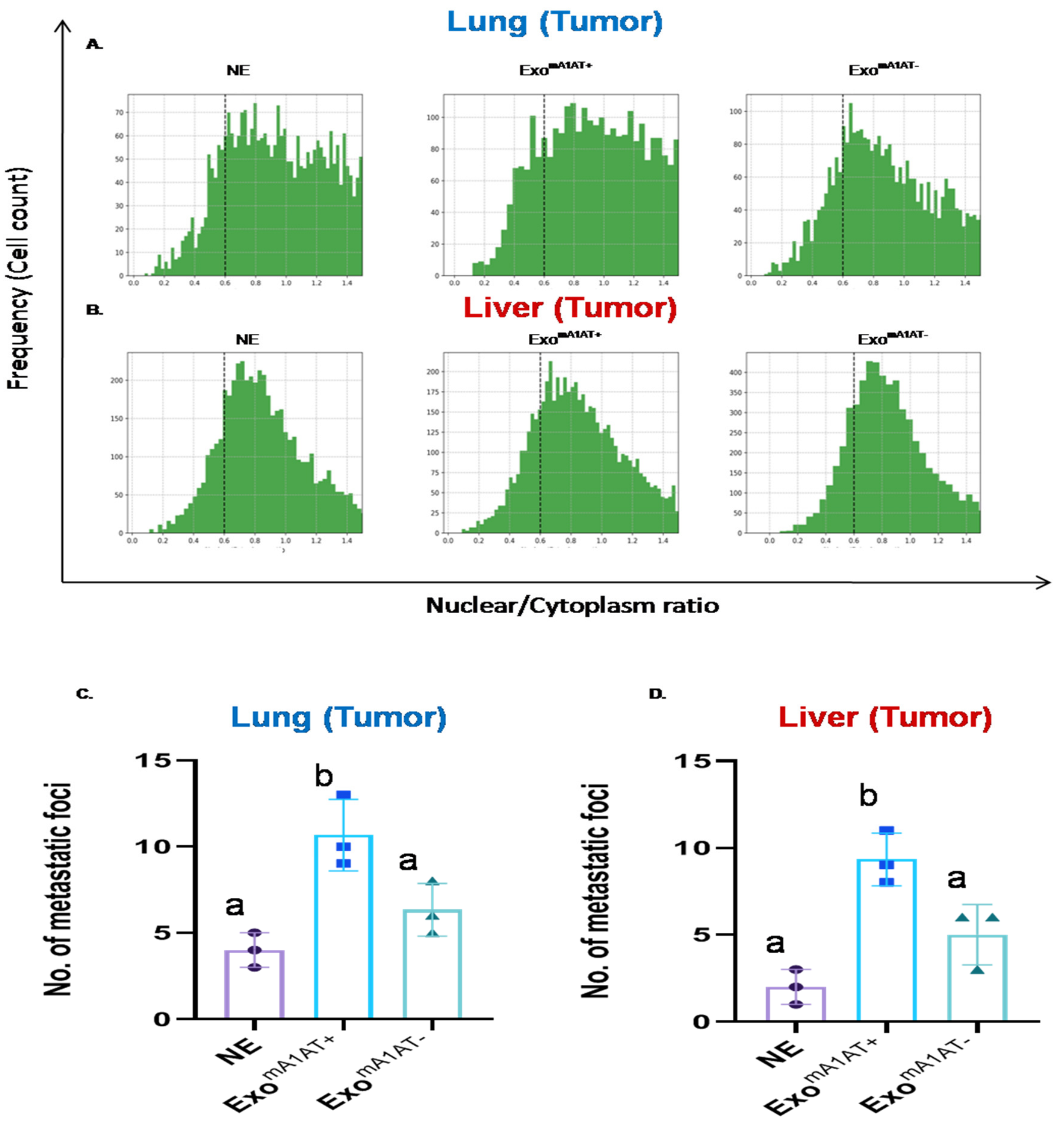

Fig S3. mA1AT-enriched exosome treatment increases metastatic burden in the lung and liver of tumor-bearing mice. (A, B) Quantification of nuclear-to-cytoplasmic ratio from HE-stained sections of (A) lung and (B) liver tissues isolated from tumor-bearing mice treated with NE, Exo^{mA1AT+} or Exo^{mA1AT-}. Histomorphometric analysis was performed to assess changes associated with metastatic progression. (C, D) Quantification of metastatic foci in HE-stained sections of (C) lung and (D) liver tissues from tumor-bearing mice treated with NE, Exo^{mA1AT+} or Exo^{mA1AT-}. Data are presented as mean ± SD (n=3 biological replicates). Statistical significance was determined using two-way ANOVA followed by Tukey's multiple-comparison test. $P < 0.05$ was considered statistically significant. Different lowercase letters (a–b) indicate statistically significant differences among groups.

Table S3: Adjusted P values corresponding to Fig. S3C-D

	Lung	Liver
NE vs. T + Exo^{mA1AT+}	0.0053	0.002
NE vs. T + Exo^{mA1AT-}	0.2516	0.0984
T + Exo^{mA1AT+} vs. T + Exo^{mA1AT-}	0.0368	0.0248

Supplementary Fig. S4

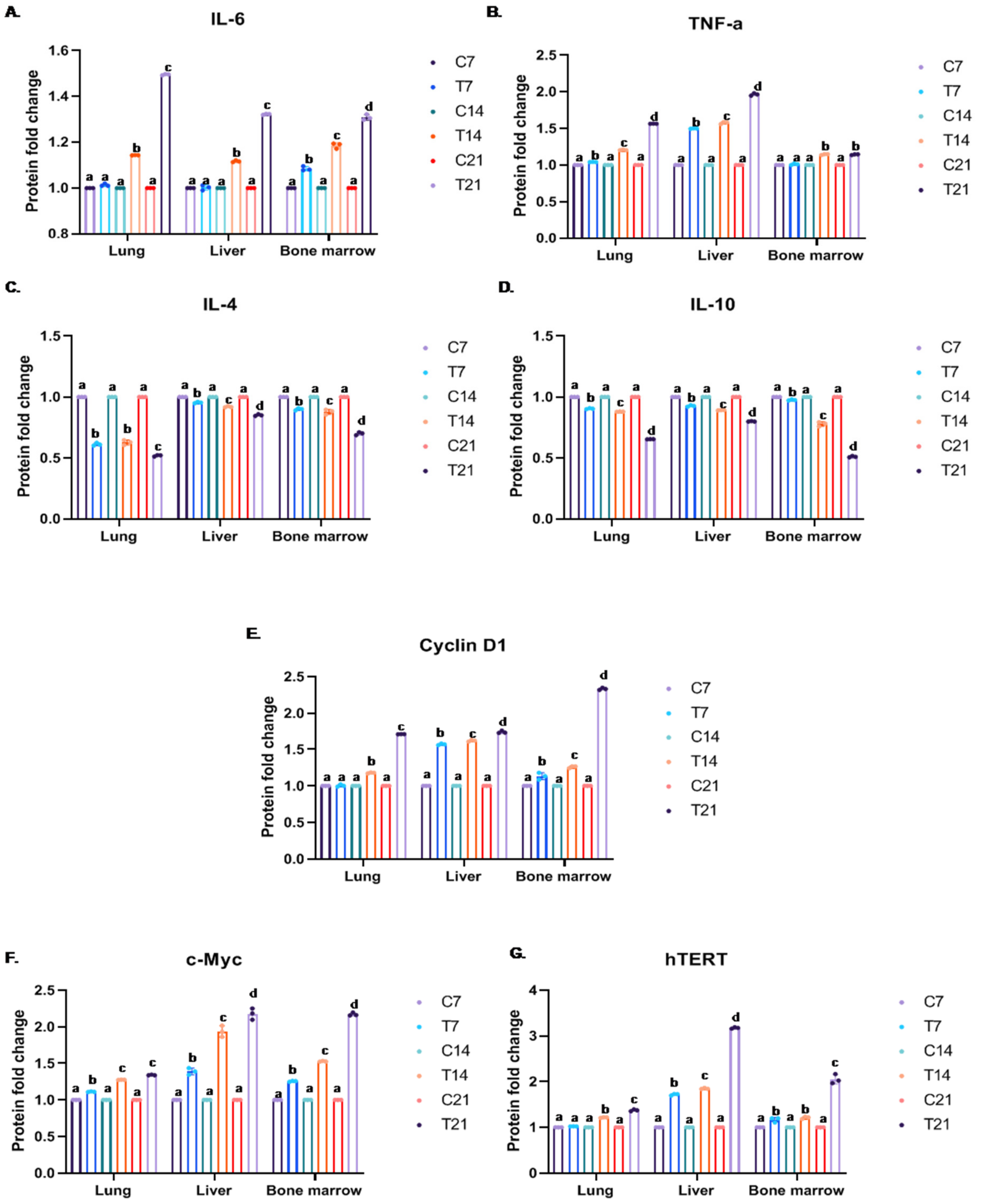

Fig S4. Densitometric quantification of inflammation- and proliferation-associated markers shown in Fig. 1E and 1F. (A–D) Quantitative densitometric analyses of (A) IL-6, (B) TNF-α, (C) IL-4 and (D) IL-10 expression shown in Fig. 1E. (E–G) Densitometric analyses of (E) Cyclin D1, (F) c-Myc and (G) hTERT expression shown in Fig. 1F. Data are presented as mean

± SD (n=3 biological replicates). Statistical significance was determined using two-way ANOVA followed by Tukey's multiple-comparison test. P < 0.05 was considered statistically significant. Lowercase letters (a–d) indicate statistically significant differences among groups.

Table S4A: Adjusted P values corresponding to Fig. S4A-D

	IL-6			TNF-α			IL-4			IL-10		
	Lung	Liver	BM	Lung	Liver	BM	Lung	Liver	BM	Lung	Liver	BM
C7 vs. T7	0.0807	0.9993	<0.0001	<0.0001	<0.0001	0.3082	<0.0001	<0.0001	<0.0001	<0.0001	<0.0001	<0.0001
C7 vs. C14	>0.9999	>0.9999	>0.9999	>0.9999	>0.9999	>0.9999	>0.9999	>0.9999	>0.9999	>0.9999	>0.9999	>0.9999
C7 vs. T14	<0.0001	<0.0001	<0.0001	<0.0001	<0.0001	<0.0001	<0.0001	<0.0001	<0.0001	<0.0001	<0.0001	<0.0001
C7 vs. C21	>0.9999	>0.9999	>0.9999	>0.9999	>0.9999	>0.9999	>0.9999	>0.9999	>0.9999	>0.9999	>0.9999	>0.9999
C7 vs. T21	<0.0001	<0.0001	<0.0001	<0.0001	<0.0001	<0.0001	<0.0001	<0.0001	<0.0001	<0.0001	<0.0001	<0.0001
T7 vs. C14	0.0807	0.9993	<0.0001	<0.0001	<0.0001	0.3082	<0.0001	<0.0001	<0.0001	<0.0001	<0.0001	<0.0001
T7 vs. T14	<0.0001	<0.0001	<0.0001	<0.0001	<0.0001	<0.0001	0.3275	0.0003	0.0462	<0.0001	<0.0001	<0.0001
T7 vs. C21	0.0807	0.9993	<0.0001	<0.0001	<0.0001	0.3082	<0.0001	<0.0001	<0.0001	<0.0001	<0.0001	<0.0001
T7 vs. T21	<0.0001	<0.0001	<0.0001	<0.0001	<0.0001	<0.0001	<0.0001	<0.0001	<0.0001	<0.0001	<0.0001	<0.0001
C14 vs. T14	<0.0001	<0.0001	<0.0001	<0.0001	<0.0001	<0.0001	<0.0001	<0.0001	<0.0001	<0.0001	<0.0001	<0.0001
C14 vs. C21	>0.9999	>0.9999	>0.9999	>0.9999	>0.9999	>0.9999	>0.9999	>0.9999	>0.9999	>0.9999	>0.9999	>0.9999
C14 vs. T21	<0.0001	<0.0001	<0.0001	<0.0001	<0.0001	<0.0001	<0.0001	<0.0001	<0.0001	<0.0001	<0.0001	<0.0001
T14 vs. C21	<0.0001	<0.0001	<0.0001	<0.0001	<0.0001	<0.0001	<0.0001	<0.0001	<0.0001	<0.0001	<0.0001	<0.0001
T14 vs. T21	<0.0001	<0.0001	<0.0001	<0.0001	<0.0001	0.9995	<0.0001	<0.0001	<0.0001	<0.0001	<0.0001	<0.0001
C21 vs. T21	<0.0001	<0.0001	<0.0001	<0.0001	<0.0001	<0.0001	<0.0001	<0.0001	<0.0001	<0.0001	<0.0001	<0.0001

Table S4B: Adjusted P values corresponding to Fig. S4E-G

Fig, S4 (E-G)	Cyclin D1			c-Myc			hTERT		
	Lung	Liver	BM	Lung	Liver	BM	Lung	Liver	BM
C7 vs. T7	0.9989	<0.0001	<0.0001	0.0003	<0.0001	<0.0001	0.9353	<0.0001	<0.0001
C7 vs. C14	>0.9999	>0.9999	>0.9999	>0.9999	>0.9999	>0.9999	>0.9999	>0.9999	>0.9999
C7 vs. T14	<0.0001	<0.0001	<0.0001	<0.0001	<0.0001	<0.0001	<0.0001	<0.0001	<0.0001
C7 vs. C21	>0.9999	>0.9999	>0.9999	>0.9999	>0.9999	>0.9999	>0.9999	>0.9999	>0.9999
C7 vs. T21	<0.0001	<0.0001	<0.0001	<0.0001	<0.0001	<0.0001	<0.0001	<0.0001	<0.0001
T7 vs. C14	0.9989	<0.0001	<0.0001	0.0003	<0.0001	<0.0001	0.9353	<0.0001	<0.0001
T7 vs. T14	<0.0001	0.0011	<0.0001	<0.0001	<0.0001	<0.0001	<0.0001	<0.0001	0.4589
T7 vs. C21	0.9989	<0.0001	<0.0001	0.0003	<0.0001	<0.0001	0.9353	<0.0001	<0.0001
T7 vs. T21	<0.0001	<0.0001	<0.0001	<0.0001	<0.0001	<0.0001	<0.0001	<0.0001	<0.0001
C14 vs. T14	<0.0001	<0.0001	<0.0001	<0.0001	<0.0001	<0.0001	<0.0001	<0.0001	<0.0001
C14 vs. C21	>0.9999	>0.9999	>0.9999	>0.9999	>0.9999	>0.9999	>0.9999	>0.9999	>0.9999
C14 vs. T21	<0.0001	<0.0001	<0.0001	<0.0001	<0.0001	<0.0001	<0.0001	<0.0001	<0.0001
T14 vs. C21	<0.0001	<0.0001	<0.0001	<0.0001	<0.0001	<0.0001	<0.0001	<0.0001	<0.0001
T14 vs. T21	<0.0001	<0.0001	<0.0001	0.0835	<0.0001	<0.0001	<0.0001	<0.0001	<0.0001
C21 vs. T21	<0.0001	<0.0001	<0.0001	<0.0001	<0.0001	<0.0001	<0.0001	<0.0001	<0.0001

Supplementary Fig. S5

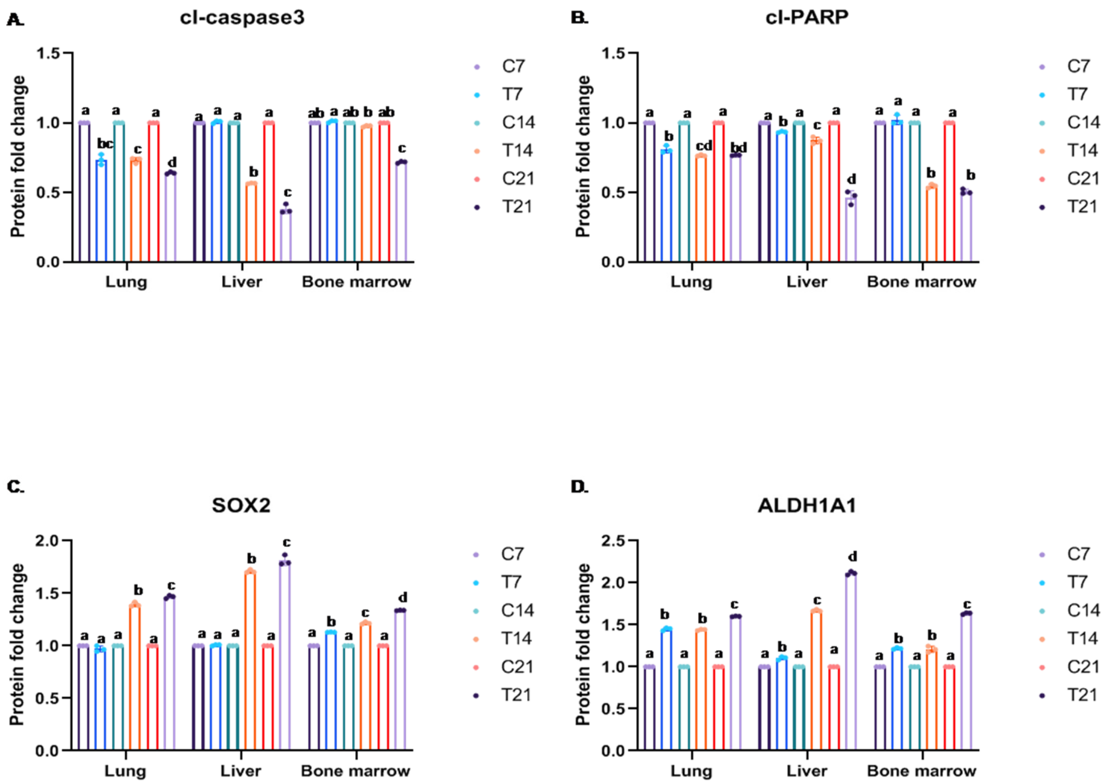

Fig S5. Densitometric quantification of apoptosis- and stemness-associated markers shown in Fig. 1G and 1H. (A–B) Quantitative densitometric analyses of (A) Cl-caspase3 and (B) Cl-PARP expression shown in Fig. 1G. (C–D) Densitometric analyses of (C) SOX2 and (D) ALDH1A1 expression shown in Fig. 1H. Data are presented as mean ± SD (n=3 biological replicates). Statistical significance was determined using two-way ANOVA followed by Tukey's multiple-comparison test. $P < 0.05$ was considered statistically significant. Lowercase letters (a–d) indicate statistically significant differences among groups.

Table S5: Adjusted P values corresponding to Fig. S5A-D

	cl-caspase3			cl-PARP			SOX2			ALDH1A1		
	Lung	Liver	BM	Lung	Liver	BM	Lung	Liver	BM	Lung	Liver	BM
C7 vs. T7	<0.0001	0.988	0.9374	<0.0001	0.0006	0.6597	0.1973	0.9924	<0.0001	<0.0001	<0.0001	<0.0001
C7 vs. C14	>0.9999	>0.9999	>0.9999	>0.9999	>0.9999	>0.9999	>0.9999	>0.9999	>0.9999	>0.9999	>0.9999	>0.9999
C7 vs. T14	<0.0001	<0.0001	0.2768	<0.0001	<0.0001	<0.0001	<0.0001	<0.0001	<0.0001	<0.0001	<0.0001	<0.0001
C7 vs. C21	>0.9999	>0.9999	>0.9999	>0.9999	>0.9999	>0.9999	>0.9999	>0.9999	>0.9999	>0.9999	>0.9999	>0.9999
C7 vs. T21	<0.0001	<0.0001	<0.0001	<0.0001	<0.0001	<0.0001	<0.0001	<0.0001	<0.0001	<0.0001	<0.0001	<0.0001
T7 vs. C14	<0.0001	0.988	0.9374	<0.0001	0.0006	0.6597	0.1973	0.9924	<0.0001	<0.0001	<0.0001	<0.0001
T7 vs. T14	0.9984	<0.0001	0.0408	0.0332	0.0013	<0.0001	<0.0001	<0.0001	<0.0001	0.9525	<0.0001	0.9242
T7 vs. C21	<0.0001	0.988	0.9374	<0.0001	0.0006	0.6597	0.1973	0.9924	<0.0001	<0.0001	<0.0001	<0.0001
T7 vs. T21	<0.0001	<0.0001	<0.0001	0.0541	<0.0001	<0.0001	<0.0001	<0.0001	<0.0001	<0.0001	<0.0001	<0.0001
C14 vs. T14	<0.0001	<0.0001	0.2768	<0.0001	<0.0001	<0.0001	<0.0001	<0.0001	<0.0001	<0.0001	<0.0001	<0.0001
C14 vs. C21	>0.9999	>0.9999	>0.9999	>0.9999	>0.9999	>0.9999	>0.9999	>0.9999	>0.9999	>0.9999	>0.9999	>0.9999
C14 vs. T21	<0.0001	<0.0001	<0.0001	<0.0001	<0.0001	<0.0001	<0.0001	<0.0001	<0.0001	<0.0001	<0.0001	<0.0001
T14 vs. C21	<0.0001	<0.0001	0.2768	<0.0001	<0.0001	<0.0001	<0.0001	<0.0001	<0.0001	<0.0001	<0.0001	<0.0001
T14 vs. T21	<0.0001	<0.0001	<0.0001	>0.9999	<0.0001	0.059	<0.0001	<0.0001	<0.0001	<0.0001	<0.0001	<0.0001
C21 vs. T21	<0.0001	<0.0001	<0.0001	<0.0001	<0.0001	<0.0001	<0.0001	<0.0001	<0.0001	<0.0001	<0.0001	<0.0001

Supplementary Fig. S6

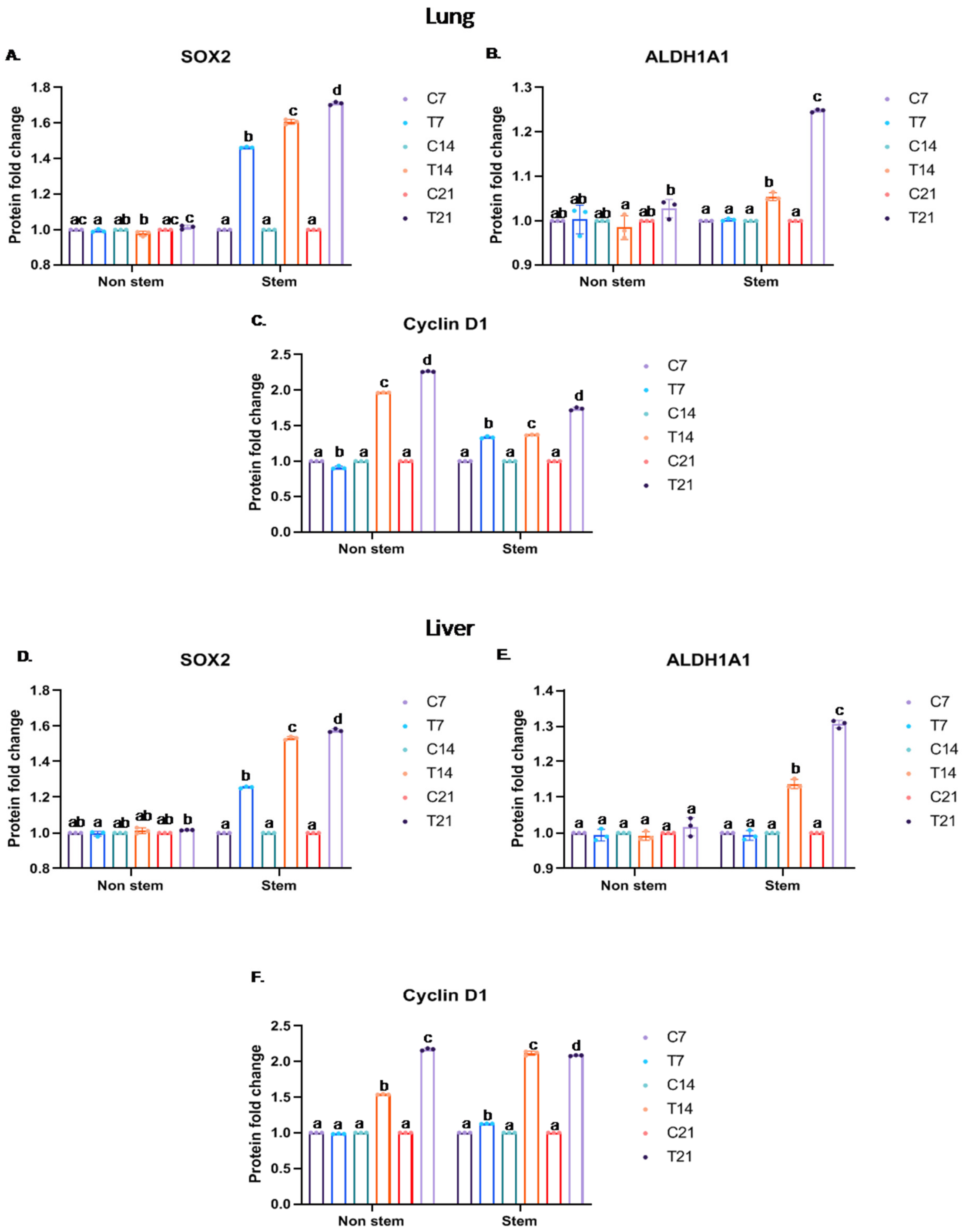

Fig S6. Densitometric quantification of stemness- and proliferation-associated markers shown in Fig. 2D and 2H. (A–C) Densitometric analyses of (A) SOX2, (B) ALDH1A1 and (C) Cyclin D1 expression in lung tissue shown in Fig. 2D. (D-F) Densitometric analyses of (D)

SOX2, (E) ALDH1A1 and (F) Cyclin D1 expression in liver tissue shown in Fig. 2H. Data are presented as mean ± SD (n=3 biological replicates). Statistical significance was determined using two-way ANOVA followed by Tukey's multiple-comparison test. $P < 0.05$ was considered statistically significant. Lowercase letters (a–d) indicate statistically significant differences among groups.

Table S6: Adjusted P values corresponding to Fig. S6A-F

	SOX2		ALDH1A1		Cyclin D1	
Lung	Non-stem	Stem	Non-stem	Stem	Non-stem	Stem
C7 vs. T7	0.8766	<0.0001	0.9999	>0.9999	<0.0001	<0.0001
C7 vs. C14	>0.9999	>0.9999	>0.9999	>0.9999	>0.9999	>0.9999
C7 vs. T14	0.0092	<0.0001	0.7508	0.0008	<0.0001	<0.0001
C7 vs. C21	>0.9999	>0.9999	>0.9999	>0.9999	>0.9999	>0.9999
C7 vs. T21	0.2459	<0.0001	0.1967	<0.0001	<0.0001	<0.0001
T7 vs. C14	0.8766	<0.0001	0.9999	>0.9999	<0.0001	<0.0001
T7 vs. T14	0.1051	<0.0001	0.6069	0.0014	<0.0001	0.0035
T7 vs. C21	0.8766	<0.0001	0.9999	>0.9999	<0.0001	<0.0001
T7 vs. T21	0.0273	<0.0001	0.294	<0.0001	<0.0001	<0.0001
C14 vs. T14	0.0092	<0.0001	0.7508	0.0008	<0.0001	<0.0001
C14 vs. C21	>0.9999	>0.9999	>0.9999	>0.9999	>0.9999	>0.9999
C14 vs. T21	0.2459	<0.0001	0.1967	<0.0001	<0.0001	<0.0001
T14 vs. C21	0.0092	<0.0001	0.7508	0.0008	<0.0001	<0.0001
T14 vs. T21	<0.0001	<0.0001	0.0111	<0.0001	<0.0001	<0.0001
C21 vs. T21	0.2459	<0.0001	0.1967	<0.0001	<0.0001	<0.0001
Liver						
C7 vs. T7	0.9586	<0.0001	0.9807	0.9718	0.5041	<0.0001
C7 vs. C14	>0.9999	>0.9999	>0.9999	>0.9999	>0.9999	>0.9999
C7 vs. T14	0.3891	<0.0001	0.9265	<0.0001	<0.0001	<0.0001
C7 vs. C21	>0.9999	>0.9999	>0.9999	>0.9999	>0.9999	>0.9999
C7 vs. T21	0.1296	<0.0001	0.4418	<0.0001	<0.0001	<0.0001
T7 vs. C14	0.9586	<0.0001	0.9807	0.9718	0.5041	<0.0001
T7 vs. T14	0.0923	<0.0001	0.9998	<0.0001	<0.0001	<0.0001
T7 vs. C21	0.9586	<0.0001	0.9807	0.9718	0.5041	<0.0001
T7 vs. T21	0.0222	<0.0001	0.1444	<0.0001	<0.0001	<0.0001
C14 vs. T14	0.3891	<0.0001	0.9265	<0.0001	<0.0001	<0.0001
C14 vs. C21	>0.9999	>0.9999	>0.9999	>0.9999	>0.9999	>0.9999
C14 vs. T21	0.1296	<0.0001	0.4418	<0.0001	<0.0001	<0.0001
T14 vs. C21	0.3891	<0.0001	0.9265	<0.0001	<0.0001	<0.0001
T14 vs. T21	0.9849	<0.0001	0.0863	<0.0001	<0.0001	0.0095
C21 vs. T21	0.1296	<0.0001	0.4418	<0.0001	<0.0001	<0.0001

Supplementary Fig. S7

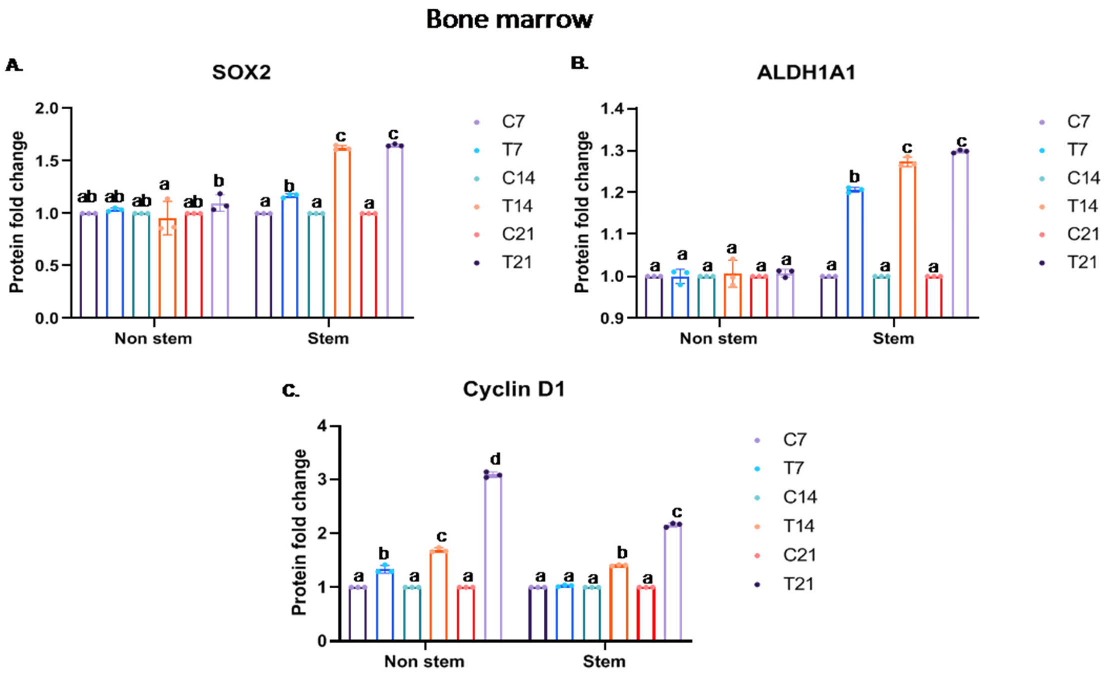

Fig S7. Densitometric quantification of stemness- and proliferation-associated markers shown in Fig. 2L. (A–C) Densitometric analyses of (A) SOX2, (B) ALDH1A1 and (C) Cyclin D1 expression in bone marrow shown in Fig. 2L. Data are presented as mean ± SD (n=3 biological replicates). Statistical significance was determined using two-way ANOVA followed by Tukey's multiple-comparison test. $P < 0.05$ was considered statistically significant. Lowercase letters (a–d) indicate statistically significant differences among groups.

Table S7: Adjusted P values corresponding to Fig. S7A-C

Bone marrow						
C7 vs. T7	0.9558	0.0074	>0.9999	<0.0001	<0.0001	0.8586
C7 vs. C14	>0.9999	>0.9999	>0.9999	>0.9999	>0.9999	>0.9999
C7 vs. T14	0.8905	<0.0001	0.9883	<0.0001	<0.0001	<0.0001
C7 vs. C21	>0.9999	>0.9999	>0.9999	>0.9999	>0.9999	>0.9999
C7 vs. T21	0.2336	<0.0001	0.9676	<0.0001	<0.0001	<0.0001
T7 vs. C14	0.9558	0.0074	>0.9999	<0.0001	<0.0001	0.8586
T7 vs. T14	0.4211	<0.0001	0.9843	<0.0001	<0.0001	<0.0001
T7 vs. C21	0.9558	0.0074	>0.9999	<0.0001	<0.0001	0.8586
T7 vs. T21	0.6957	<0.0001	0.9595	<0.0001	<0.0001	<0.0001
C14 vs. T14	0.8905	<0.0001	0.9883	<0.0001	<0.0001	<0.0001
C14 vs. C21	>0.9999	>0.9999	>0.9999	>0.9999	>0.9999	>0.9999
C14 vs. T21	0.2336	<0.0001	0.9676	<0.0001	<0.0001	<0.0001
T14 vs. C21	0.8905	<0.0001	0.9883	<0.0001	<0.0001	<0.0001
T14 vs. T21	0.0276	0.9891	>0.9999	0.1153	<0.0001	<0.0001
C21 vs. T21	0.2336	<0.0001	0.9676	<0.0001	<0.0001	<0.0001

Supplementary Fig. S8

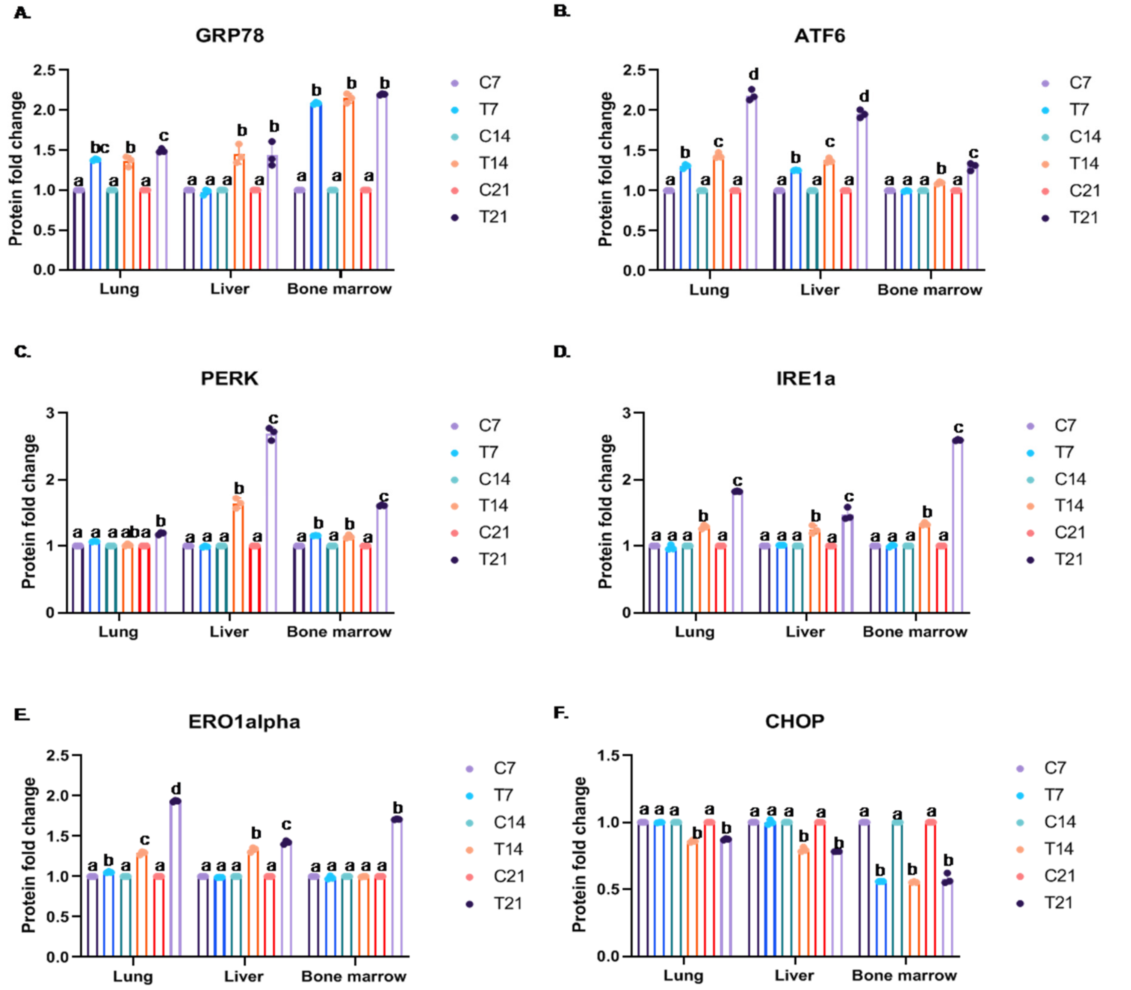

Fig S8. Densitometric quantification of UPR-associated markers shown in Fig. 2M. (A–F) Densitometric analyses of (A) GRP78, (B) ATF6, (C) PERK, (D) IRE1α, (E) Ero1-Lα and (F) CHOP expression in lung, liver and bone marrow shown in Fig. 2M. Data are presented as mean ± SD (n=3 biological replicates). Statistical significance was determined using two-way ANOVA followed by Tukey's multiple-comparison test. $P < 0.05$ was considered statistically significant. Lowercase letters (a–d) indicate statistically significant differences among groups.

Table S8: Adjusted P values corresponding to Fig. S8A-F

	GRP78			ATF6			PERK		
	Lung	Liver	BM	Lung	Liver	BM	Lung	Liver	BM
C7 vs. T7	<0.0001	0.9908	<0.0001	<0.0001	<0.0001	>0.9999	0.1091	0.9981	<0.0001
C7 vs. C14	>0.9999	>0.9999	>0.9999	>0.9999	>0.9999	>0.9999	>0.9999	>0.9999	>0.9999
C7 vs. T14	<0.0001	<0.0001	<0.0001	<0.0001	<0.0001	0.0008	0.9925	<0.0001	<0.0001
C7 vs. C21	>0.9999	>0.9999	>0.9999	>0.9999	>0.9999	>0.9999	>0.9999	>0.9999	>0.9999
C7 vs. T21	<0.0001	<0.0001	<0.0001	<0.0001	<0.0001	<0.0001	<0.0001	<0.0001	<0.0001
T7 vs. C14	<0.0001	0.9908	<0.0001	<0.0001	<0.0001	>0.9999	0.1091	0.9981	<0.0001
T7 vs. T14	0.9962	<0.0001	0.6731	<0.0001	<0.0001	0.0005	0.3143	<0.0001	0.9894
T7 vs. C21	<0.0001	0.9908	<0.0001	<0.0001	<0.0001	>0.9999	0.1091	0.9981	<0.0001
T7 vs. T21	0.1027	<0.0001	0.1027	<0.0001	<0.0001	<0.0001	0.0003	<0.0001	<0.0001
C14 vs. T14	<0.0001	<0.0001	<0.0001	<0.0001	<0.0001	0.0008	0.9925	<0.0001	<0.0001
C14 vs. C21	>0.9999	>0.9999	>0.9999	>0.9999	>0.9999	>0.9999	>0.9999	>0.9999	>0.9999
C14 vs. T21	<0.0001	<0.0001	<0.0001	<0.0001	<0.0001	<0.0001	<0.0001	<0.0001	<0.0001
T14 vs. C21	<0.0001	<0.0001	<0.0001	<0.0001	<0.0001	0.0008	0.9925	<0.0001	<0.0001
T14 vs. T21	0.0334	>0.9999	0.8346	<0.0001	<0.0001	<0.0001	<0.0001	<0.0001	<0.0001
C21 vs. T21	<0.0001	<0.0001	<0.0001	<0.0001	<0.0001	<0.0001	<0.0001	<0.0001	<0.0001

Supplementary Fig. S9

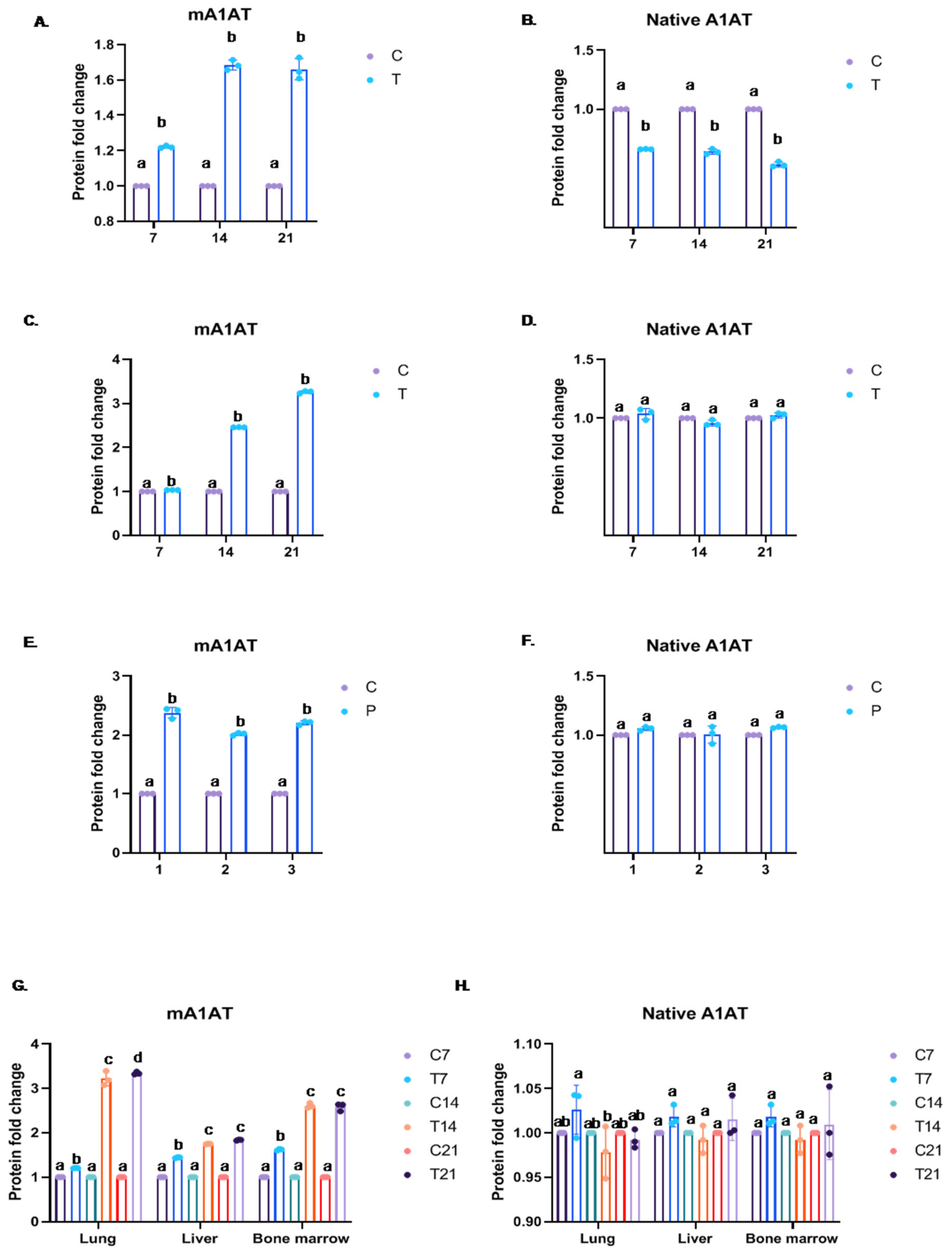

Fig S9. Densitometric quantification of misfolded A1AT and native A1AT shown in Figs. 3E-J. (A–F) Densitometric analyses of misfolded and native A1AT expression shown in Fig. 3E–G, including (A, B) mammary fat pad and mammary tumor tissues from control and tumor-bearing mice at different stages of tumor progression (Fig. 3E), (C, D) serum-derived exosomes isolated from control and tumor-bearing mice at different stages of tumor progression (Fig. 3F), and (E, F) serum-derived exosomes isolated from patients (Fig. 3G). (G, H) Densitometric analyses of (G) misfolded A1AT and (H) native A1AT expression in lung, liver and bone marrow tissues shown in Fig. 3H–J. Data are presented as mean ± SD (n=3 biological replicates). Statistical significance was determined using two-way ANOVA followed by Tukey's multiple-comparison test. $P < 0.05$ was considered statistically significant. Lowercase letters (a–d) indicate statistically significant differences among groups.

Table S9A: Adjusted P values corresponding to Fig. S9A-F

	Mammary tumor		Mammary TDEs		Patient breast TDEs	
C-T	mA1AT	Native A1AT	mA1AT	Native A1AT	mA1AT	Native A1AT
7	<0.0001	<0.0001	0.0092	0.2273	<0.0001	0.1472
14	<0.0001	<0.0001	<0.0001	0.1452	<0.0001	0.9981
21	<0.0001	<0.0001	<0.0001	0.5515	<0.0001	0.0683

Table S9B: Adjusted P values corresponding to Fig. S9G-H

	mA1AT			Native A1AT		
	Lung	Liver	BM	Lung	Liver	BM
C7 vs. T7	<0.0001	<0.0001	<0.0001	0.3645	0.7151	0.7151
C7 vs. C14	>0.9999	>0.9999	>0.9999	>0.9999	>0.9999	>0.9999
C7 vs. T14	<0.0001	<0.0001	<0.0001	0.5548	0.9912	0.9912
C7 vs. C21	>0.9999	>0.9999	>0.9999	>0.9999	>0.9999	>0.9999
C7 vs. T21	<0.0001	<0.0001	<0.0001	0.9919	0.8541	0.9799
T7 vs. C14	<0.0001	<0.0001	<0.0001	0.3645	0.7151	0.7151
T7 vs. T14	<0.0001	<0.0001	<0.0001	0.0093	0.3582	0.3582
T7 vs. C21	<0.0001	<0.0001	<0.0001	0.3645	0.7151	0.7151
T7 vs. T21	<0.0001	<0.0001	<0.0001	0.1305	0.9998	0.9791
C14 vs. T14	<0.0001	<0.0001	<0.0001	0.5548	0.9912	0.9912
C14 vs. C21	>0.9999	>0.9999	>0.9999	>0.9999	>0.9999	>0.9999
C14 vs. T21	<0.0001	<0.0001	<0.0001	0.9919	0.8541	0.9799
T14 vs. C21	<0.0001	<0.0001	<0.0001	0.5548	0.9912	0.9912
T14 vs. T21	0.025	0.2073	0.9836	0.878	0.5131	0.786
C21 vs. T21	<0.0001	<0.0001	<0.0001	0.9919	0.8541	0.9799

Supplementary Fig. S10

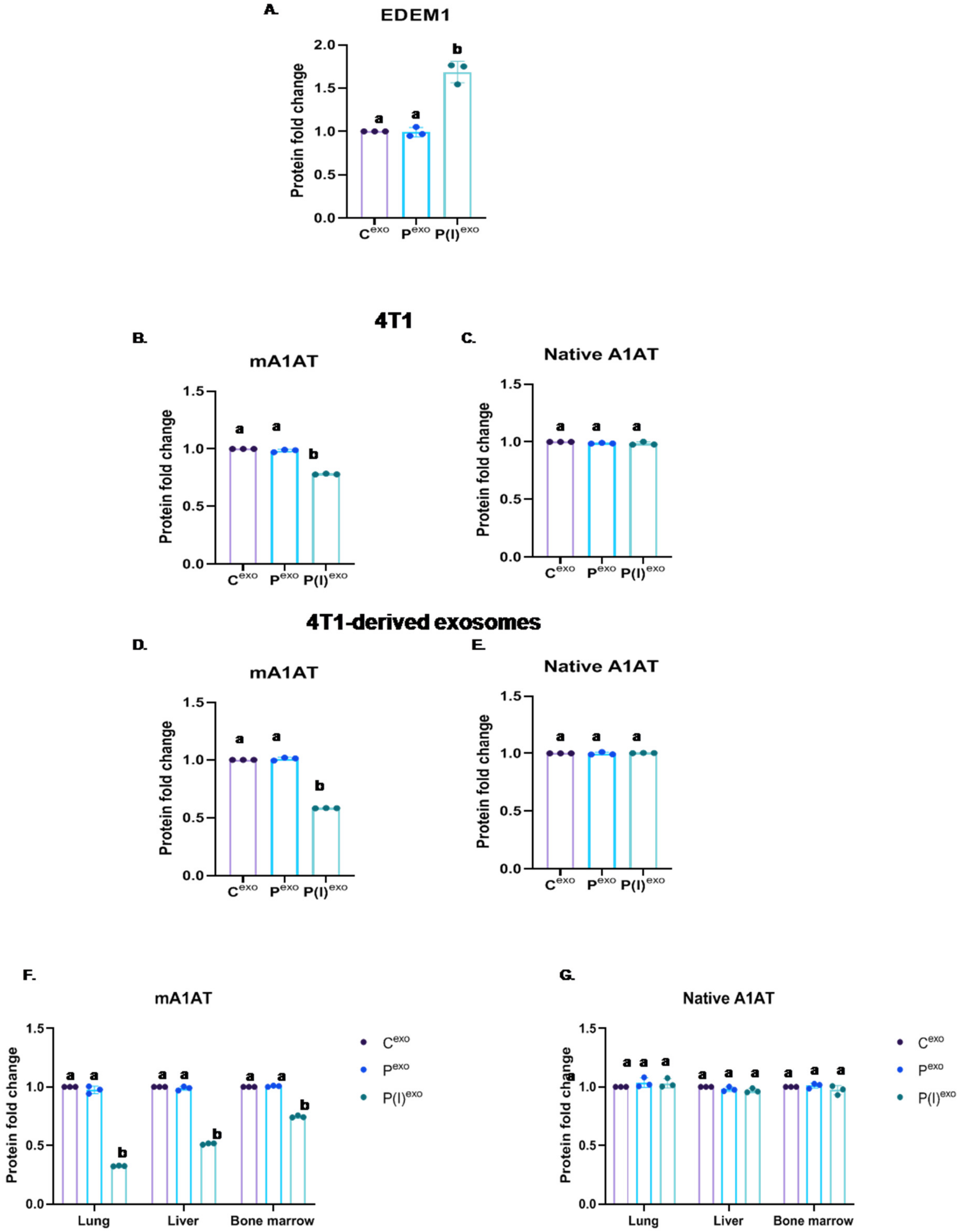

Fig S10. Densitometric quantification of EDEM1, misfolded A1AT and native A1AT shown in Figs. 4B, 4D, 4E-G. (A) Densitometric analyses of EDEM1 expression shown in Fig. 4B, (B-E) Densitometric analyses of (B,D) misfolded and (C,E) native A1AT expression shown in Fig. 4D. Data are presented as mean ± SD (n=3 biological replicates). Statistical significance was determined using one way ANOVA followed by Tukey's multiple-comparison test. $P < 0.05$ was considered statistically significant. Lowercase letters (a–d) indicate statistically significant differences among groups. (F-G) Densitometric analyses of (F) misfolded and (G) native A1AT expression shown in Fig. 4E-G. Data are presented as mean ± SD (n=3 biological replicates). Statistical significance was determined using two-way ANOVA followed by Tukey's multiple-comparison test. $P < 0.05$ was considered statistically significant. Lowercase letters (a–d) indicate statistically significant differences among groups.

Table S10A: Adjusted P values corresponding to Fig. S10A

		C^{exo} vs. P^{exo}	C^{exo} vs. $P(I)^{exo}$	P^{exo} vs. $P(I)^{exo}$
EDEM1	4T1	0.9895	<0.0001	<0.0001

Table S10B: Adjusted P values corresponding to Fig. S10B-E

		C^{exo} vs. P^{exo}	C^{exo} vs. $P(I)^{exo}$	P^{exo} vs. $P(I)^{exo}$
mA1AT	4T1	0.0936	<0.0001	<0.0001
	4T1-derived exosomes	0.4058	<0.0001	<0.0001
Native A1AT	4T1	0.2752	0.1855	0.9466
	4T1-derived exosomes	0.9349	0.8075	0.6114

Table S10C: Adjusted P values corresponding to Fig. S10F-G

		C^{exo} vs. P^{exo}	C^{exo} vs. $P(I)^{exo}$	P^{exo} vs. $P(I)^{exo}$
mA1AT	Lung	0.0571	<0.0001	<0.0001
	Liver	0.4802	<0.0001	<0.0001
	BM	0.8113	<0.0001	<0.0001
Native A1AT	Lung	0.2203	0.3166	0.9692
	Liver	0.6229	0.3099	0.8376
	BM	0.8855	0.3968	0.197

Supplementary Fig. S11

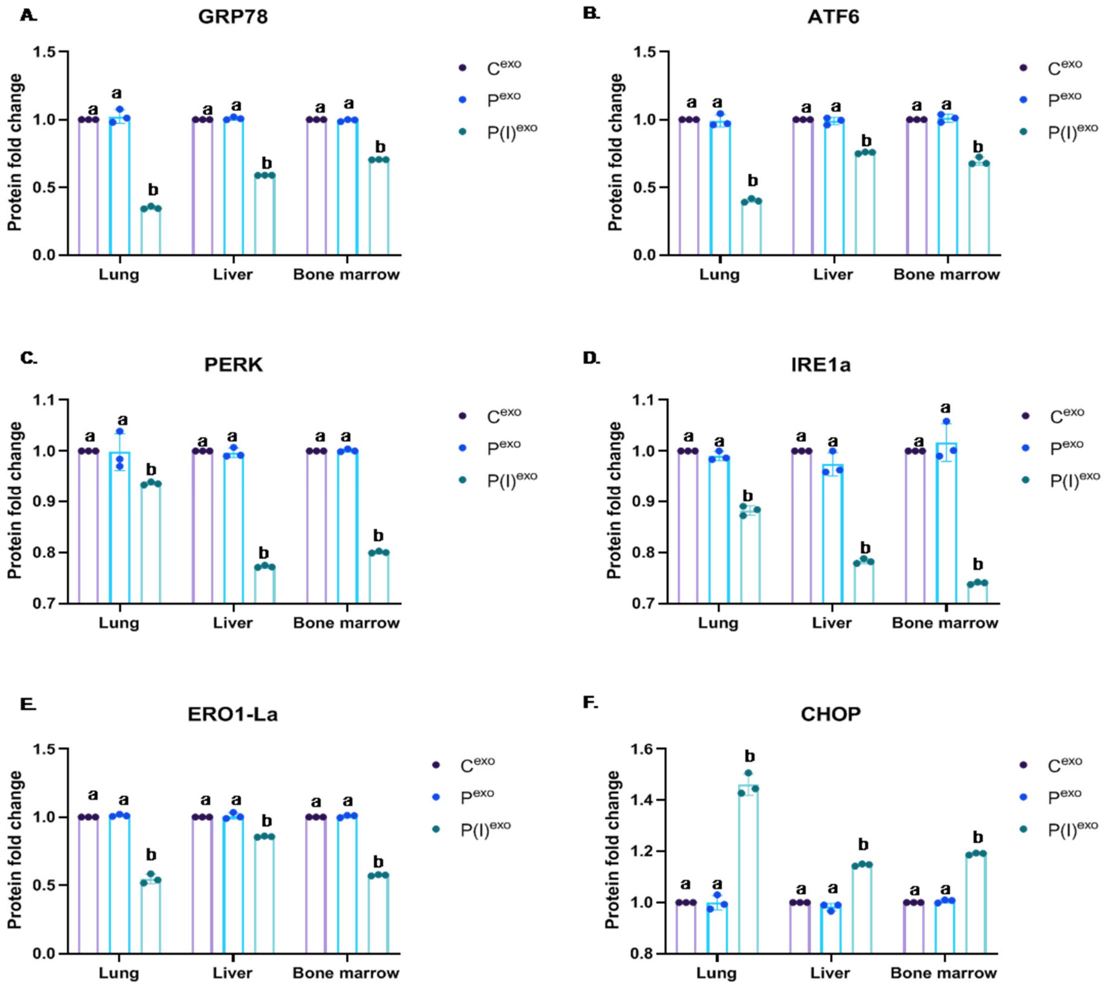

Fig S11. Densitometric quantification of UPR-associated markers shown in Figs. 4H. (A–F) Densitometric analyses of (A) GRP78, (B) ATF6, (C) PERK, (D) IRE1α, (E) Ero1-Lα and (F) CHOP expression in lung, liver and bone marrow shown in Fig. 4H. Data are presented as mean ± SD (n=3 biological replicates). Statistical significance was determined using two-way ANOVA followed by Tukey's multiple-comparison test. $P < 0.05$ was considered statistically significant. Lowercase letters (a,b) indicate statistically significant differences among groups.

Table S11: Adjusted P values corresponding to Fig. S11A-F

		C^{exo} vs. P^{exo}	C^{exo} vs. $P(I)^{exo}$	P^{exo} vs. $P(I)^{exo}$
GRP78	Lung	0.268	<0.0001	<0.0001
	Liver	0.8181	<0.0001	<0.0001
	BM	0.9281	<0.0001	<0.0001
ATF6	Lung	0.8967	<0.0001	<0.0001
	Liver	0.8307	<0.0001	<0.0001
	BM	0.8567	<0.0001	<0.0001
PERK	Lung	0.9647	<0.0001	<0.0001
	Liver	0.9236	<0.0001	<0.0001
	BM	0.9969	<0.0001	<0.0001
IRE1α	Lung	0.6904	<0.0001	<0.0001
	Liver	0.1126	<0.0001	<0.0001
	BM	0.403	<0.0001	<0.0001
ERO1-Lα	Lung	0.579	<0.0001	<0.0001
	Liver	0.7583	<0.0001	<0.0001
	BM	0.8613	<0.0001	<0.0001
CHOP	Lung	0.996	<0.0001	<0.0001
	Liver	0.4311	<0.0001	<0.0001
	BM	0.934	<0.0001	<0.0001

Supplementary Fig. S12

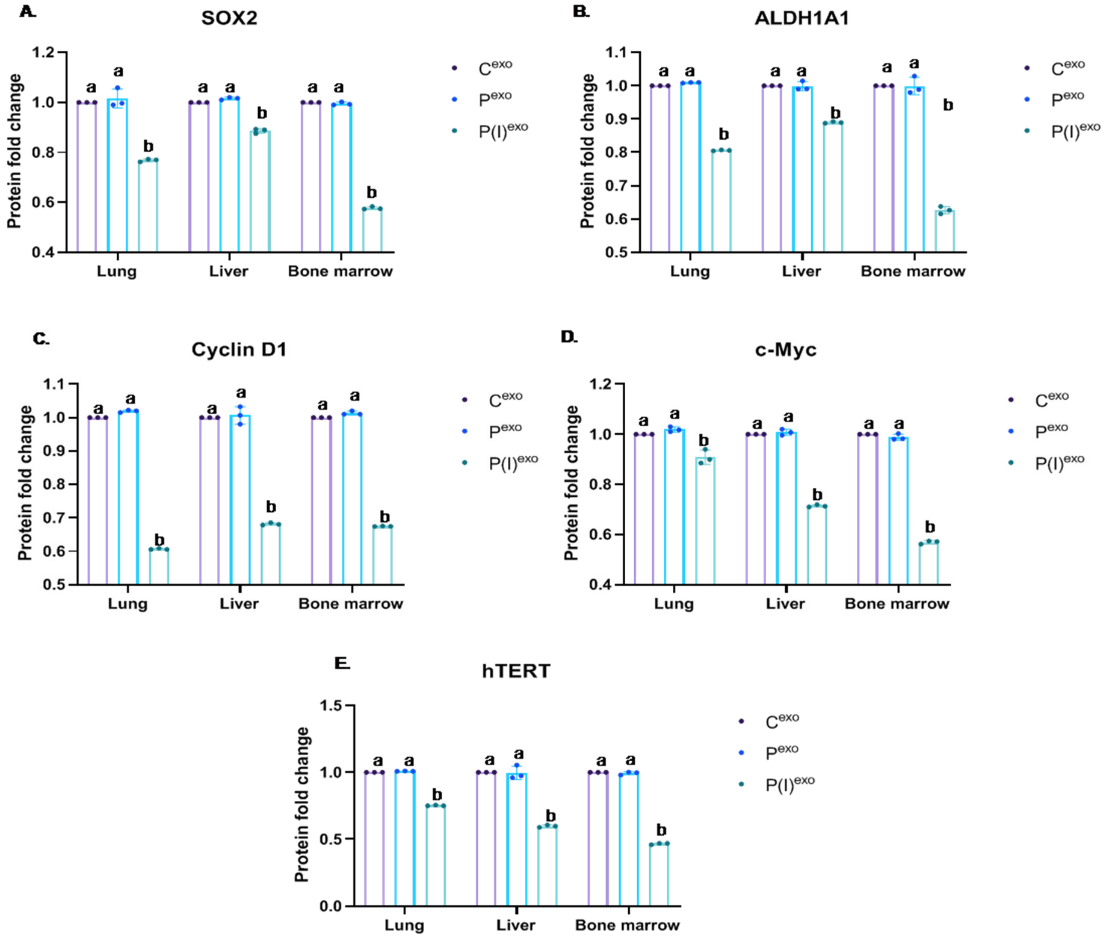

Fig S12. Densitometric quantification of stemness- and proliferation-associated markers shown in Figs. 4I. (A–E) Densitometric analyses of (A) SOX2, (B) ALDH1A1, (C) Cyclin D1, (D) cMyc and (E) hTERT expression in lung, liver and bone marrow shown in Fig. 4I. Data are presented as mean ± SD (n=3 biological replicates). Statistical significance was determined using two-way ANOVA followed by Tukey's multiple-comparison test. $P < 0.05$ was considered statistically significant. Lowercase letters (a,b) indicate statistically significant differences among groups.

Table S12: Adjusted P values corresponding to Fig. S12A-E

		C^{exo} vs. P^{exo}	C^{exo} vs. $P(I)^{exo}$	P^{exo} vs. $P(I)^{exo}$
SOX2	Lung	0.3794	<0.0001	<0.0001
	Liver	0.3666	<0.0001	<0.0001
	BM	0.8773	<0.0001	<0.0001
ALDH1A1	Lung	0.5537	<0.0001	<0.0001
	Liver	0.9815	<0.0001	<0.0001
	BM	0.9923	<0.0001	<0.0001
Cyclin D1	Lung	0.0504	<0.0001	<0.0001
	Liver	0.699	<0.0001	<0.0001
	BM	0.2121	<0.0001	<0.0001
c-Myc	Lung	0.1603	<0.0001	<0.0001
	Liver	0.7155	<0.0001	<0.0001
	BM	0.4515	<0.0001	<0.0001
hTERT	Lung	0.8324	<0.0001	<0.0001
	Liver	0.9605	<0.0001	<0.0001
	BM	0.8831	<0.0001	<0.0001

Supplementary Fig. S13

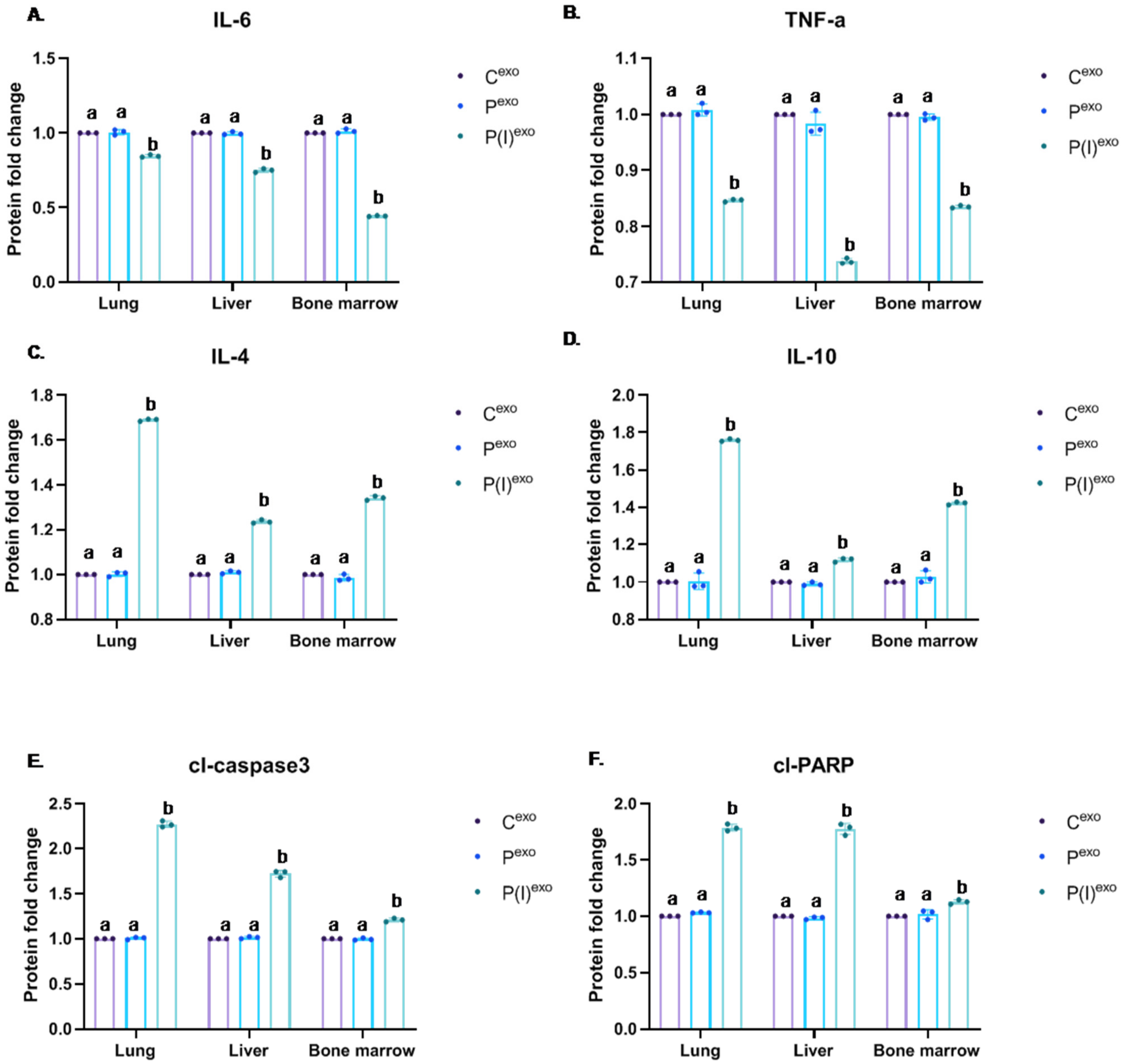

Fig S13. Densitometric quantification of inflammation- and apoptosis-associated markers shown in Figs. 4J and 4K. (A–D) Densitometric analyses of (A) IL-6, (B) TNF-α, (C) IL-4 and (D) IL-10 expression in lung, liver and bone marrow shown in Fig. 4J. (E–F) Densitometric analyses of (E) Cl-caspase3 and (F) Cl-PARP expression in lung, liver and bone marrow shown in Fig. 4K. Data are presented as mean ± SD (n=3 biological replicates). Statistical significance was determined using two-way ANOVA followed by Tukey's multiple-comparison test. $P < 0.05$ was considered statistically significant. Lowercase letters (a,b) indicate statistically significant differences among groups.

Table S13: Adjusted P values corresponding to Fig. S13A-F

		C^{exo} vs. P^{exo}	C^{exo} vs. $P(I)^{exo}$	P^{exo} vs. $P(I)^{exo}$
IL-6	Lung	0.7466	<0.0001	<0.0001
	Liver	0.945	<0.0001	<0.0001
	BM	0.3024	<0.0001	<0.0001
TNF-α	Lung	0.4913	<0.0001	<0.0001
	Liver	0.0566	<0.0001	<0.0001
	BM	0.7382	<0.0001	<0.0001
IL-4	Lung	0.9688	<0.0001	<0.0001
	Liver	0.2961	<0.0001	<0.0001
	BM	0.0617	<0.0001	<0.0001
IL-10	Lung	0.9727	<0.0001	<0.0001
	Liver	0.7493	<0.0001	<0.0001
	BM	0.2409	<0.0001	<0.0001
cl-caspase3	Lung	0.9385	<0.0001	<0.0001
	Liver	0.7072	<0.0001	<0.0001
	BM	0.9674	<0.0001	<0.0001
cl-PARP	Lung	0.2799	<0.0001	<0.0001
	Liver	0.7152	<0.0001	<0.0001
	BM	0.664	<0.0001	0.0001

Supplementary Fig. S14

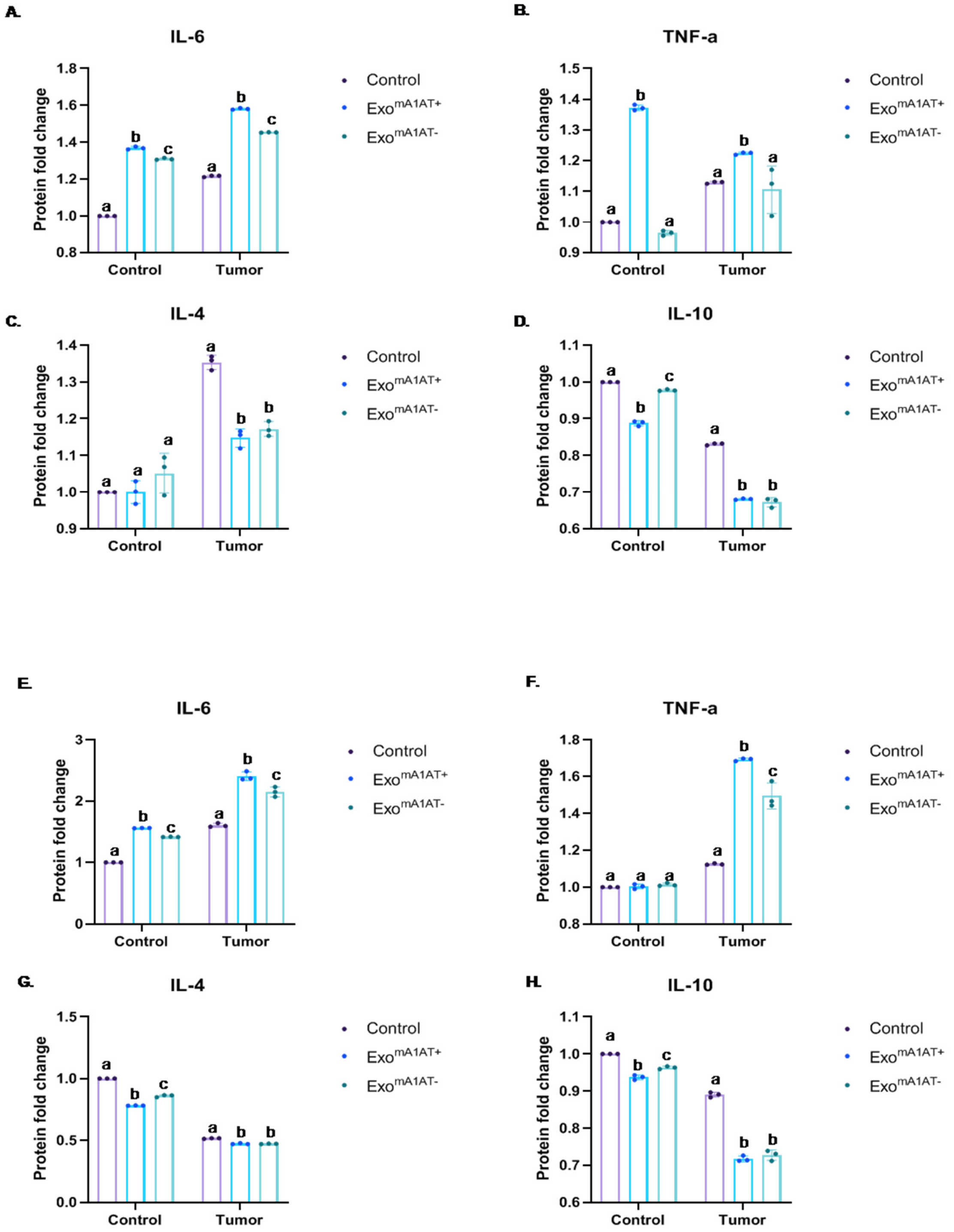

Fig S14. Densitometric quantification of inflammation- associated markers shown in Figs. 5I and 5J. (A–D) Densitometric analyses of (A) IL-6, (B) TNF-α, (C) IL-4 and (D) IL-10 expression in lung tissue shown in Fig. 5I. (E–H) Densitometric analyses of (E) IL-6, (F) TNF-α, (G) IL-4 and (H) IL-10 expression in liver tissue shown in Fig. 5J. Data are presented as mean ± SD (n=3 biological replicates). Statistical significance was determined using two-way ANOVA followed by Tukey's multiple-comparison test. $P < 0.05$ was considered statistically significant. Lowercase letters (a-c) indicate statistically significant differences among groups.

Table S14: Adjusted P values corresponding to Fig. S14A-H

	IL-6		TNF-α		IL-4		IL-10	
Lung	Control	Tumor	Control	Tumor	Control	Tumor	Control	Tumor
Control vs. ExomA1AT+	<0.0001	<0.0001	<0.0001	0.0088	0.9999	<0.0001	<0.0001	<0.0001
Control vs. ExomA1AT-	<0.0001	<0.0001	0.3843	0.6498	0.1259	<0.0001	0.002	<0.0001
ExomA1AT+ vs. ExomA1AT-	<0.0001	<0.0001	<0.0001	0.0018	0.1228	0.5792	<0.0001	0.2181
	IL-6		TNF-α		IL-4		IL-10	
Liver	Control	Tumor	Control	Tumor	Control	Tumor	Control	Tumor
Control vs. ExomA1AT+	<0.0001	<0.0001	0.9908	<0.0001	<0.0001	<0.0001	<0.0001	<0.0001
Control vs. ExomA1AT-	<0.0001	<0.0001	0.8564	<0.0001	<0.0001	<0.0001	0.0002	<0.0001
ExomA1AT+ vs. ExomA1AT-	0.0058	<0.0001	0.9146	<0.0001	<0.0001	0.9939	0.0035	0.2916

Supplementary Fig. S15

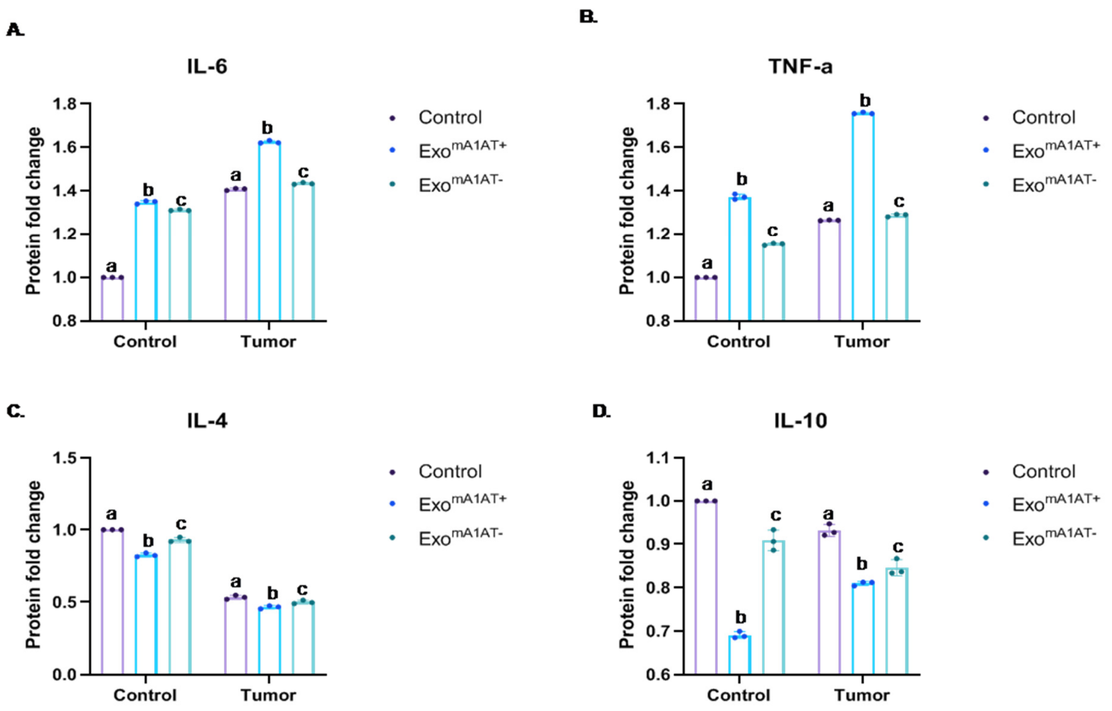

Fig S15. Densitometric quantification of inflammation- associated markers shown in Fig. 5K. (A–D) Densitometric analyses of (A) IL-6, (B) TNF-α, (C) IL-4 and (D) IL-10 expression in bone marrow shown in Fig. 5K. Data are presented as mean ± SD (n=3 biological replicates). Statistical significance was determined using two-way ANOVA followed by Tukey's multiple-comparison test. $P < 0.05$ was considered statistically significant. Lowercase letters (a-c) indicate statistically significant differences among groups.

Table S15: Adjusted P values corresponding to Fig. S15A-D

BM	IL-6		TNF-α		IL-4		IL-10	
	Control	Tumor	Control	Tumor	Control	Tumor	Control	Tumor
Control vs. ExomA1AT+	<0.0001	<0.0001	<0.0001	<0.0001	<0.0001	<0.0001	<0.0001	<0.0001
Control vs. ExomA1AT-	<0.0001	0.0001	<0.0001	0.002	<0.0001	0.0176	<0.0001	<0.0001
ExomA1AT+ vs. ExomA1AT-	<0.0001	<0.0001	<0.0001	<0.0001	<0.0001	0.0136	<0.0001	0.0205

Supplementary Fig. S16

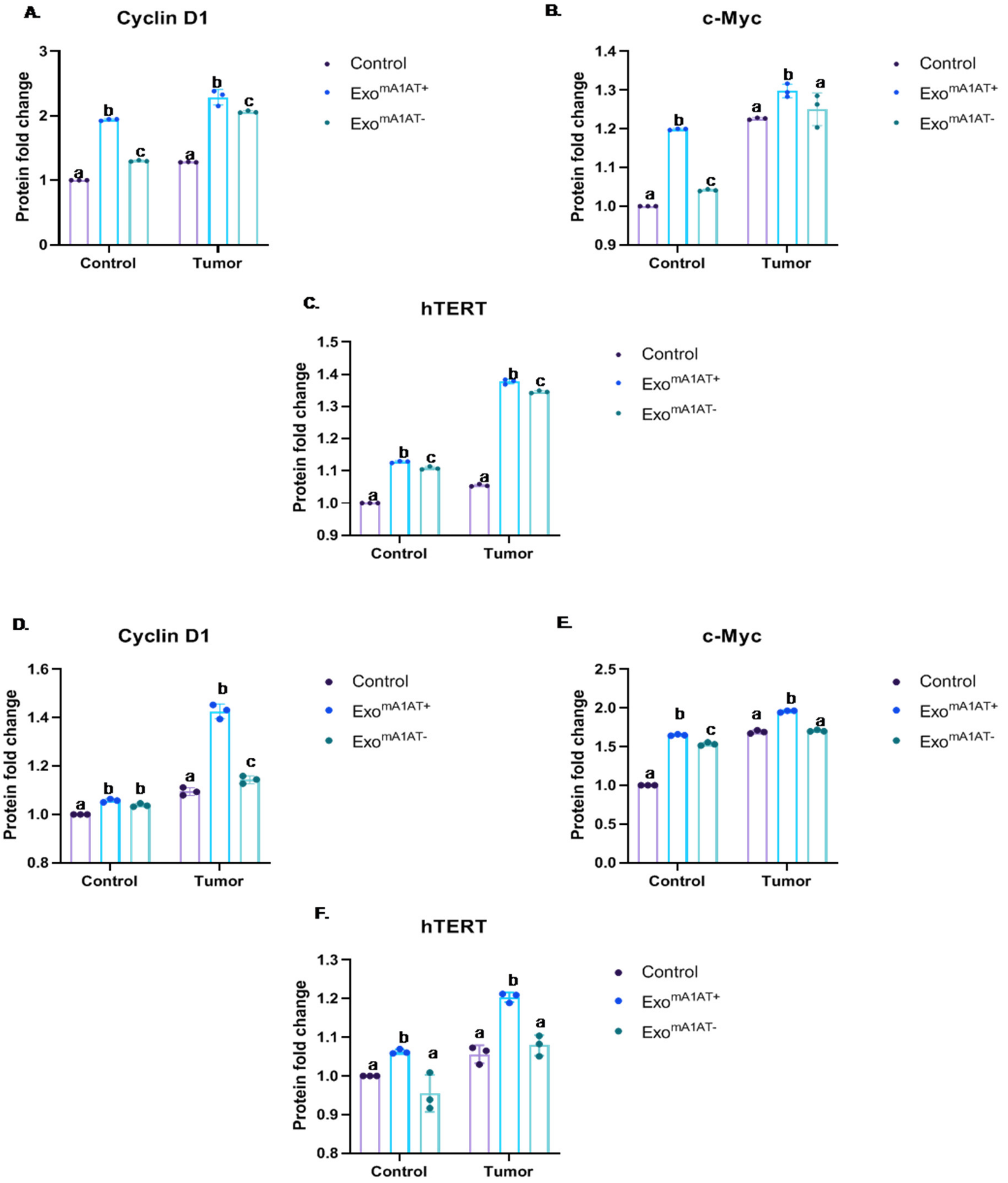

Fig S16. Densitometric quantification of proliferation- associated markers shown in Figs. 6D and 6E. (A–C) Densitometric analyses of (A) Cyclin D1, (B) cMyc and (C) hTERT expression in lung tissue shown in Fig. 6D. (D-F) Densitometric analyses of (D) Cyclin D1, (E) cMyc and (F) hTERT expression in liver tissue shown in Fig. 6E. Data are presented as mean ± SD (n=3 biological replicates). Statistical significance was determined using two-way ANOVA followed by Tukey's multiple-comparison test. $P < 0.05$ was considered statistically significant. Lowercase letters (a-c) indicate statistically significant differences among groups.

Table S16: Adjusted P values corresponding to Fig. S16A-F

Lung	Cyclin D1		c-Myc		hTERT	
	Control	Tumor	Control	Tumor	Control	Tumor
Control vs. ExomA1AT+	<0.0001	<0.0001	<0.0001	0.0014	<0.0001	<0.0001
Control vs. ExomA1AT-	<0.0001	<0.0001	0.0479	0.2844	<0.0001	<0.0001
ExomA1AT+ vs. ExomA1AT-	<0.0001	0.0004	<0.0001	0.0231	0.0004	<0.0001
Liver	Cyclin D1		c-Myc		hTERT	
	Control	Tumor	Control	Tumor	Control	Tumor
Control vs. ExomA1AT+	0.0022	<0.0001	<0.0001	<0.0001	0.0245	<0.0001
Control vs. ExomA1AT-	0.0317	0.0069	<0.0001	0.4684	0.1055	0.5138
ExomA1AT+ vs. ExomA1AT-	0.3285	<0.0001	<0.0001	<0.0001	0.0005	0.0001

Supplementary Fig. S17

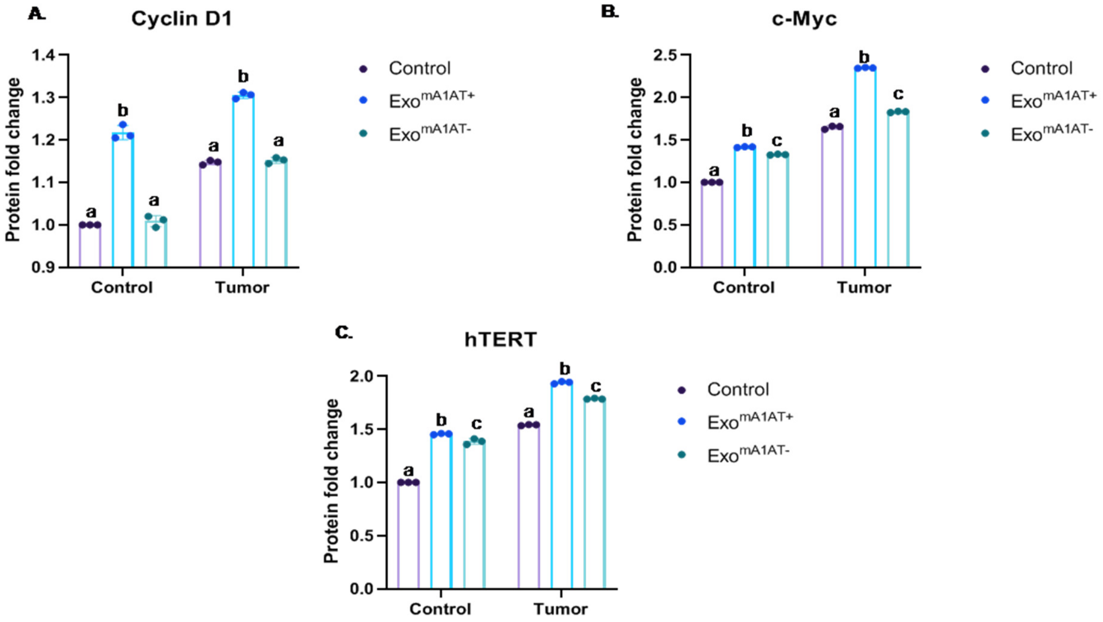

Fig S17. Densitometric quantification of proliferation- associated markers shown in Fig. 6F. (A–C) Densitometric analyses of (A) Cyclin D1, (B) cMyc and (C) hTERT expression in bone marrow shown in Fig. 6F. Data are presented as mean ± SD (n=3 biological replicates). Statistical significance was determined using two-way ANOVA followed by Tukey's multiple-comparison test. $P < 0.05$ was considered statistically significant. Lowercase letters (a-c) indicate statistically significant differences among groups.

Table S17: Adjusted P values corresponding to Fig. S17A-C

BM	Cyclin D1		c-Myc		hTERT	
	Control	Tumor	Control	Tumor	Control	Tumor
Control vs. ExomA1AT+	<0.0001	<0.0001	<0.0001	<0.0001	<0.0001	<0.0001
Control vs. ExomA1AT-	0.5568	0.8048	<0.0001	<0.0001	<0.0001	<0.0001
ExomA1AT+ vs. ExomA1AT-	<0.0001	<0.0001	<0.0001	<0.0001	<0.0001	<0.0001

Supplementary Fig. S18

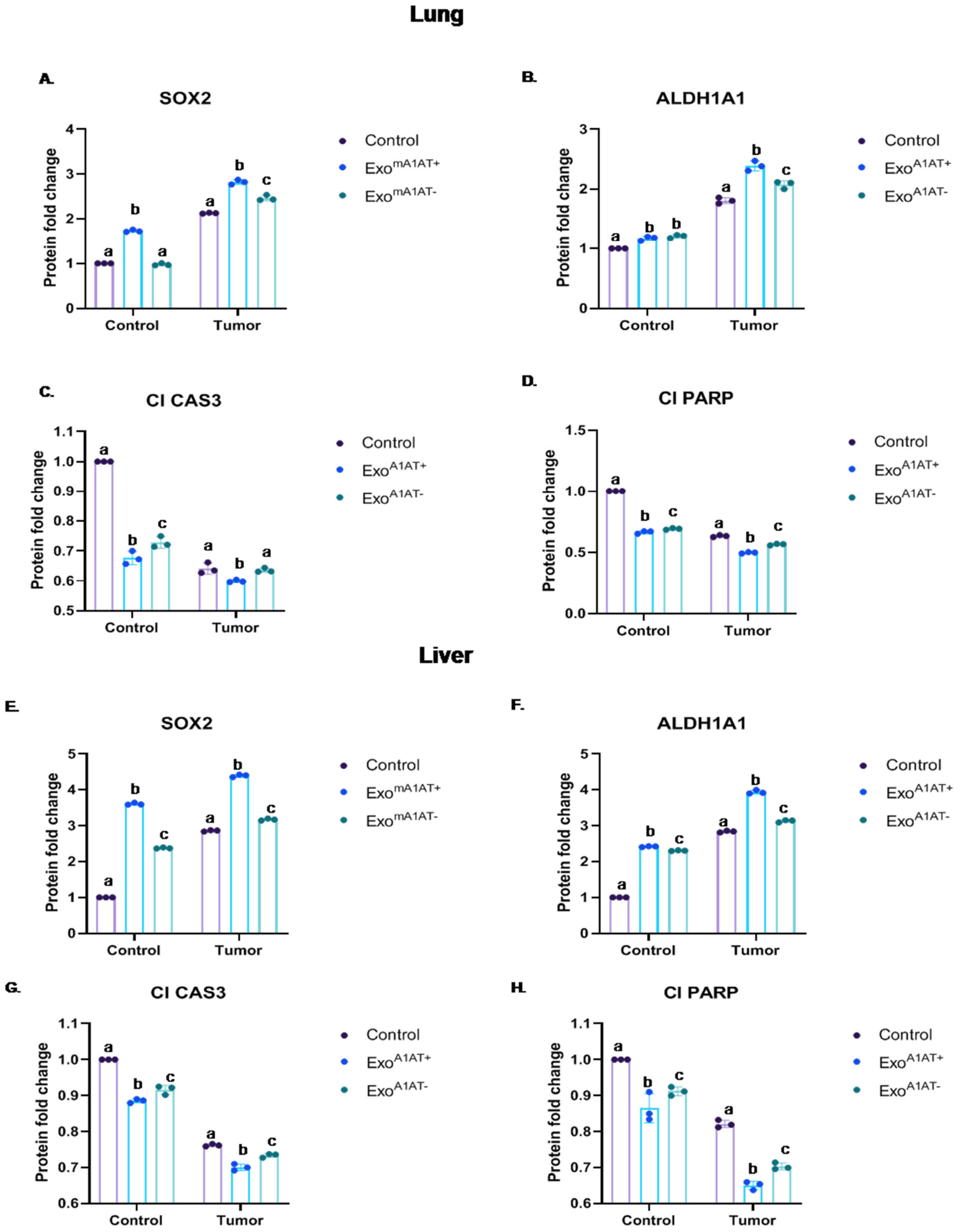

Fig S18. Densitometric quantification of stemness- and apoptosis- associated markers from lung and liver shown in Fig. 6G. (A–D) Densitometric analyses of (A) SOX2, (B) ALDH1A1, (C) Cl-caspase3 and (D) Cl-PARP expression in lung shown in Fig. 6G. (E–H) Densitometric analyses of (E) SOX2, (F) ALDH1A1, (G) Cl-caspase3 and (H) Cl-PARP expression in liver shown in Fig. 6G. Data are presented as mean ± SD (n=3 biological replicates). Statistical significance was determined using two-way ANOVA followed by Tukey's multiple-comparison test. $P < 0.05$ was considered statistically significant. Lowercase letters (a-c) indicate statistically significant differences among groups.

Table S18: Adjusted P values corresponding to Fig. S18A-H

	SOX2		ALDH1A1		Cl Caspase3		Cl PARP	
Lung	Control	Tumor	Control	Tumor	Control	Tumor	Control	Tumor
Control vs. ExomA1AT+	<0.0001	<0.0001	0.0035	<0.0001	<0.0001	0.0102	<0.0001	<0.0001
Control vs. ExomA1AT-	0.6948	<0.0001	0.0008	<0.0001	<0.0001	0.8863	<0.0001	<0.0001
ExomA1AT+ vs. ExomA1AT-	<0.0001	<0.0001	0.6784	<0.0001	0.0024	0.0237	0.0016	<0.0001
	SOX2		ALDH1A1		Cl Caspase3		Cl PARP	
Liver	Control	Tumor	Control	Tumor	Control	Tumor	Control	Tumor
Control vs. ExomA1AT+	<0.0001	<0.0001	<0.0001	<0.0001	<0.0001	<0.0001	<0.0001	<0.0001
Control vs. ExomA1AT-	<0.0001	<0.0001	<0.0001	<0.0001	<0.0001	0.001	0.0002	<0.0001
ExomA1AT+ vs. ExomA1AT-	<0.0001	<0.0001	0.0011	<0.0001	0.0006	0.0003	0.0241	0.012

Supplementary Fig. S19

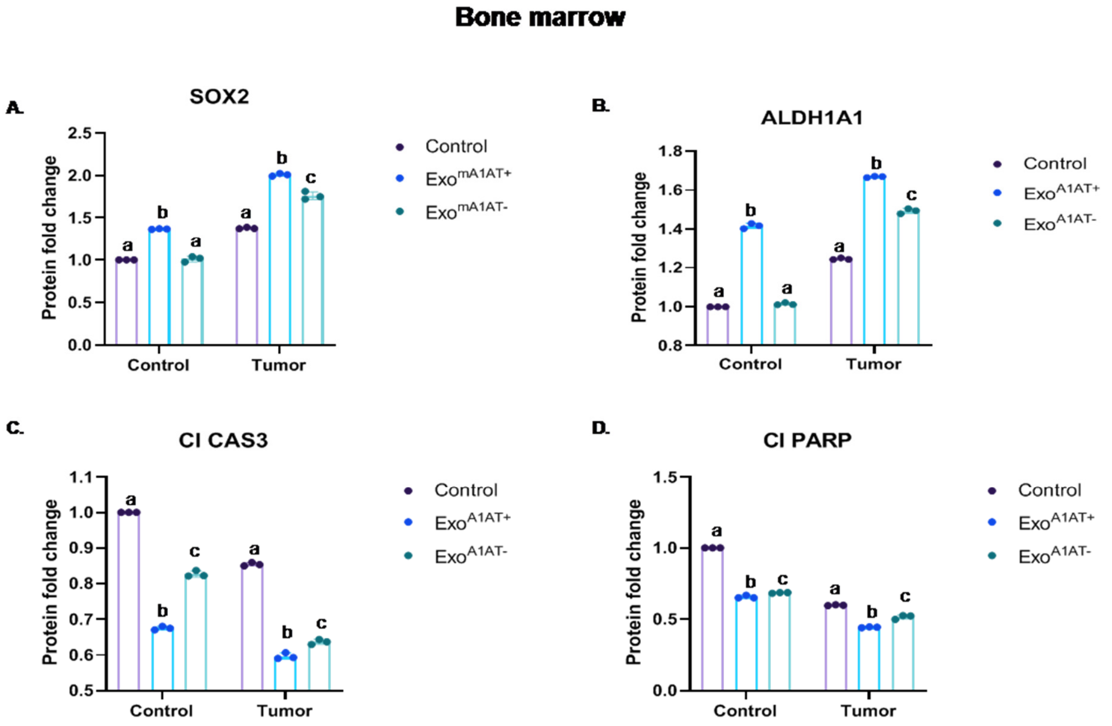

Fig S19. Densitometric quantification of stemness- and apoptosis- associated markers from bone marrow shown in Fig. 6G. (A–D) Densitometric analyses of (A) SOX2, (B) ALDH1A1, (C) Cl-caspase3 and (D) Cl-PARP expression in bone marrow shown in Fig. 6G. Data are presented as mean ± SD (n=3 biological replicates). Statistical significance was determined using two-way ANOVA followed by Tukey's multiple-comparison test. P < 0.05 was considered statistically significant. Lowercase letters (a-c) indicate statistically significant differences among groups.

Table S19: Adjusted P values corresponding to Fig. S19A-D

BM	SOX2		ALDH1A1		Cl Caspase3		Cl PARP	
	Control	Tumor	Control	Tumor	Control	Tumor	Control	Tumor
Control vs. ExomA1AT+	<0.0001	<0.0001	<0.0001	<0.0001	<0.0001	<0.0001	<0.0001	<0.0001
Control vs. ExomA1AT-	0.8389	<0.0001	0.1295	<0.0001	<0.0001	<0.0001	<0.0001	<0.0001
ExomA1AT+ vs. ExomA1AT-	<0.0001	<0.0001	<0.0001	<0.0001	<0.0001	<0.0001	0.0016	<0.0001

Supplementary Fig. S20

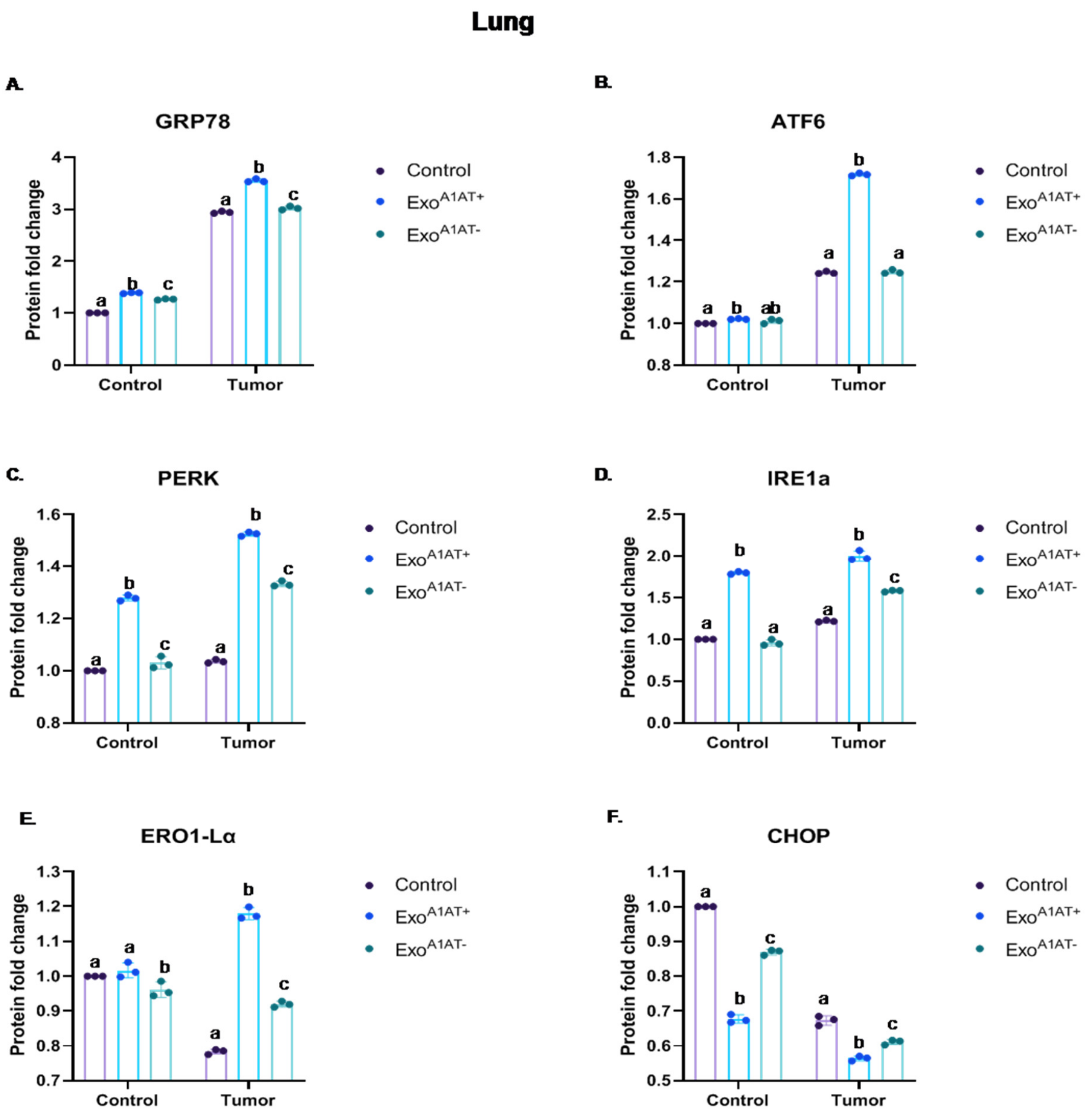

Fig S20. Densitometric quantification of UPR-associated markers from lung shown in Figs. 6H. (A–F) Densitometric analyses of (A) GRP78, (B) ATF6, (C) PERK, (D) IRE1α, (E) Ero1-Lα and (F) CHOP expression in lung shown in Fig. 6H. Data are presented as mean ± SD (n=3 biological replicates). Statistical significance was determined using two-way ANOVA followed by Tukey's multiple-comparison test. P < 0.05 was considered statistically significant. Lowercase letters (a-c) indicate statistically significant differences among groups.

Table S20: Adjusted P values corresponding to Fig. S20A-F

Lung	GRP78		ATF6		PERK		IRE1α		ERO1-Lα		CHOP	
	Control	Tumor	Control	Tumor	Control	Tumor	Control	Tumor	Control	Tumor	Control	Tumor
Control vs. ExomA1AT+	<0.0001	<0.0001	0.0083	<0.0001	<0.0001	<0.0001	<0.0001	<0.0001	0.4014	<0.0001	<0.0001	<0.0001
Control vs. ExomA1AT-	<0.0001	0.002	0.1892	0.8485	0.0267	<0.0001	0.2586	<0.0001	0.0194	<0.0001	<0.0001	<0.0001
ExomA1AT+ vs. ExomA1AT-	<0.0001	<0.0001	0.2113	<0.0001	<0.0001	<0.0001	<0.0001	<0.0001	0.0018	<0.0001	<0.0001	<0.0001

Supplementary Fig. S21

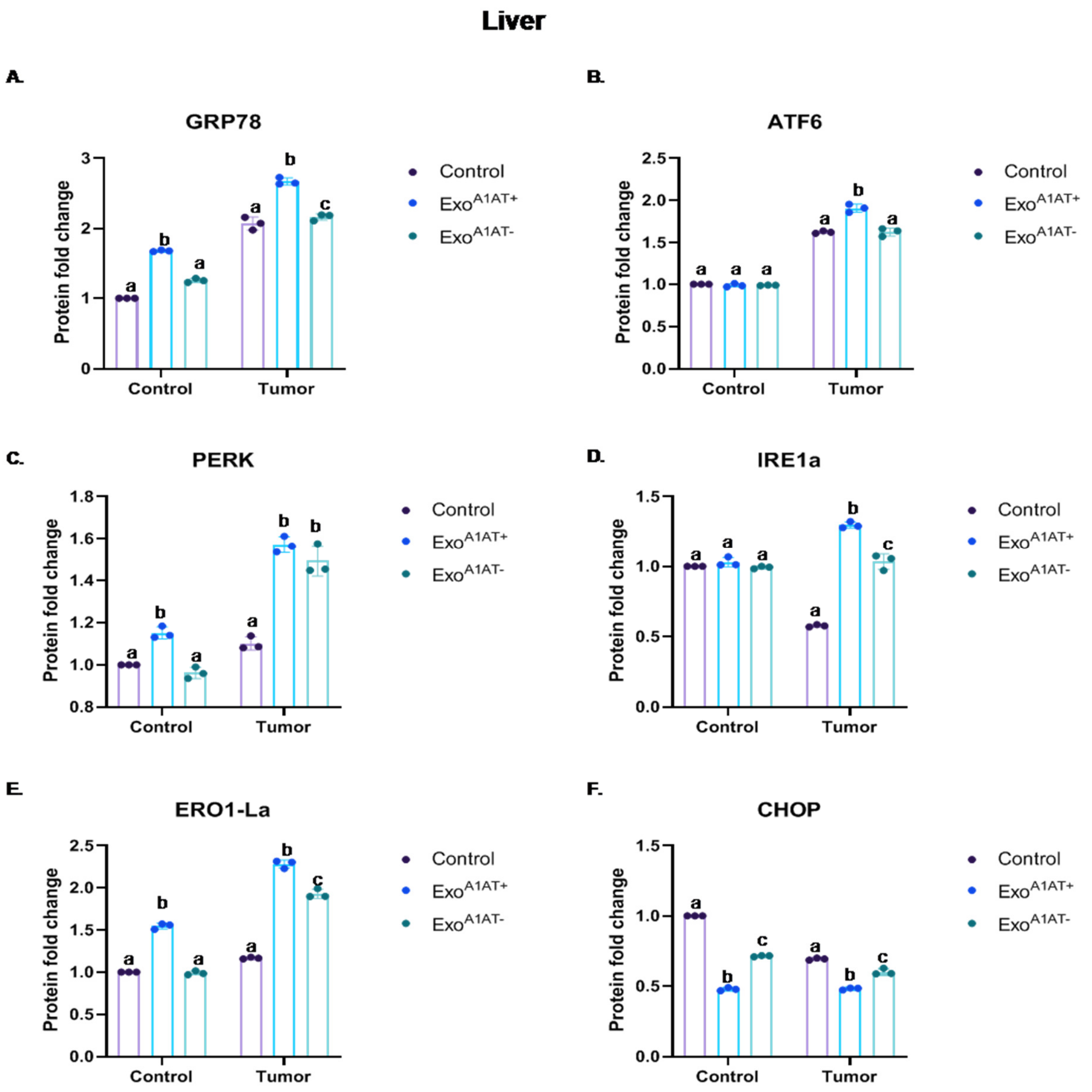

Fig S21. Densitometric quantification of UPR-associated markers from liver shown in Figs. 6H. (A–F) Densitometric analyses of (A) GRP78, (B) ATF6, (C) PERK, (D) IRE1α, (E) Ero1-Lα and (F) CHOP expression in liver shown in Fig. 6H. Data are presented as mean ± SD (n=3 biological replicates). Statistical significance was determined using two-way ANOVA followed by Tukey's multiple-comparison test. P < 0.05 was considered statistically significant. Lowercase letters (a-c) indicate statistically significant differences among groups.

Table S21: Adjusted P values corresponding to Fig. S21A-F

	GRP78		ATF6		PERK		IRE1α		ERO1-Lα		CHOP	
Liver	Control	Tumor	Control	Tumor	Control	Tumor	Control	Tumor	Control	Tumor	Control	Tumor
Control vs. ExomA1AT+	<0.0001	<0.0001	0.852	<0.0001	0.0013	<0.0001	0.426	<0.0001	<0.0001	<0.0001	<0.0001	<0.0001
Control vs. ExomA1AT-	<0.0001	0.0879	0.875	0.9924	0.4657	<0.0001	0.951	<0.0001	0.9268	<0.0001	<0.0001	<0.0001
ExomA1AT+ vs. ExomA1AT-	<0.0001	<0.0001	0.999	<0.0001	0.0002	0.0745	0.285	<0.0001	<0.0001	<0.0001	<0.0001	<0.0001

Supplementary Fig. S22

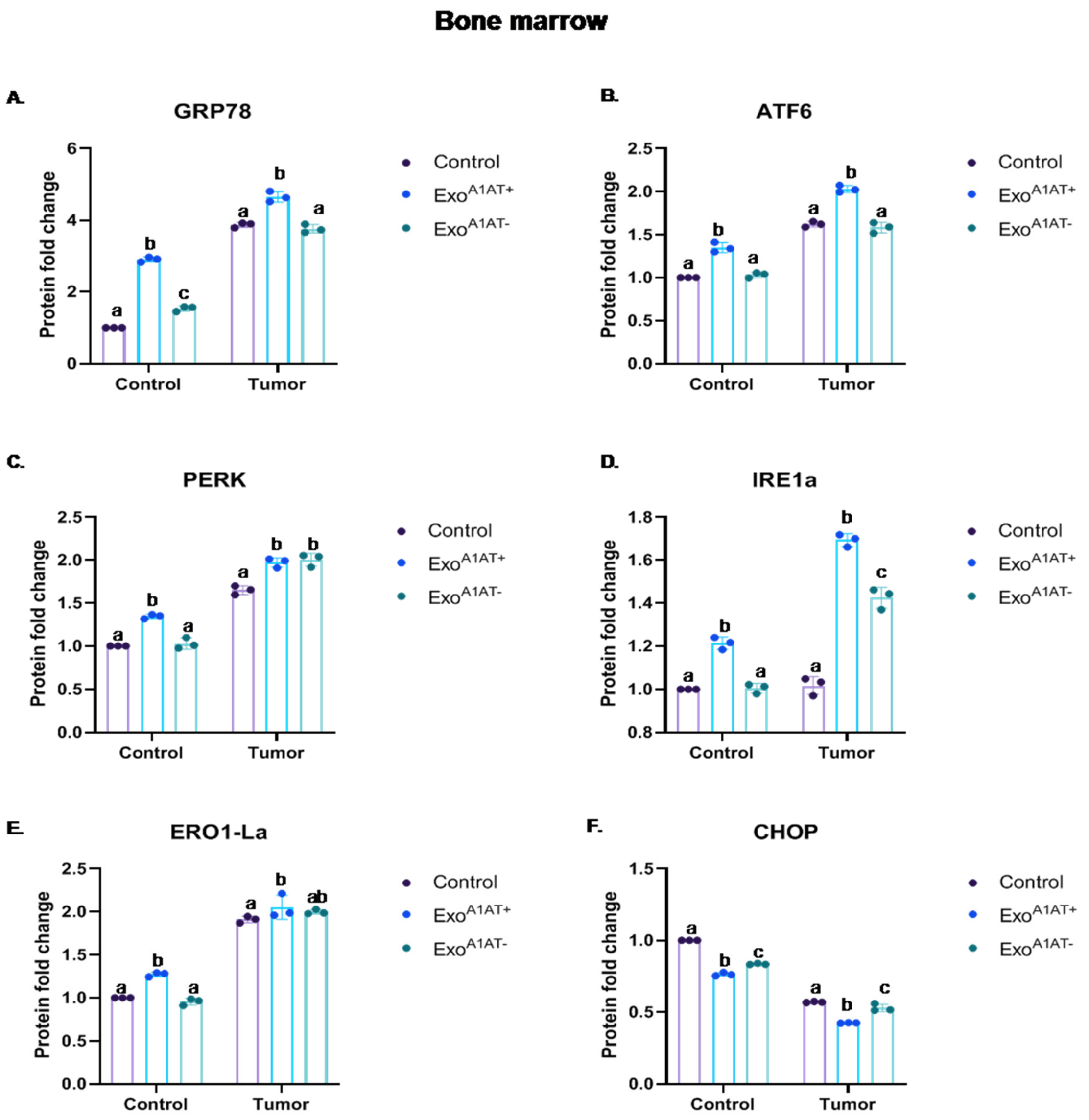

Fig S22. Densitometric quantification of UPR-associated markers from bone marrow shown in Figs. 6H. (A–F) Densitometric analyses of (A) GRP78, (B) ATF6, (C) PERK, (D) IRE1α, (E) Ero1-Lα and (F) CHOP expression in bone marrow shown in Fig. 6H. Data are presented as mean ± SD (n=3 biological replicates). Statistical significance was determined using two-way ANOVA followed by Tukey's multiple-comparison test. $P < 0.05$ was considered statistically significant. Lowercase letters (a-c) indicate statistically significant differences among groups.

Table S22: Adjusted P values corresponding to Fig. S22A-F

	GRP78		ATF6		PERK		IRE1α		ERO1-Lα		CHOP	
BM	Control	Tumor	Control	Tumor	Control	Tumor	Control	Tumor	Control	Tumor	Control	Tumor
Control vs. ExomA1AT+	<0.0001	<0.0001	<0.0001	<0.0001	<0.0001	<0.0001	<0.0001	<0.0001	0.0005	0.0005	<0.0001	<0.0001
Control vs. ExomA1AT-	<0.0001	0.3444	0.6412	0.5141	0.7888	<0.0001	0.9858	<0.0001	0.6615	0.6615	<0.0001	0.0028
ExomA1AT+ vs. ExomA1AT-	<0.0001	<0.0001	<0.0001	<0.0001	<0.0001	0.7272	<0.0001	<0.0001	0.0001	0.0001	<0.0001	<0.0001

Supplementary Fig. S23

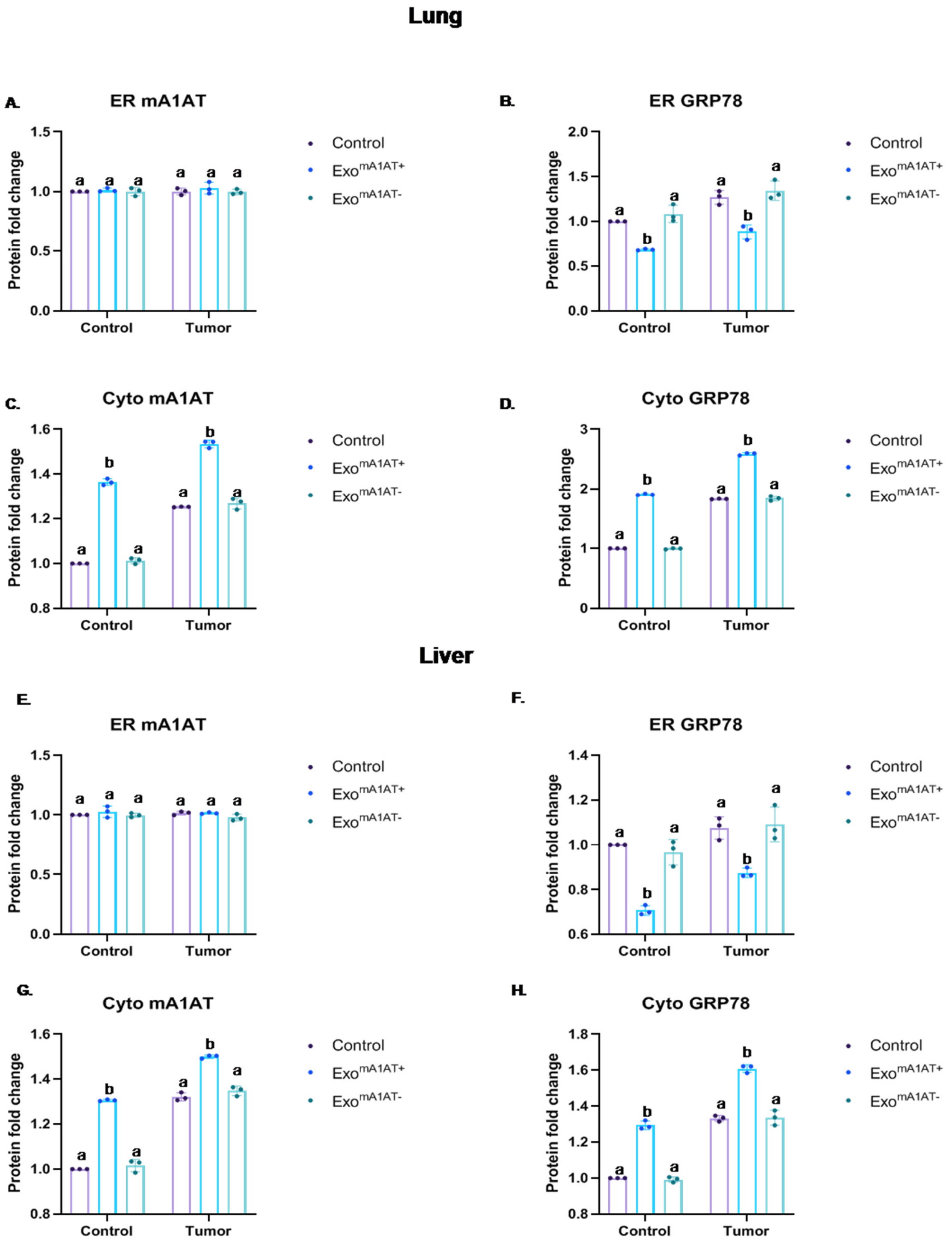

Fig S23. Densitometric quantification of mA1AT and GRP78 in ER and cytoplasmic fraction from lung and liver tissue as shown in Figs. 7A. (A–B) Densitometric analyses of (A) mA1AT and (B) GRP78 expression in the ER fraction of lung as shown in Fig. 7A. (C–D) Densitometric analyses of (C) mA1AT and (D) GRP78 expression in the cytoplasmic fraction of lung as shown in Fig. 7A. (E–F) Densitometric analyses of (E) mA1AT and (F) GRP78 expression in the ER fraction of liver as shown in Fig. 7A. (G–H) Densitometric analyses of (G) mA1AT and (H) GRP78 expression in the cytoplasmic fraction of liver as shown in Fig. 7A. Data are presented as mean ± SD (n=3 biological replicates). Statistical significance was determined using two-way ANOVA followed by Tukey's multiple-comparison test. P < 0.05 was considered statistically significant. Lowercase letters (a,b) indicate statistically significant differences among groups.

Table S23: Adjusted P values corresponding to Fig. S23A-H

	ER mA1AT		ER GRP78		Cyto mA1AT		Cyto GRP78	
Lung	Control	Tumor	Control	Tumor	Control	Tumor	Control	Tumor
Control vs. Exo^{mA1AT+}	0.8747	0.5101	0.0005	<0.0001	<0.0001	<0.0001	<0.0001	<0.0001
Control vs. Exo^{mA1AT-}	0.9998	0.9892	0.3709	0.4552	0.5919	0.4226	0.9688	0.7593
Exo^{mA1AT+} vs. Exo^{mA1AT-}	0.8666	0.4329	<0.0001	<0.0001	<0.0001	<0.0001	<0.0001	<0.0001
	ER mA1AT		ER GRP78		Cyto mA1AT		Cyto GRP78	
Liver	Control	Tumor	Control	Tumor	Control	Tumor	Control	Tumor
Control vs. Exo^{mA1AT+}	0.4038	>0.9999	<0.0001	0.0005	<0.0001	<0.0001	<0.0001	<0.0001
Control vs. Exo^{mA1AT-}	0.9911	0.1879	0.6497	0.9068	0.494	0.1584	0.8958	0.977
Exo^{mA1AT+} vs. Exo^{mA1AT-}	0.3422	0.1914	<0.0001	0.0002	<0.0001	<0.0001	<0.0001	<0.0001

Supplementary Fig. S24

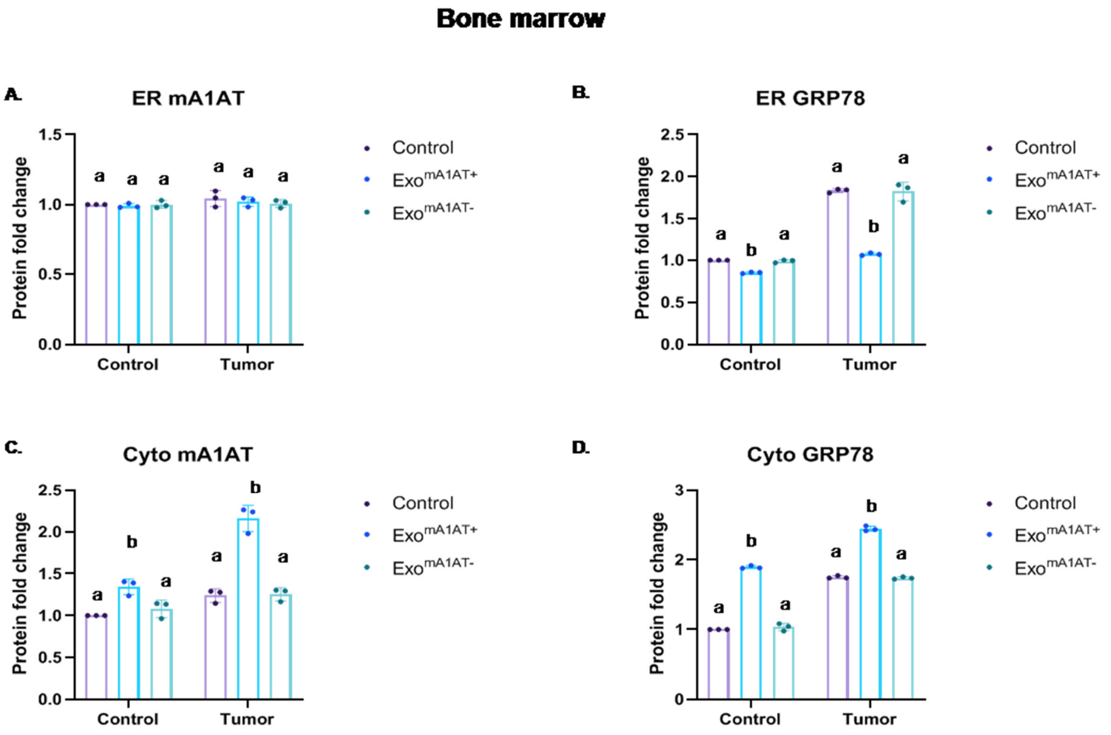

Fig S24. Densitometric quantification of mA1AT and GRP78 in ER and cytoplasmic fraction from bone marrow tissue as shown in Fig. 7A. (A–B) Densitometric analyses of (A) mA1AT and (B) GRP78 expression in the ER fraction of bone marrow as shown in Fig. 7A. (C–D) Densitometric analyses of (C) mA1AT and (D) GRP78 expression in the cytoplasmic fraction of bone marrow as shown in Fig. 7A. Data are presented as mean ± SD (n=3 biological replicates). Statistical significance was determined using two-way ANOVA followed by Tukey's multiple-comparison test. P < 0.05 was considered statistically significant. Lowercase letters (a,b) indicate statistically significant differences among groups.

Table S24: Adjusted P values corresponding to Fig. S24A-D

BM	ER mA1AT		ER GRP78		Cyto mA1AT		Cyto GRP78	
	Control	Tumor	Control	Tumor	Control	Tumor	Control	Tumor
Control vs. ExomA1AT+	0.9404	0.7037	0.0061	<0.0001	0.0025	<0.0001	<0.0001	<0.0001
Control vs. ExomA1AT-	0.9992	0.3878	0.9647	0.9546	0.5767	0.9777	0.3268	0.8815
ExomA1AT+ vs. ExomA1AT-	0.9271	0.8453	0.0097	<0.0001	0.0156	<0.0001	<0.0001	<0.0001

Supplementary Fig. S25

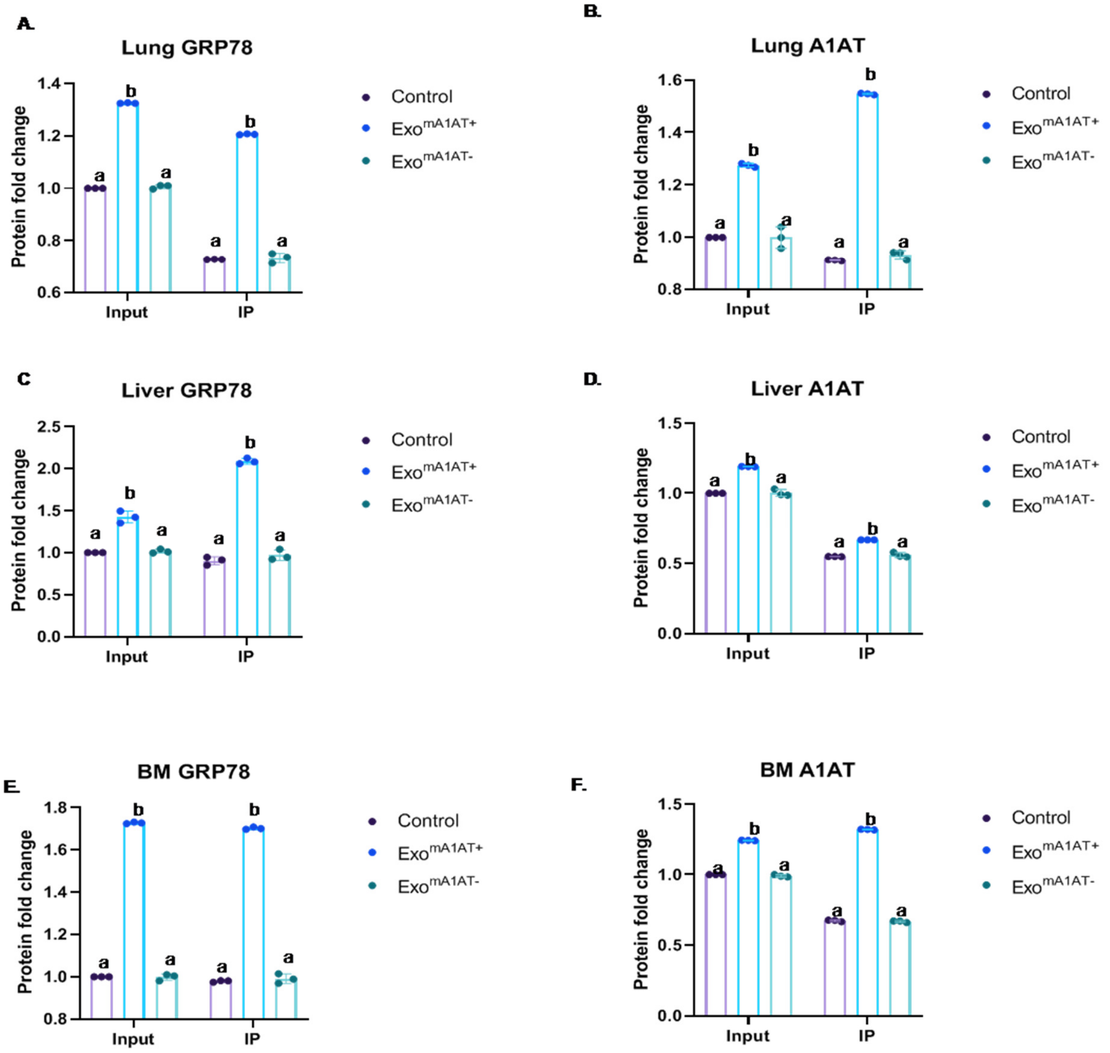

Figure S25. Densitometric quantification of co-immunoprecipitation analyses of GRP78 and A1AT in cytoplasmic fractions shown in Fig. 7C. (A–F) Quantitative densitometric analyses of GRP78 and mA1AT expression in the cytoplasmic fractions of lung (A, B), liver (C, D), and bone marrow (E, F) tissues obtained from the non-tumor-bearing mice of the in vivo exosome education study, corresponding to the co-immunoprecipitation data shown in Fig. 7C. Data are presented as mean ± SD (n=3 biological replicates per group). Statistical significance was determined using two-way ANOVA followed by Tukey's multiple-comparison test. $P < 0.05$ was considered statistically significant. Different lowercase letters (a, b) indicate statistically significant differences among groups.

Table S25: Adjusted P values corresponding to Fig. S25A-F

	Control			
	Cyto GRP78:A1AT		Cyto GRP78:GRP78	
Lung	Input	IP	Input	IP
Control vs. ExomA1AT+	<0.0001	<0.0001	<0.0001	<0.0001
Control vs. ExomA1AT-	0.9964	0.4039	0.6878	0.6959
ExomA1AT+ vs. ExomA1AT-	<0.0001	<0.0001	<0.0001	<0.0001
	Control			
	Cyto GRP78:A1AT		Cyto GRP78:GRP78	
Liver	Input	IP	Input	IP
Control vs. ExomA1AT+	<0.0001	<0.0001	<0.0001	<0.0001
Control vs. ExomA1AT-	0.9599	0.5785	0.9029	0.2368
ExomA1AT+ vs. ExomA1AT-	<0.0001	<0.0001	<0.0001	<0.0001
	Control			
	Cyto GRP78:A1AT		Cyto GRP78:GRP78	
BM	Input	IP	Input	IP
Control vs. ExomA1AT+	<0.0001	<0.0001	<0.0001	<0.0001
Control vs. ExomA1AT-	0.1234	0.3651	0.9749	0.4473
ExomA1AT+ vs. ExomA1AT-	<0.0001	<0.0001	<0.0001	<0.0001

Supplementary Fig. S26

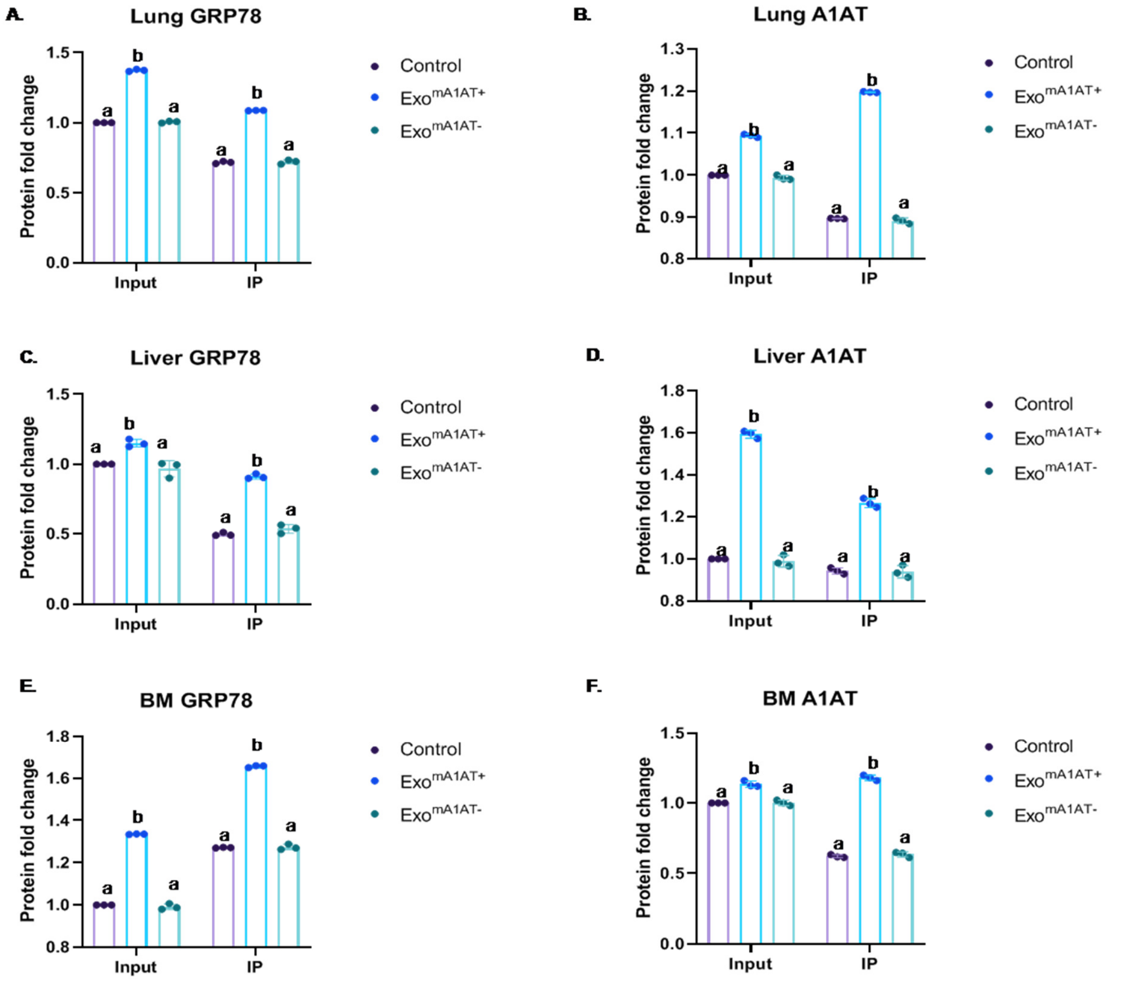

Figure S26. Densitometric quantification of co-immunoprecipitation analyses of GRP78 and A1AT in cytoplasmic fractions shown in Fig. 7D. (A–F) Quantitative densitometric analyses of GRP78 and mA1AT expression in the cytoplasmic fractions of lung (A, B), liver (C, D) and bone marrow (E, F) tissues obtained from the tumor-bearing mice of the in vivo exosome education study, corresponding to the co-immunoprecipitation data shown in Fig. 7I. Data are presented as mean ± SD (n=3 biological replicates per group). Statistical significance was determined using two-way ANOVA followed by Tukey's multiple-comparison test. $P < 0.05$ was considered statistically significant. Different lowercase letters (a, b) indicate statistically significant differences among groups.

Table S26: Adjusted P values corresponding to Fig. S26A-F

	Tumor			
	Cyto GRP78:A1AT		Cyto GRP78:GRP78	
Lung	Input	IP	Input	IP
Control vs. ExomA1AT+	<0.0001	<0.0001	<0.0001	<0.0001
Control vs. ExomA1AT-	0.1751	0.2558	0.6668	0.7975
ExomA1AT+ vs. ExomA1AT-	<0.0001	<0.0001	<0.0001	<0.0001
	Tumor			
	Cyto GRP78:A1AT		Cyto GRP78:GRP78	
Liver	Input	IP	Input	IP
Control vs. ExomA1AT+	<0.0001	<0.0001	0.0002	<0.0001
Control vs. ExomA1AT-	0.7698	0.9724	0.3977	0.3042
ExomA1AT+ vs. ExomA1AT-	<0.0001	<0.0001	<0.0001	<0.0001
	Tumor			
	Cyto GRP78:A1AT		Cyto GRP78:GRP78	
BM	Input	IP	Input	IP
Control vs. ExomA1AT+	<0.0001	<0.0001	<0.0001	<0.0001
Control vs. ExomA1AT-	0.9973	0.6074	0.3647	0.9207
ExomA1AT+ vs. ExomA1AT-	<0.0001	<0.0001	<0.0001	<0.0001

Supplementary Fig. S27

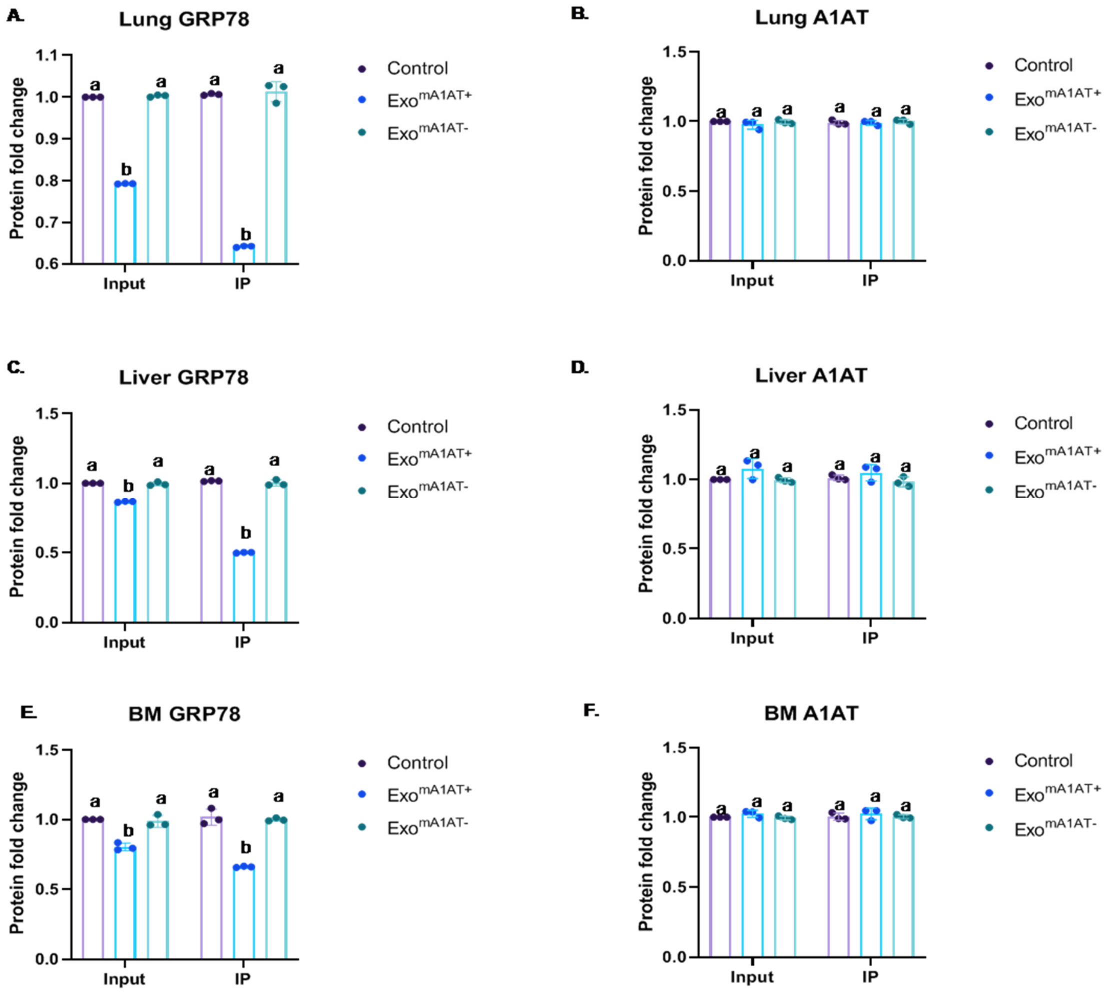

Figure S27. Densitometric quantification of co-immunoprecipitation analyses of GRP78 and A1AT in cytoplasmic fractions shown in Fig. 7E. (A–F) Quantitative densitometric analyses of GRP78 and mA1AT expression in the ER fractions of lung (A, B), liver (C, D) and bone marrow (E, F) tissues obtained from the non-tumor-bearing mice of the in vivo exosome education study, corresponding to the co-immunoprecipitation data shown in Fig. 7E. Data are presented as mean ± SD (n=3 biological replicates per group). Statistical significance was determined using two-way ANOVA followed by Tukey's multiple-comparison test. $P < 0.05$ was considered statistically significant. Different lowercase letters (a, b) indicate statistically significant differences among groups.

Table S27: Adjusted P values corresponding to Fig. S27A-F

	Control			
	ER GRP78:A1AT		ER GRP78:GRP78	
Lung	Input	IP	Input	IP
Control vs. ExomA1AT+	0.3469	0.9928	<0.0001	<0.0001
Control vs. ExomA1AT-	0.9713	0.8541	0.9377	0.7153
ExomA1AT+ vs. ExomA1AT-	0.4633	0.7942	<0.0001	<0.0001
	Control			
	ER GRP78:A1AT		ER GRP78:GRP78	
Liver	Input	IP	Input	IP
Control vs. ExomA1AT+	0.0906	0.5324	<0.0001	<0.0001
Control vs. ExomA1AT-	0.9887	0.6739	0.8469	0.2633
ExomA1AT+ vs. ExomA1AT-	0.0708	0.1646	<0.0001	<0.0001
	Control			
	ER GRP78:A1AT		ER GRP78:GRP78	
BM	Input	IP	Input	IP
Control vs. ExomA1AT+	0.4305	0.5958	<0.0001	<0.0001
Control vs. ExomA1AT-	0.9504	>0.9999	0.8684	0.7857
ExomA1AT+ vs. ExomA1AT-	0.288	0.5939	<0.0001	<0.0001

Supplementary Fig. S28

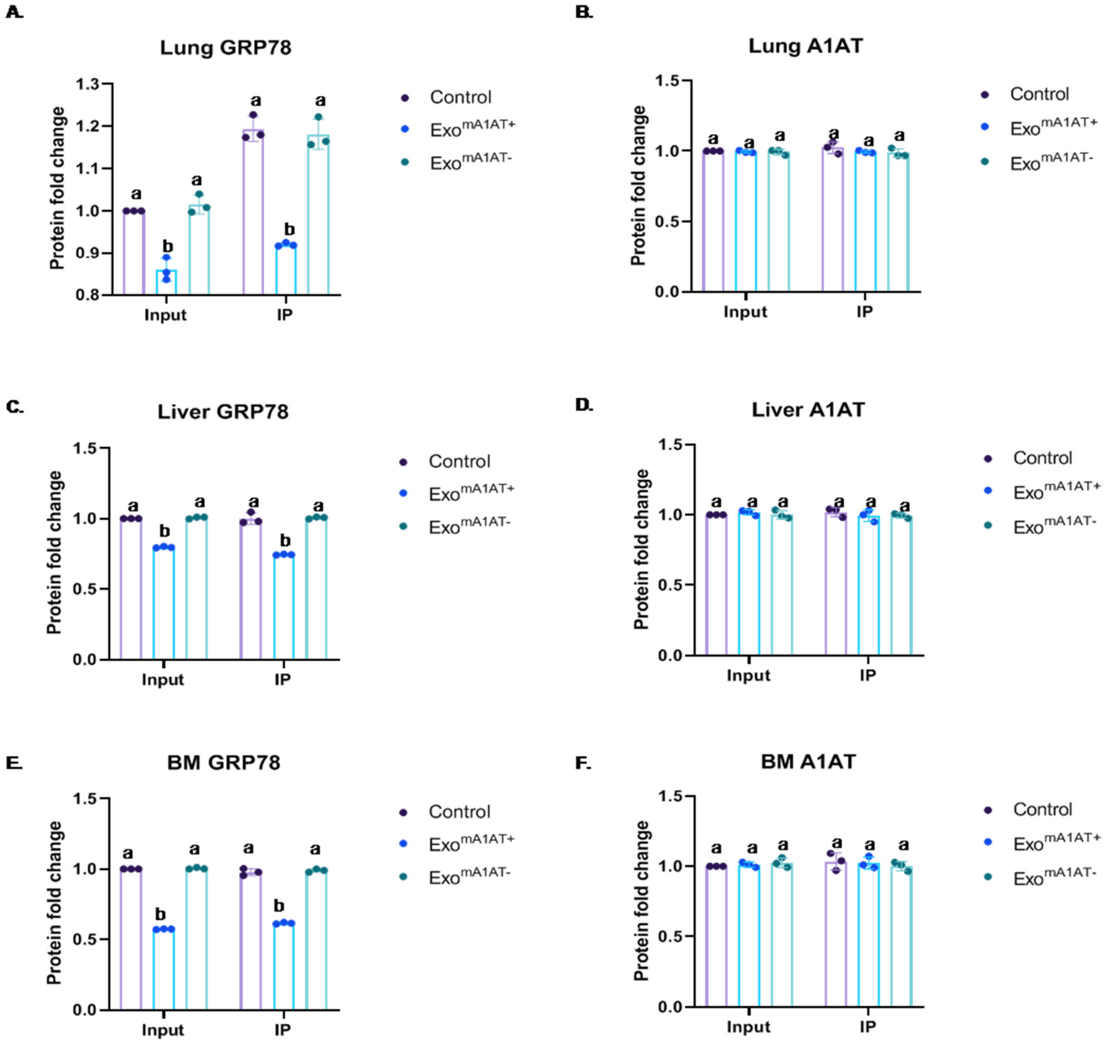

Figure S28. Densitometric quantification of co-immunoprecipitation analyses of GRP78 and A1AT in cytoplasmic fractions shown in Fig. 7F. (A–F) Quantitative densitometric analyses of GRP78 and mA1AT expression in the ER fractions of lung (A, B), liver (C, D) and bone marrow (E, F) tissues obtained from the tumor-bearing mice of the in vivo exosome education study, corresponding to the co-immunoprecipitation data shown in Fig. 7F. Data are presented as mean ± SD (n=3 biological replicates per group). Statistical significance was determined using two-way ANOVA followed by Tukey's multiple-comparison test. $P < 0.05$ was considered statistically significant. Different lowercase letters (a, b) indicate statistically significant differences among groups.

Table S28: Adjusted P values corresponding to Fig. S28A-F

Lung	Tumor			
	ER GRP78:A1AT		ER GRP78:GRP78	
	Input	IP	Input	IP
Control vs. ExomA1AT+	0.9659	0.3007	<0.0001	<0.0001
Control vs. ExomA1AT-	0.9298	0.1547	0.7312	0.8027
ExomA1AT+ vs. ExomA1AT-	0.9929	0.8987	<0.0001	<0.0001

Liver	Tumor			
	ER GRP78:A1AT		ER GRP78:GRP78	
	Input	IP	Input	IP
Control vs. ExomA1AT+	0.7299	0.4956	<0.0001	<0.0001
Control vs. ExomA1AT-	0.9993	0.5117	0.8987	0.912
ExomA1AT+ vs. ExomA1AT-	0.7508	0.9996	<0.0001	<0.0001

BM	Tumor			
	ER GRP78:A1AT		ER GRP78:GRP78	
	Input	IP	Input	IP
Control vs. ExomA1AT+	0.9271	0.9476	<0.0001	<0.0001
Control vs. ExomA1AT-	0.6894	0.5361	0.8726	0.4543
ExomA1AT+ vs. ExomA1AT-	0.8895	0.7219	<0.0001	<0.0001

Supplementary Fig. S29

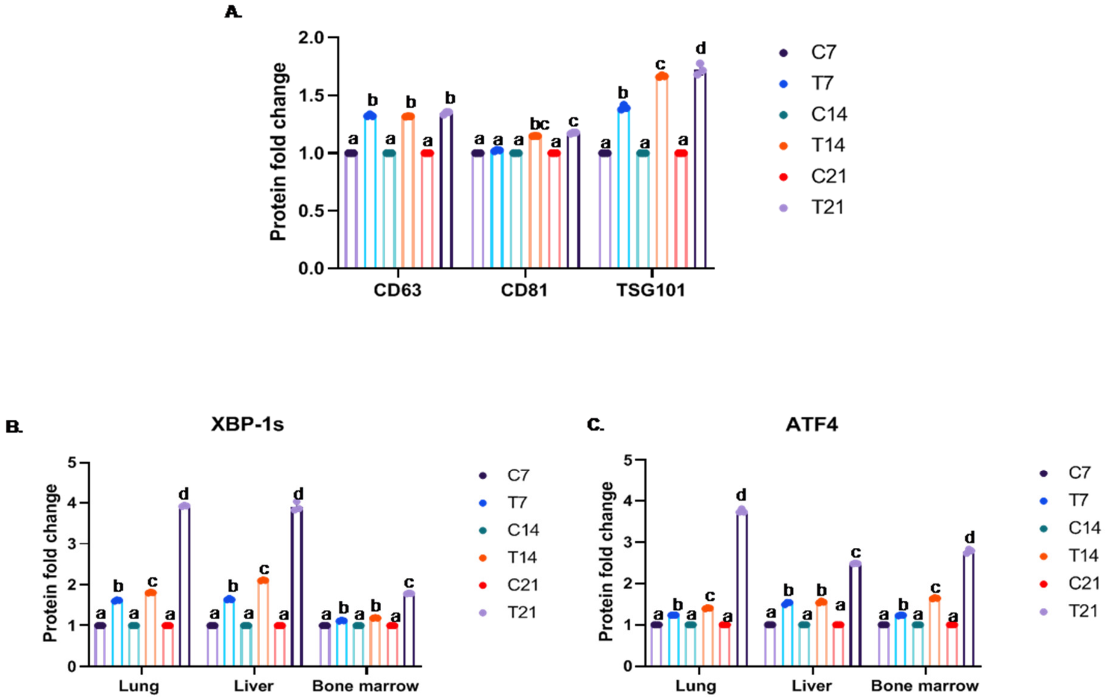

Figure S29. Densitometric quantification of canonical exosomal markers and downstream UPR markers shown in Fig. S1D and S1H. (A) Densitometric analyses of CD63, CD81 and TSG101 expression in serum-derived exosomes isolated from control and tumor-bearing mice at different stages of tumor progression, corresponding to the immunoblot data shown in Fig. S1D. (B, C) Densitometric analyses of the downstream UPR markers (B) XBP-1s and (C) ATF4 in lung, liver and bone marrow cells corresponding to the immunoblot data shown in Fig. S1H. Data are presented as mean ± SD (n=3 biological replicates). Statistical significance was determined using two-way ANOVA followed by Tukey's multiple-comparison test. $P < 0.05$ was considered statistically significant. Different lowercase letters (a-d) indicate statistically significant differences among groups.

Table S29A: Adjusted P values corresponding to Fig. S29A

	CD63	CD81	TSG101
C7 vs. T7	<0.0001	0.2521	<0.0001
C7 vs. C14	>0.9999	>0.9999	>0.9999
C7 vs. T14	<0.0001	<0.0001	<0.0001
C7 vs. C21	>0.9999	>0.9999	>0.9999
C7 vs. T21	<0.0001	<0.0001	<0.0001
T7 vs. C14	<0.0001	0.2521	<0.0001
T7 vs. T14	0.9831	<0.0001	<0.0001
T7 vs. C21	<0.0001	0.2521	<0.0001
T7 vs. T21	0.3529	<0.0001	<0.0001
C14 vs. T14	<0.0001	<0.0001	<0.0001
C14 vs. C21	>0.9999	>0.9999	>0.9999
C14 vs. T21	<0.0001	<0.0001	<0.0001
T14 vs. C21	<0.0001	<0.0001	<0.0001
T14 vs. T21	0.1008	0.1599	0.0001
C21 vs. T21	<0.0001	<0.0001	<0.0001

Table S29B: Adjusted P values corresponding to Fig. S29B-C

	XBP-1s			ATF4		
	Lung	Liver	BM	Lung	Liver	BM
C7 vs. T7	<0.0001	<0.0001	<0.0001	<0.0001	<0.0001	<0.0001
C7 vs. C14	>0.9999	>0.9999	>0.9999	>0.9999	>0.9999	>0.9999
C7 vs. T14	<0.0001	<0.0001	<0.0001	<0.0001	<0.0001	<0.0001
C7 vs. C21	>0.9999	>0.9999	>0.9999	>0.9999	>0.9999	>0.9999
C7 vs. T21	<0.0001	<0.0001	<0.0001	<0.0001	<0.0001	<0.0001
T7 vs. C14	<0.0001	<0.0001	<0.0001	<0.0001	<0.0001	<0.0001
T7 vs. T14	<0.0001	<0.0001	0.0545	<0.0001	0.5177	<0.0001
T7 vs. C21	<0.0001	<0.0001	<0.0001	<0.0001	<0.0001	<0.0001
T7 vs. T21	<0.0001	<0.0001	<0.0001	<0.0001	<0.0001	<0.0001
C14 vs. T14	<0.0001	<0.0001	<0.0001	<0.0001	<0.0001	<0.0001
C14 vs. C21	>0.9999	>0.9999	>0.9999	>0.9999	>0.9999	>0.9999
C14 vs. T21	<0.0001	<0.0001	<0.0001	<0.0001	<0.0001	<0.0001
T14 vs. C21	<0.0001	<0.0001	<0.0001	<0.0001	<0.0001	<0.0001
T14 vs. T21	<0.0001	<0.0001	<0.0001	<0.0001	<0.0001	<0.0001
C21 vs. T21	<0.0001	<0.0001	<0.0001	<0.0001	<0.0001	<0.0001

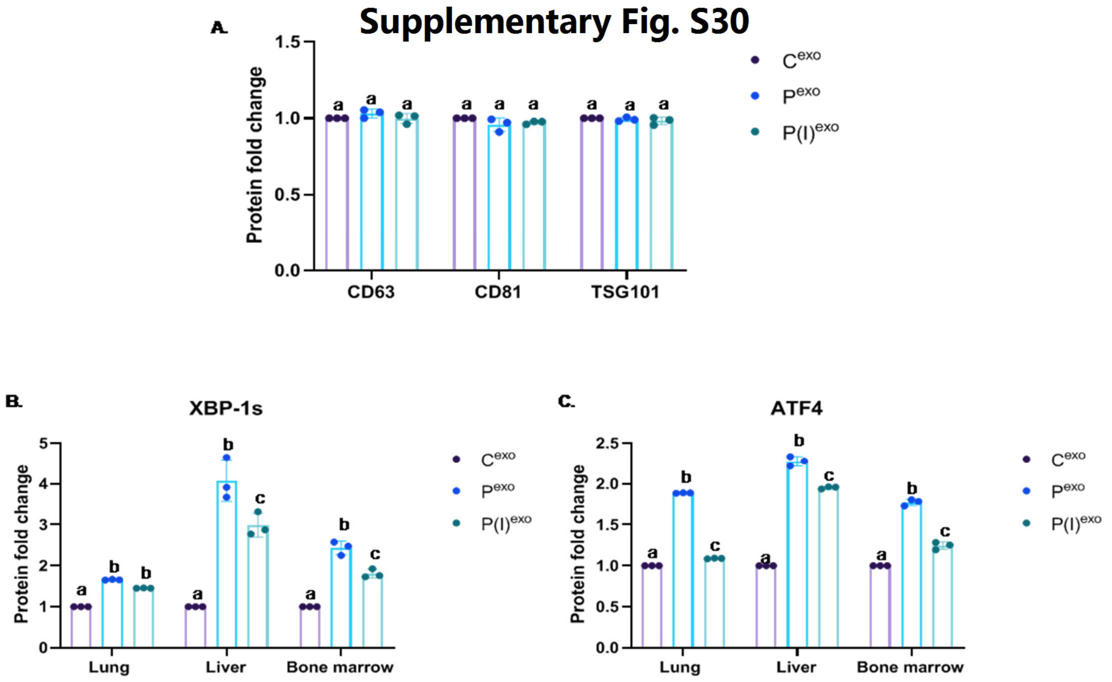

Figure S30. Densitometric quantification of canonical exosomal markers and downstream UPR markers shown in Fig. S2C and S2J. (A) Densitometric analyses of CD63, CD81 and TSG101 expression in exosomes isolated from the conditioned media of untransfected control 4T1cells (C^{exo}), 4T1 cells transfected with empty pcDNA3.1 vector (P^{exo}) and 4T1 cells transfected with pcDNA3.1 vector containing the EDEM1 gene ($P(I)^{exo}$) corresponding to the immunoblot data shown in Fig. S2C. (B, C) Densitometric analyses of the downstream UPR markers (B) XBP-1s and (C) ATF4 in lung, liver and bone marrow cells corresponding to the immunoblot data shown in Fig. S2J. Data are presented as mean ± SD (n=3 biological replicates). Statistical significance was determined using two-way ANOVA followed by Tukey's multiple-comparison test. $P < 0.05$ was considered statistically significant. Different lowercase letters (a-c) indicate statistically significant differences among groups.

Table S30A: Adjusted P values corresponding to Fig. S30A

	CD63	CD81	TSG101
C^{exo} vs. P^{exo}	0.2336	0.0871	0.9241
C^{exo} vs. $P(I)^{exo}$	0.9961	0.3309	0.6119
P^{exo} vs. $P(I)^{exo}$	0.2039	0.7075	0.833

Table S30B: Adjusted P values corresponding to Fig. S30B-C

Fig. S2J	XBP-1s			ATF4		
	Lung	Liver	BM	Lung	Liver	BM
C^{exo} vs. P^{exo}	0.0028	<0.0001	<0.0001	<0.0001	<0.0001	<0.0001
C^{exo} vs. $P(I)^{exo}$	0.0367	<0.0001	0.0004	0.0032	<0.0001	<0.0001
P^{exo} vs. $P(I)^{exo}$	0.4656	<0.0001	0.0042	<0.0001	<0.0001	<0.0001

Supplementary Fig. S31

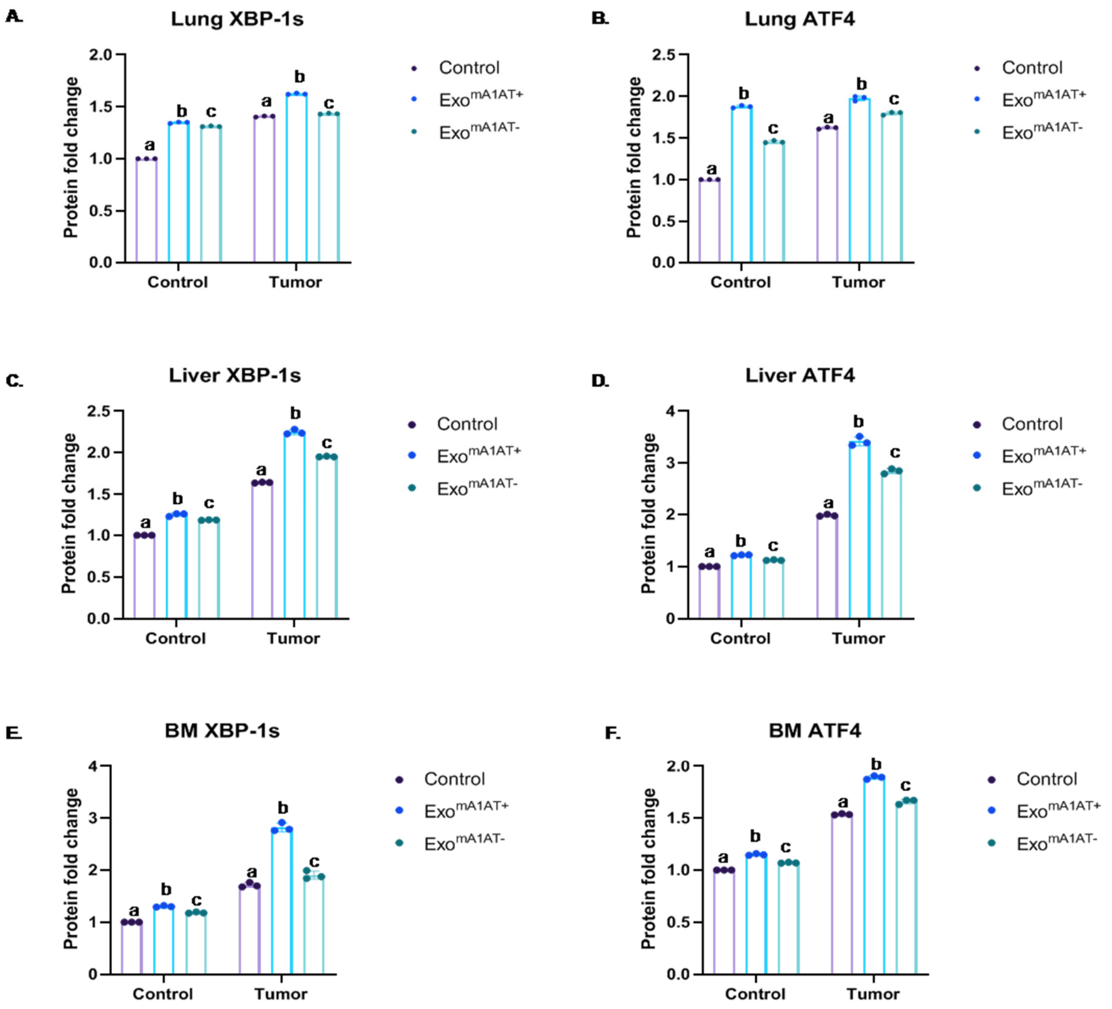

Figure S31. Densitometric quantification of downstream UPR markers shown in Fig. S2K. (A-F) Densitometric analyses of XBP-1s and ATF4 expression in (A,B) lung, (C,D) liver and (E,F) bone marrow respectively, obtained from the exosome education study, corresponding to the immunoblot data shown in Fig. S2K. Data are presented as mean ± SD (n=3 biological replicates). Statistical significance was determined using two-way ANOVA followed by Tukey's multiple-comparison test. P < 0.05 was considered statistically significant. Different lowercase letters (a-c) indicate statistically significant differences among groups.

Table S31: Adjusted P values corresponding to Fig. S31A-F

	XBP-1s		ATF4	
Lung	Control	Tumor	Control	Tumor
Control vs. Exo^{mA1AT+}	<0.0001	<0.0001	<0.0001	<0.0001
Control vs. Exo^{mA1AT-}	<0.0001	0.0001	<0.0001	<0.0001
Exo^{mA1AT+} vs. Exo^{mA1AT-}	<0.0001	<0.0001	<0.0001	<0.0001
	XBP-1s		ATF4	
Liver	Control	Tumor	Control	Tumor
Control vs. Exo^{mA1AT+}	<0.0001	<0.0001	<0.0001	<0.0001
Control vs. Exo^{mA1AT-}	<0.0001	<0.0001	0.0098	<0.0001
Exo^{mA1AT+} vs. Exo^{mA1AT-}	0.0005	<0.0001	0.0334	<0.0001
	XBP-1s		ATF4	
BM	Control	Tumor	Control	Tumor
Control vs. Exo^{mA1AT+}	<0.0001	<0.0001	<0.0001	<0.0001
Control vs. Exo^{mA1AT-}	0.0023	0.0018	<0.0001	<0.0001
Exo^{mA1AT+} vs. Exo^{mA1AT-}	0.0349	<0.0001	<0.0001	<0.0001
